# Factors associated with severe neurological sequelae of COVID-19: findings from the multicenter COVID-BRAIN imaging cohort

**DOI:** 10.3389/fnhum.2026.1754342

**Published:** 2026-03-19

**Authors:** Ana I. Silva, Keenan Christopher Byrne, Lauren Pollak, Katherine Gundry, Georgios E. Manousakis, Abby I. Metzler, Christophe Lenglet, Lynn E. Eberly, June Kendall-Thomas, Orhun H. Kantarci, Burcu Zeydan, Shibani S. Mukerji, Sevil Yasar, Tetsuo Ashizawa, Kejal Kantarci, Eva-Maria Ratai, Gülin Öz, COVID-BRAIN Investigators, James M. Joers, Alfredo Lorente, Jeromy Thotland, Jaime Lavallee, Dinesh K. Deelchand, Young Woo Park, Xiufeng Li, Merve Atik, Matthew L. Senjem, Meher R. Juttukonda, David H. Salat, Janet C. Sherman, G. Kyle Harrold, Mehreen Nabi, Sana Rehman, Ipek Özdemir, Dillip Senapati, Peter B. Barker, Christof Karmonik, Syed A. Gillani, Valerie Flores, Rachel Davis

**Affiliations:** 1 Department of Radiology, Center for Magnetic Resonance Research, University of Minnesota, Minneapolis, MN, United States; 2 Department of Radiology, Athinoula A. Martinos Center for Biomedical Imaging, Massachusetts General Hospital, Charlestown, MA, United States; 3 Department of Psychiatry, Psychology Assessment Center, Massachusetts General Hospital, Harvard Medical School, Boston, MA, United States; 4 Department of Neurology, University of Minnesota, Minneapolis, MN, United States; 5 Division of Biostatistics and Health Data Science, School of Public Health, University of Minnesota, Minneapolis, MN, United States; 6 Department of Radiology, Mayo Clinic Rochester, Rochester, MN, United States; 7 Department of Neurology, Mayo Clinic Rochester, Rochester, MN, United States; 8 Harvard Medical School, Boston, MA, United States; 9 Department of Neurology, Massachusetts General Hospital, Boston, MA, United States; 10 Johns Hopkins University, Baltimore, MD, United States; 11 Stanley Appel Department of Neurology, Houston Methodist Research Institute, Weill Cornell Medicine, Houston, TX, United States

**Keywords:** blood biomarkers, long COVID, neurocognitive impairment, post-acute COVID-19, SARS-CoV-2

## Abstract

**Introduction:**

Neurological post-acute sequelae of COVID-19 (neuroPASC) are associated with persistent cognitive dysfunction and quality-of-life decline. We aimed to identify clinical, behavioral and sociodemographic factors associated with neuroPASC symptom burden two years after COVID-19 among individuals without prior neurological disease.

**Methods:**

In this prospective, observational study, individuals with neuroPASC (*n* = 102) and controls without symptomatic COVID-19 (*n* = 74), all without prior neurological, psychiatric, or post-viral conditions, were enrolled between February 2022 and June 2024 across five academic sites. An unsupervised algorithm identified clusters with differing self-reported neurological symptom burden within the neuroPASC group. Functional differences between clusters were evaluated using quality-of-life, neurological and cognitive evaluations. Demographics, behavioral history, comorbidities, and blood biomarkers were compared across clusters and controls. Multivariable logistic regression assessed predictors of neuroPASC severity, including demographics, body-mass-index, Charlson Comorbidity Index, Framingham Risk Score, pre-existing endocrine/metabolic and/or gastrointestinal/hepatobiliary conditions, COVID-19 vaccination prior to infection, hospitalization during acute infection, and cumulative alcohol use.

**Results:**

Two clusters emerged based on neurological symptom burden, labeled “high-burden” and “low-burden” neuroPASC, reflecting differences in the number and frequency of symptoms. Both clusters had deficits in quality-of-life and cognitive function compared to controls, with greater impairment in high-burden than low-burden neuroPASC. The clusters did not differ by sex, education, tobacco and cannabis use, blood pressure, body-mass-index, HbA1C, days since infection, hospitalization during COVID-19, pre-COVID vaccination rate, antibody-positivity, inflammation, and neurodegeneration biomarkers. The high-burden cluster was older and exhibited higher comorbidity burden and greater cumulative alcohol use compared with the low-burden cluster and controls. Pre-existing endocrine/metabolic and gastrointestinal/hepatobiliary conditions were more common in high-burden (63%) than in low-burden neuroPASC (35%). After adjusting for clinical and demographic factors, these pre-existing conditions remained the only independent predictor of severity, conferring a 3.5-fold increase in the odds of high-burden versus low-burden neuroPASC.

**Discussion:**

Older age, higher comorbidity burden, greater cumulative alcohol use, and endocrine/metabolic and gastrointestinal conditions, rather than acute COVID-19 severity, were observed in the high-burden neuroPASC cluster. After multivariable adjustment, only pre-existing endocrine/metabolic and/or gastrointestinal/hepatobiliary conditions remained independently associated with high-burden neuroPASC, conferring a 3.5-fold increase in odds and highlighting the need for targeted post-infection monitoring in at-risk patients.

## Introduction

Long COVID is a static, relapsing and remitting, or progressive disease that occurs after SARS-CoV-2 infection, with a minimum duration of 3 months (National Academies of Sciences, Engineering, and Medicine, 2024). Neurological post-acute sequelae of COVID-19 (neuroPASC) refer specifically to enduring neurological and cognitive symptoms (Mina et al., 2023), and is associated with persistent cognitive dysfunction and quality-of-life decline (Katz et al., 2023; Kim et al., 2023; Szewczyk et al., 2024). The most affected domains reported in neuroPASC are executive function, memory, attention, and processing speed (Taquet et al., 2021; Guo et al., 2022; Ballouz et al., 2023; Cheetham et al., 2023; Panagea et al., 2025), with some individuals continuing to show cognitive dysfunction 2 years after acute COVID-19 (Ballouz et al., 2023). Cognitive recovery usually parallels the resolution of neurological symptoms (Guo et al., 2022; Ballouz et al., 2023; Cheetham et al., 2023).

Several studies have investigated risk factors for long COVID. Overall, older age, certain comorbidities (particularly cardiovascular disease), hospitalization during acute infection, higher body mass index (BMI), and female sex have consistently been reported as risk factors for post-acute sequelae of COVID-19 (Pilotto et al., 2021; Sudre et al., 2021; Tsampasian et al., 2023; Romero-Ibarguengoitia et al., 2024; Alcalde-Herraiz et al., 2025; Hou et al., 2025). In contrast, relatively few studies have specifically examined risk factors associated with persistent neurological symptoms, and findings across these studies have been variable or cohort-specific (Frontera and Simon, 2022; Li et al., 2023; Cahan et al., 2024; Choudhury et al., 2025; Shil et al., 2025). Notably, many studies recruit participants from specialized clinics, potentially excluding individuals with mild or transient symptoms who are less likely to seek specialized care (Exley et al., 2025). Pre-existing major neurological or psychiatric conditions also pose potential confounders, as they can independently affect cognition, quality-of-life and neurological function, and evidence suggests that such symptoms can worsen within two to three years following COVID-19 hospitalization (Taquet et al., 2024) and potentially even after mild-to-moderate infection (Jose, 2024). Moreover, most neurocognitive assessments in long COVID research have focused on previously hospitalized individuals, many of whom required mechanical ventilation as part of their treatment (Panagea et al., 2025). Consequently, the risk factors associated with neuroPASC symptom burden among individuals without prior neurological or psychiatric disorders—and without the confounding influence of mechanical ventilation—remain poorly understood.

The multi-site COVID-Brain Advanced Imaging Network (COVID-BRAIN) (COVID-BRAIN Project, 2021) seeks to elucidate the long-term effects of SARS-CoV-2 infection on the brain through advanced magnetic resonance imaging (MRI), standardized neurological and neuropsychological assessments, and blood-based biomarkers. The neuroPASC group in this study consisted of individuals with no prior history of chronic neurological or active psychiatric disorders, who had not required mechanical ventilation during acute COVID-19, and who continued to experience neurological symptoms two years post-infection. Therefore, the COVID-BRAIN cohort allowed us to examine factors associated with neuroPASC symptom burden without the confounding influence of pre-existing major neurological or psychiatric conditions and from complications associated with mechanical ventilation. Using an unsupervised cluster analysis of self-reported neurological symptoms, we defined neuroPASC sub-groups based on disease burden. We then evaluated daily functioning in these sub-groups using standardized quality-of-life (fatigue, sleep, depression, anxiety) surveys, a structured neurological examination and a centralized cognitive battery comprised of attention, working memory, processing speed and memory assessments to determine if these standardized measures corroborated self-reported disease burden. Finally, we investigated the factors associated with neuroPASC disease burden, including sex, age, body-mass-index (BMI), need for hospitalization and oxygen therapy during acute infection, behavioral history, social determinants of health, pre-existing comorbidities, presence of nucleocapsid antibodies, and blood biomarkers of inflammation, neurodegeneration and amyloid burden.

## Methods

### Participants

All procedures were approved by the Institutional Review Board: Human Subjects Committee of the University of Minnesota under a central IRB protocol and informed consent was obtained from all participants. COVID-BRAIN (COVID-BRAIN Project, 2021) participants were enrolled between February 2022 and June 2024 across five sites (University of Minnesota, Mayo Clinic Rochester, Massachusetts General Hospital, Johns Hopkins University, Houston Methodist Research Institute), using the following inclusion criteria:

1. Age 18 years or older;
2. Controls: Individuals who had no known SARS-CoV-2 infection;
3. NeuroPASC group: Individuals who had PCR, antibody or antigen confirmed COVID-19 and presented with neurological symptoms in the 6 months after infection, continued to show at least one post-COVID neurological symptom and fit one of the following criteria during the acute phase of the infection: ambulatory with no or mild symptoms, hospitalized but no oxygen therapy, or hospitalized and on oxygen administered via a nasal cannula, mask or non-invasive ventilation, i.e., individuals with WHO Ordinal Scale scores 0–5 (Rubio-Rivas et al., 2022);
4. English or Spanish speaking (based on self-stated primary language);
5. Clear of any contraindications for MRI: including but not limited to claustrophobia, unable to remain still in an MRI scanner for more than 30 min, presence of paramagnetic substances or pacemakers in body, weight over 300 lbs.

Participants were excluded if they had pre-existing chronic neurological conditions (e.g., neurodegenerative disorders, demyelinating and inflammatory central nervous system disorders, cerebrovascular disease and epilepsy), active psychiatric illness (e.g., bipolar disorder, schizophrenia and active substance use disorder excluding cannabis), end-stage renal disease, end-stage liver disease, stroke, brain tumor, brain infection, or traumatic brain injury with loss of consciousness, were diagnosed with another post-viral syndrome or Chronic Fatigue Syndrome before COVID-19, or required mechanical ventilation during hospitalization (WHO Ordinal Scale scores 6–7) (Rubio-Rivas et al., 2022) to avoid the confounding effects of invasive treatment. Participants with well-controlled, treated, or remitted psychiatric conditions—including depression, anxiety, and attention deficit hyperactivity disorder—were allowed to participate.

**Figure 1:**
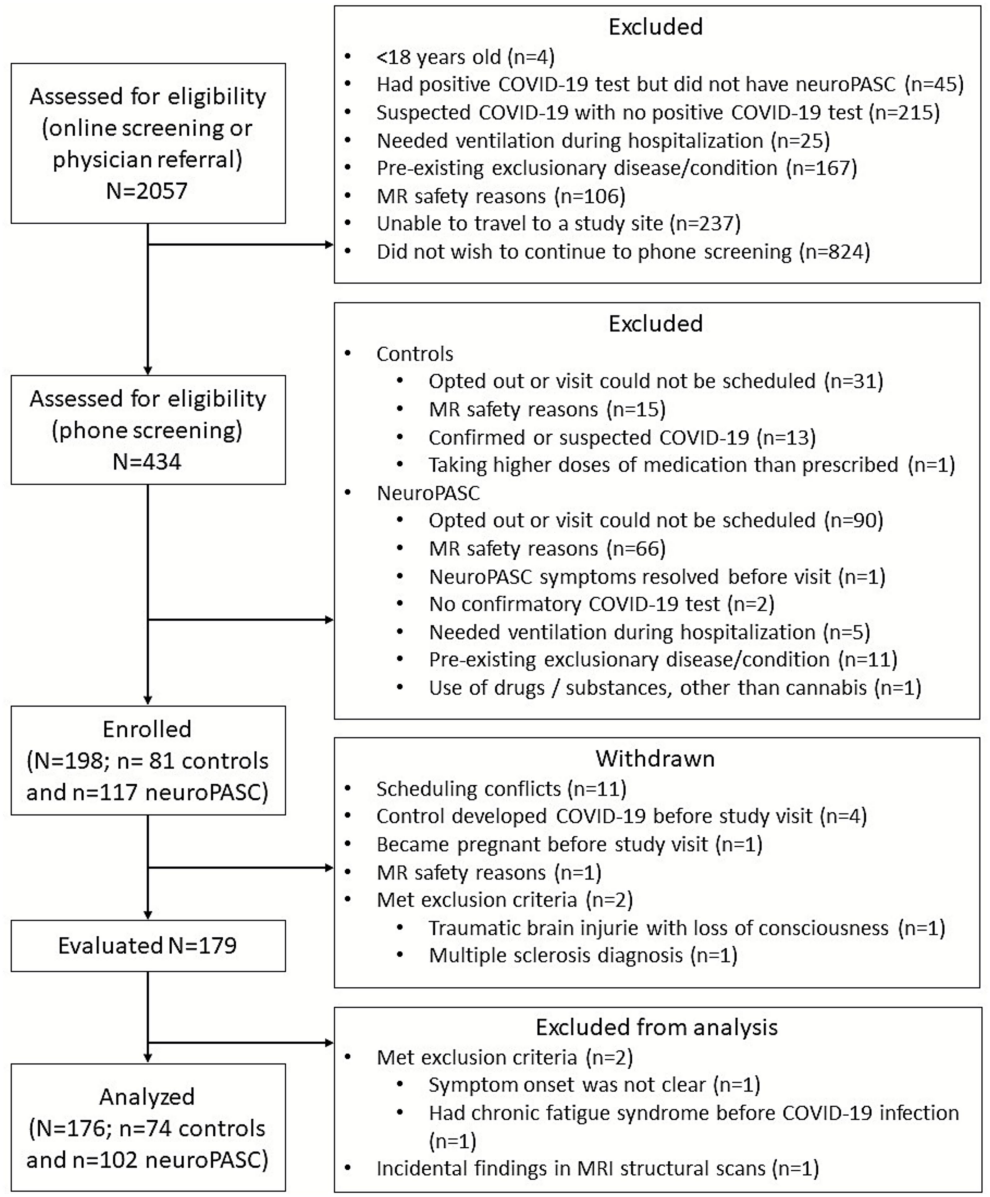
CONSORT diagram. Flow-chart showing how the COVID-BRAIN cohort of individuals with neuroPASC and controls was built. Most interested participants underwent an online screening via REDCap through the study webpage that described study goals and basic eligibility criteria (covidbrainstudy.umn.edu). Interested participants who qualified through the online survey were automatically connected to the coordinator at the nearest enrollment site for a phone screening.

Screening for the study involved two steps (Figure 1): interested participants underwent an online screening (Step 1) via REDCap through the study webpage that described study goals and basic eligibility criteria.^1^ Participants who qualified through the online survey were automatically connected to the coordinator at the nearest enrollment site and underwent a detailed phone screening (Step 2). A total of 117 participants with self-reported neuroPASC (35 of them hospitalized during COVID-19) and 81 control participants were enrolled. Nineteen participants were withdrawn after enrollment (Figure 1), and a total of 179 individuals completed a comprehensive study visit that included MRI acquisition. Three participants were subsequently excluded after the study visit upon discovery that they met exclusion criteria or presented incidental MRI findings likely unrelated to COVID-19. In total, we assessed 176 individuals (neuroPASC *n* = 102, controls *n* = 74; Table 1). All individuals with neuroPASC reported at least one neurological symptom persisting for three or more months, consistent with the long COVID definition developed by the National Academies of Sciences, Engineering, and Medicine (2024).

**Table 1:** Demographics and clinical characteristics of participants with neuroPASC and controls.

Variable	Control participants (*n* = 74)	Participants with NeuroPASC			*p* value neuroPASC vs control	*p* value three group comparison (Control, low-burden, high-burden neuroPASC)
		Total group (*n* = 102)	Low-burden neuroPASC (*n* = 75)	High-burden neuroPASC (*n* = 27)		
Site N						
UMN	32	37	28	9		
Mayo	24	29	24	5		
MGH	17	22	13	9		
JHU	1	11	7	4		
Houston	0	3	3	0		
Age, median (IQR), y	43 (28–59)	50 (35–59)	45 (32–58)	56 (47–64)	0.1^a^	**0.007**^b^, 0.3^c^, **0.003**^d^, **0.005**^e^
Female, No. (%)	47 (63%)	75 (74%)	56 (75%)	20 (74%)	0.1^f^	0.5^g^, 0.5^h^, >0.99^i^
Education, median (IQR), y	16 (16–18)	16 (14–18)	16 (14–18)	16 (14–18)	0.2^a^	0.4^b^, 0.3^c^, 0.3^d^, 0.3^e^
Days since infection, median (IQR), d	NA	781 (504–1,005)	763 (492–950)	859 (583–1,198)	NA	0.1^a^
Hospitalized during COVID-19, No. (%)	NA	25 (25%)	16 (21%)	9 (33%)	NA	0.3^i^
Needed oxygen therapy, No. (%)	NA	22 (22%)	14 (19%)	8 (30%)	NA	0.3^i^
Vaccinated for COVID-19, No. (%)	71 (96%)	94 (92%)	68 (91%)	26 (96%)	0.5^f^	0.7^g^, 0.7^h^, 0.7^i^
Vaccinated for COVID-19 before infection, No. (%)	NA	40 (39%)	30 (40%)	10 (37%)	NA	0.8^i^
BMI, median (IQR)	25 (23–32)	28 (25–36), *n* = 101	29 (25–36)	28 (26–33), *n* = 26	**0.006** ^j^	**0.01**^k^, **0.01**^l^, 0.3^m^, 0.5^n^
HbA1C, %, median (IQR)	5.2 (5–6), *n* = 66	5.3 (5–6), *n* = 79	5.2 (5–6), *n* = 61	5.3 (5–6), *n* = 18	0.1^j^	0.1^k^, 0.1^l^, >0.99^m^, 0.3^n^
Blood pressure (mmHg), median (IQR)						
Systolic	121 (112–130)	122 (110–132)	122 (110–131)	123 (111–134)	0.6^j^	0.6^k^, 0.8^l^, 0.6^m^, 0.6^n^
Diastolic	78 (72–84)	78 (71–83)	78 (71–83)	77 (75–82)	0.8^j^	0.9^k^, 0.8^l^, 0.8^m^, 0.8^n^
CCI, median (IQR)	0 (0–2)	1 (0–2)	0 (0–1)	2 (0–3)	0.3^a^	**0.03**^b^, 0.5^c^, **0.01**^d^, **0.02**^e^
FRS, median (IQR)	3 (1–7), *n* = 73	5 (1–10), *n* = 101	4 (1–10), *n* = 74	7 (3–11)	0.1^a^	**0.02**^b^, 0.3^c^, **0.01**^d^, **0.02**^e^
Positive for SARS-CoV-2 nucleocapsid No. (%)	37 (51%), *n* = 72	94 (93%), *n* = 101	70 (95%), *n* = 74	24 (89%)	**<0.001** ^f^	**<0.001**^g^, **0.001**^h^, 0.4^i^

**Figure 2:**
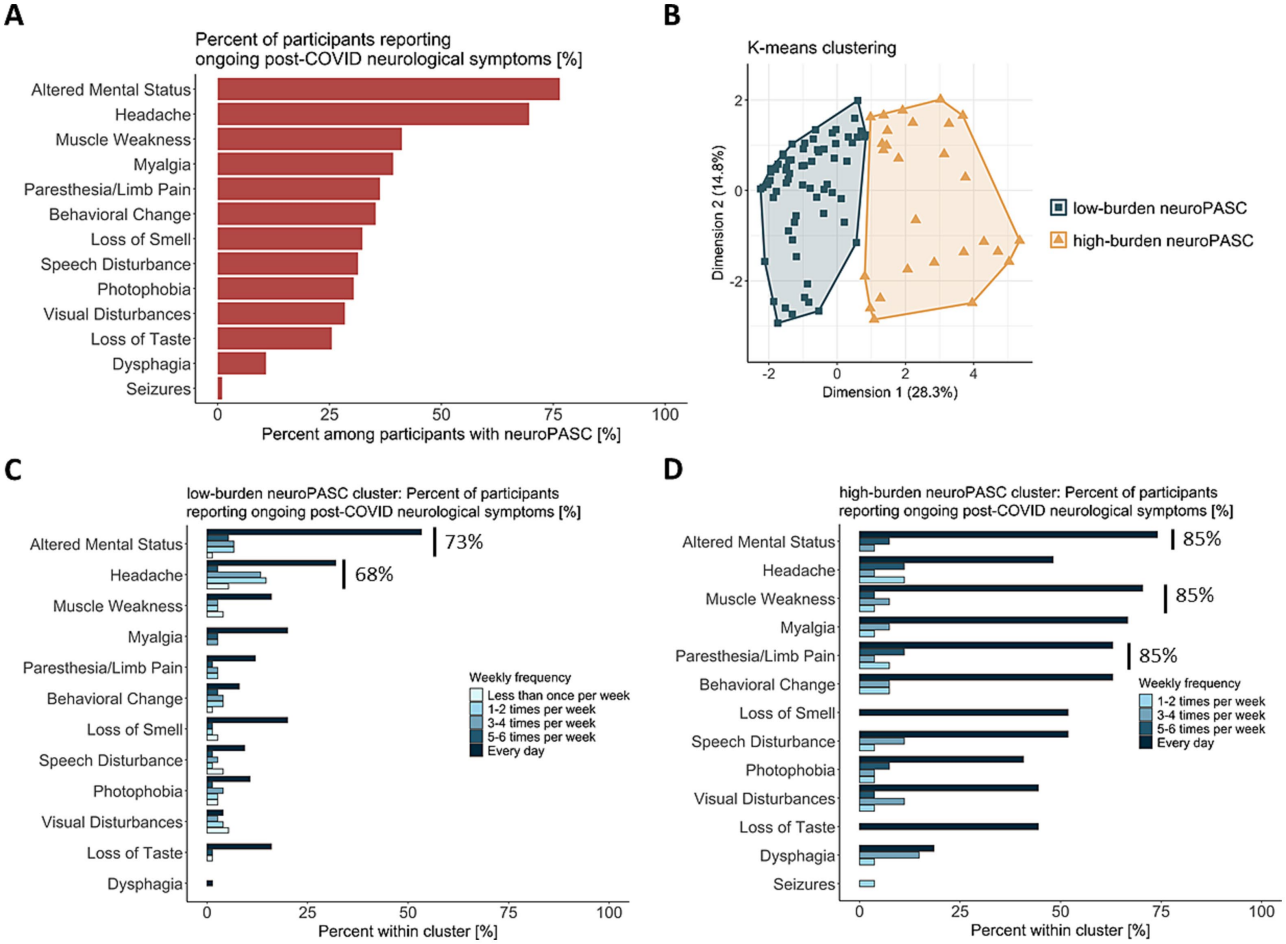
Unsupervised clustering of participants with neuroPASC according to self-reported neurological symptoms that appeared after COVID-19 infection and were ongoing at the time of evaluation. **(A)** Percent of participants with neuroPASC reporting ongoing post-COVID neurological symptoms that were present within 14 days prior to the study visit. **(B)** *K*-means clustering analysis of post-COVID neurological symptoms, revealing 2 distinct clusters of participants with neuroPASC based on their symptom burden. Symptom burden was measured as weekly frequency of each symptom, at the time each symptom was most severe. Cluster designations (“low-burden” and “high-burden”) were created based on visual examination of observed frequencies in panels **(C,D)**. **(C)** Percent of participants reporting ongoing post-COVID neurological symptoms in the low-burden neuroPASC cluster, color-coded by weekly frequency of each symptom. **(D)** Percent of participants reporting ongoing post-COVID neurological symptoms in the high-burden neuroPASC cluster, color-coded by weekly frequency of each symptom.

Participants with neuroPASC were empirically grouped in two distinct clusters, low-burden and high-burden neuroPASC (Figure 2), based on the self-reported burden of neurological symptoms that were ongoing at the time of visit. Group comparisons were conducted using the Benjamini–Hochberg correction for multiple testing (false discovery rate, FDR < 0.05) across the following contrasts: controls vs. low-burden neuroPASC, controls vs. high-burden neuroPASC, and low-burden vs. high-burden neuroPASC. Sample sizes are indicated in cases with missing data. CCI, Charlson Comorbidity Index; FRS, 10-Year Framingham Risk score.

a Mann–Whitney U test.

b Kruskal-Wallis rank sum test.

c Dunn adjusted pairwise comparison, control vs low-burden neuroPASC.

d Dunn adjusted pairwise comparison, control vs high-burden neuroPASC.

e Dunn adjusted pairwise comparison, low-burden vs high-burden neuroPASC.

f Fisher’s exact test.

g Fisher’s exact test, control vs low-burden neuroPASC.

h Fisher’s exact test, control vs high-burden neuroPASC.

i Fisher’s exact test, low-burden vs high-burden neuroPASC.

j ANCOVA 1 degree of freedom F-test, adjusted for age and sex.

k ANCOVA 2 degree of freedom F-test, adjusted for age and sex.

l Pairwise comparison, control vs low-burden neuroPASC, adjusted for age and sex.

m Pairwise comparison, control vs high-burden neuroPASC, adjusted for age and sex.

n Pairwise comparison, low-burden vs high-burden neuroPASC, adjusted for age and sex.Bold values were used to highlight significant *p*-values (*p* < 0.05).

### Self-reported neuroPASC symptoms

NeuroPASC symptoms obtained by a structured interview included altered mental status (reduced level of awareness, confusion, brain fog), headaches, behavioral change, speech disturbances, muscle weakness, myalgia, paresthesia/limb pain, dysphagia, loss of smell, loss of taste, photophobia, visual disturbances, and seizures. The list was adapted from the neurological symptom checklist in the COVID-Neuro Network case record form (Brain Infections Global, 2020), which was developed through expert consensus to facilitate standardized data collection from patients with neurological complications of COVID-19. Participants reported when they experienced the symptom, whether the symptom was ongoing (experienced within the past 14 days), and weekly symptom frequency at its most severe. NeuroPASC symptoms were defined as those neurological symptoms that were either: (1) experienced during and persisting beyond the acute phase; or (2) newly emergent after the acute phase. The case report form that formed the basis of the REDCap symptom survey, which was filled by neurologist co-investigators at each site, is provided in Supplementary Forms (“COVID-BRAIN NEUROLOGICAL SYMPTOMS”).

### Clinical assessment and neurological examination

Quality-of-life questionnaires included Modified Fatigue Impact Scale (MFIS), Pittsburgh Sleep Quality Index (PSQI), Patient Health Questionnaire (PHQ-8), and General Anxiety Disorder (GAD-7). Self-reported medical history was recorded, including endocrine/metabolic, gastrointestinal/hepatobiliary, psychiatric, neurological, cardiovascular, respiratory, musculoskeletal, ocular/vision, renal/urinary, dermatological, blood, reproductive, tumor/cancer, systemic or other conditions. A structured neurological exam (see Supplementary Forms “COVID-BRAIN NEUROLOGICAL EXAM”) was administered, including meningeal signs, mental status, cranial nerves, motor functions, sensation, coordination, reflexes and gait (Campbell, 2012).

### Neurocognitive assessment

A battery of neuropsychological tests was administered by the same examiner (KB), to maintain evaluator consistency, and via a HIPAA compliant video platform to minimize in-person contact time. Virtual neuropsychological evaluations have become increasingly common and have been found to provide a valid and reliable assessment of cognitive function in multiple patient populations (Iiboshi et al., 2020; Watt et al., 2021; Gallagher et al., 2023). Global cognition, attention, working memory, processing speed, executive functions, memory, language, and visual spatial skills were assessed using: Montreal Cognitive Assessment (MoCA) Blind; WAIS-IV Digit Span subtest; Stroop Color-Word Interference Test; Symbol Digit Modalities Test – oral version (SDMT); Controlled Oral Word Association Test (COWAT): FAS; Animal Fluency; Delis- Kaplan Executive Function Systems (DKEFS) Verbal Fluency: Category Switching subtest (fruit/furniture); Hopkins Verbal Learning Test-Revised (HVLT-R); Brief Visuospatial Memory Test-Revised (BVMT-R); Neuropsychological Assessment Battery (NAB) Naming Test; and Repeatable Battery for the Assessment of Neuropsychological Status (RBANS) Line Orientation subtest. The virtual assessment precluded inclusion of the Trail Making Test that shows impairments after COVID-19 (Miskowiak et al., 2021; Yesilkaya et al., 2021); therefore the DKEFS Category Switching subtest was used as an alternative test of cognitive flexibility. Validated Spanish versions were used for one participant whose primary language was Spanish. The following variables were analyzed based on literature documenting deficits in attention, working memory, processing speed, and memory in neuroPASC (Cecchetti et al., 2022; Kay et al., 2022; Vannorsdall et al., 2022; Díez-Cirarda et al., 2023): MoCA, SDMT, Stroop Color-Word, Stroop Interference, FAS, DKEFS Category Switching (Fruit/Furniture), Digit Span backward, Digit Span combined, HVLT-R total recall, HVLT-R delayed recall (DR), BVMT-R total recall, BVMT-R DR.

### Blood biomarker analyses

Details on how plasma SARS-CoV-2 nucleocapsid antibodies, hemoglobin A1c (HbA1C), high-sensitivity C-reactive protein (hsCRP), interleukin (IL)-1, IL-6, tumor necrosis factor-alpha (TNF-*α*), Aβ42/40 ratio, plasma phosphorylated tau-181 (pTau181), neurofilament light (NfL), and glial fibrillary acidic protein (GFAP) were quantified and how presence of APOE ε4 allele was determined are in Supplementary Methods. For data consistency, blood specimens collected at each site were shipped to the Advanced Research and Diagnostic Laboratory (ARDL, https://med.umn.edu/pathology/research/ardl) at the University of Minnesota and centrally analyzed using the methods detailed in Supplementary Methods. ARDL established a specimen collection protocol, prepared training materials for site personnel and shipped sample collection kits and bulk supplies to sites.

### Cluster analysis of neuroPASC symptoms

To identify clusters of participants based on self-reported symptom burden, the highest weekly frequency was used, with an ordinal scale: not present = 0, less than once per week = 1, 1–2 times per week = 3, 3–4 times per week = 4, 5–6 times per week = 5, every day = 6. The gap between 1 and 3 was intentionally widened to reflect the substantive difference between sporadic and weekly symptom occurrence (“less than once per week” vs. “1–2 times per week”). This non-linear encoding preserves ordinal rank while emphasizing clinically meaningful transitions for clustering analysis. As a sensitivity analysis, we repeated the clustering analysis using a linear encoding of symptom frequency (0–5) to confirm that the identified clusters were not driven by the deliberate introduction of a widened frequency gap. A heatmap of subject-level symptom frequency is provided in Supplementary Figure 1. Because seizures following COVID-19 infection were reported by only one participant, this symptom was excluded from the cluster analysis. We performed unsupervised K-means clustering on symptom frequency data after standardizing each symptom to z-scores across participants. Clustering was repeated using 1,000 random starts and 10 iterations per run using the “kmeansruns” function in the “fpc” package in R version 4.3.0 (R Foundation for Statistical Computing, Vienna, Austria). We used the “NbClust” R package (Charrad et al., 2014), evaluating 30 cluster validity indices including Silhouette, Calinski–Harabasz, Davies–Bouldin, Dunn, and others. The optimal number of clusters was determined by majority vote across all indices and across 2 to 10 clusters.

### Statistical analyses

Statistical analyses were performed in R. To evaluate group differences in neurocognitive assessment, quality-of-life scales, behavior history, BMI, blood pressure and blood biomarkers we used ANCOVA followed by pairwise tests for group comparisons, while adjusting for age and sex differences. Given the known association between BMI and hsCRP (Choi et al., 2013), we additionally included a group × BMI interaction term in the model when assessing group differences in hsCRP. We applied logarithmic transformation with a base of 10 to hsCRP measures and inverse transformations to IL-1 and IL-6 measures, when assessing group differences, due to high skewness in these measures. For clinical relevance, we present non-transformed values in tables. Cohen’s d effect sizes were computed for the pairwise comparisons of neurocognitive and quality-of-life measures using covariance-corrected residuals, where linear regression was used to adjust for effects of age and sex. For other continuous variables we used Kruskal-Wallis followed by Dunn’s adjusted pairwise comparisons. Group differences in categorical variables were assessed using Fisher’s exact test. We used the Benjamini-Hochberg false discovery rate (FDR, *p* < 0.05) to account for multiple testing. Relevant statistical tests are described as footnotes in Tables 1, 2 and Supplementary Tables.

**Table 2:** Behavioral history, quality-of-life and blood biomarkers of participants with neuroPASC and controls.

Variable	Control participants (*n* = 74)	Participants with NeuroPASC			*p* value neuroPASC vs control	*p* value three group comparison (Control, low-burden, high-burden neuroPASC)
		Total group (*n* = 102)	Low-burden neuroPASC (*n* = 75)	High-burden neuroPASC (*n* = 27)		
Behavior history						
Tobacco						
Current use, No. (%)	3 (4%)	5 (5%)	2 (3%)	3 (11%)	>0.99^a^	0.7^b^, 0.5^c^, 0.3^d^
Pack-years, median (IQR)	0 (0–0)	0 (0–0.1)	0 (0–0)	0 (0–2.3)	0.2^e^	0.1^f^, 0.7^g^, 0.1^h^, 0.1^i^
Alcohol						
Current use, No. (%)	56 (76%)	64 (63%)	51 (68%)	13 (48%)	0.1^a^	0.4^b^, **0.04**^**c**^, 0.2^d^
Past use, No. (%)	64 (86%)	89 (87%)	66 (88%)	23 (85%)	>0.99^a^	>0.99^b^, >0.99^c^, >0.99^d^
Age started drinking, median (IQR)	19 (18–21)	18 (16–21)	18 (17–21)	16 (14–18.5)	**0.04** ^j^	**<0.001**^k^, 0.2^l^, **<0.001**^m^, **0.001**^n^
Drink-years, median (IQR)	1.9 (0.6–7.6)	2.5 (1–14)	2.3 (0.8–8.8)	6.6 (1.6–28.9)	0.3^e^	**0.03**^f^, 0.8^g^, **0.02**^h^, **0.02**^i^
Cannabis						
Current use, No. (%)	11 (15%)	19 (19%)	13 (17%)	6 (22%)	0.7^a^	0.8^b^, 0.8^c^, 0.8^d^
Use-years, median (IQR)	0 (0–0.2)	0 (0–0.3)	0 (0–0.2)	0 (0–1.1)	0.3^e^	0.5^f^, 0.8^g^, 0.8^h^, 0.8^i^
Quality-of-life, median (IQR)						
MFIS	28 (22–35)	72 (52–85), *n* = 101	64 (45–83), *n* = 74	82 (69–92)	**<0.001** ^e^	**<0.001** ^f^ **, <0.001** ^g^ **, <0.001** ^h^ **, <0.001** ^**i**^
MFIS_cog	13 (10–18)	34 (25–41), *n* = 101	32 (23–40), *n* = 74	38 (31–44)	**<0.001** ^e^	**<0.001** ^f^ **, <0.001** ^g^ **, <0.001** ^h^ **, <0.001** ^**i**^
PSQI	4 (2–6)	8 (6–11), *n* = 100	8 (6–11), *n* = 73	10 (8–13)	**<0.001** ^e^	**<0.001**^f^**, <0.001**^g^**, <0.001**^h^, 0.06^i^
PHQ-8	2 (1–4)	8 (4–11), *n* = 96	7 (3–10), *n* = 71	11 (5–13), *n* = 25	**<0.001** ^e^	**<0.001** ^f^ **, <0.001** ^g^ **, <0.001** ^h^ **, 0.006** ^**i**^
GAD-7	1 (0–3)	4 (1–6), *n* = 101	4 (1–6)	5 (3–7)	**<0.001** ^e^	**<0.001**^f^**, <0.001**^g^**, <0.001**^h^, 0.09^i^
Blood biomarkers						
hsCRP (mg/L), median (IQR)	1 (0.5–2), *n* = 72	1.7 (0.7–2.8), *n* = 101	1.7 (0.6–2.7), *n* = 74	1.6 (0.8–3.4)	0.08^e^	0.3^f^, 0.3^g^, 0.3^h^, 0.7^i^
					**0.04** ^o^	0.4^p^, 0.5^q^, 0.5^r^, 0.7^s^
IL-1 (pg/mL), median (IQR)	0.4 (0.4–0.4), *n* = 72	0.4 (0.4–0.4), *n* = 101	0.4 (0.4–0.4), *n* = 74	0.4 (0.4–0.7)	0.7^e^	0.6^f^, 0.6^g^, 0.8^h^, 0.6^i^
IL-6 (pg/mL), median (IQR)	1.7 (1.5–2.4), *n* = 72	2 (1.5–3), *n* = 100	1.9 (1.5–2.9), *n* = 74	2.2 (1.5–3.2), *n* = 26	0.2^e^	0.8^f^, 0.9^g^, 0.9^h^, 0.9^i^
TNF-α (pg/mL), median (IQR)	7.5 (6.3–8.8), *n* = 72	7.3 (6.3–9.1), *n* = 101	7 (6.1–9.1), *n* = 74	7.8 (7–8.9)	0.3^e^	0.8^f^, 0.8^g^, 0.8^h^, 0.8^i^
Aβ42/40, median (IQR)	0.07 (0.06–0.07), *n* = 72	0.07 (0.06–0.07), *n* = 96	0.07 (0.06–0.07), *n* = 72	0.07 (0.06–0.07), *n* = 24	0.2^e^	0.4^f^, 0.5^g^, 0.7^h^, 0.7^i^
Presence of APOE ε4 allele, No. (%)	23 (35%), *n* = 66	23 (29%), *n* = 79	17 (28%), *n* = 61	6 (33%), *n* = 18	0.5^a^	>0.99^b^, >0.99^c^, >0.99^d^
pTau181 (pg/mL), median (IQR)	18.4 (14.9–23.6), *n* = 72	18.2 (14.2–24.5), *n* = 100	17.9 (14–22.8), *n* = 74	21.9 (16–27.7), *n* = 26	0.5^e^	0.9^f^, 0.8^g^, 0.8^h^, 0.8^i^
NfL (pg/mL), median (IQR)	6.9 (4.7–10.8), *n* = 72	7.6 (4.9–10.3), *n* = 101	6.5 (4.6–9.4), *n* = 74	8.2 (7.5–10.9)	0.7^e^	0.2^f^, 0.4^g^, 0.3^h^, 0.2^i^
GFAP (pg/mL), median (IQR)	58.3 (39.5–83.4), *n* = 72	56.5 (43.6–83.4), *n* = 101	55.1 (45.3–80.7), *n* = 74	60.6 (43.05–88.9)	0.5^e^	0.2^f^, 0.9^g^, 0.2^h^, 0.2^i^

Participants with neuroPASC were empirically grouped in two distinct clusters, low-burden and high-burden neuroPASC (Figure 2), based on the self-reported burden of neurological symptoms that were ongoing at the time of visit. Group comparisons were conducted using the Benjamini–Hochberg correction for multiple testing (false discovery rate, FDR < 0.05) across the following contrasts: controls vs. low-burden neuroPASC, controls vs. high-burden neuroPASC, and low-burden vs. high-burden neuroPASC. Sample sizes are indicated in cases with missing data. Tobacco pack-years was computed as (cigarettes per day/20) × years, assuming that a pack has 20 cigarettes. Alcohol drink-years was computed as (average number of drinks in one day × daily equivalent frequency) × years. Cannabis use-years was computed as daily equivalent frequency × years. MFIS: Modified Fatigue Impact Scale, MFIS _cog: MFIS cognitive subscale, PSQI: Pittsburgh Sleep Quality Index, PHQ-8: Patient Health Questionnaire-8, GAD-7: General Anxiety Disorder-7.

a Fisher’s exact test.

b Fisher’s exact test, control vs low-burden neuroPASC.

c Fisher’s exact test, control vs high-burden neuroPASC.

d Fisher’s exact test, low-burden vs high-burden neuroPASC.

e ANCOVA 1 degree of freedom F-test, adjusted for age and sex.

f ANCOVA 2 degree of freedom F-test, adjusted for age and sex.

g Pairwise comparison, control vs low-burden neuroPASC, adjusted for age and sex.

h Pairwise comparison, control vs high-burden neuroPASC, adjusted for age and sex.

i Pairwise comparison, low-burden vs high-burden neuroPASC, adjusted for age and sex.

j Mann–Whitney U test.

k Kruskal-Wallis rank sum test.

l Dunn adjusted pairwise comparison, control vs low-burden neuroPASC.

m Dunn adjusted pairwise comparison, control vs high-burden neuroPASC.

n Dunn adjusted pairwise comparison, low-burden vs high-burden neuroPASC.

o ANCOVA 1 degree of freedom F-test, adjusted for age and sex and group interaction with BMI.

p ANCOVA 2 degree of freedom F-test, adjusted for age, sex and group interaction with BMI.

q Pairwise comparison, control vs low-burden neuroPASC, adjusted for age, sex and group interaction with BMI.

r Pairwise comparison, control vs high-burden neuroPASC, adjusted for age, sex and group interaction with BMI.

s Pairwise comparison, low-burden vs high-burden neuroPASC, adjusted for age, sex and group interaction with BMI.Bold values were used to highlight significant *p*-values (*p* < 0.05).

We performed a multivariable logistic regression to compare clusters identified by our clustering procedure to each other on participant characteristics, including age, sex, education, BMI, Charlson Comorbidity Index (CCI), Framingham Risk Score (FRS), presence of pre-existing endocrine/metabolic and/or gastrointestinal/hepatobiliary conditions, COVID-19 vaccination prior to infection, hospitalization during acute infection, and alcohol use as covariates. Adjusted associations were expressed as odds ratios (ORs) with corresponding 95% confidence intervals (CIs).

## Results

### Characteristics of the neuroPASC cohort

To build a cohort of individuals without prior chronic neurological, active psychiatric or post-viral disease, a total of 2057 individuals were screened by filling out an online form or by physician referral. We contacted 434 individuals for a detailed phone screening, and enrolled 198 individuals who passed this second screening step. Of these 198 individuals, 22 were withdrawn due to scheduling conflicts or meeting exclusion criteria (Figure 1). A total of 102 participants with neuroPASC, 25% of whom were hospitalized during their COVID-19 illness, were assessed a median of 2 years after infection and compared to 74 control participants with no known SARS-CoV-2 infection (Table 1).

Most common neurological symptoms reported by the neuroPASC group were altered mental status (77%), headaches (70%), and muscle weakness (41%) (Figure 2A). Participants with neuroPASC reported fatigue (MFIS ≥ 38 in 86%), sleep disturbances (PSQI ≥ 5 in 94%), moderate-to-severe depression (PHQ-8 ≥ 6 in 63%) and moderate-to-severe anxiety (GAD-7 ≥ 10 in 13%), and had higher BMI and higher likelihood of being unable to work than controls (Table 1; Supplementary Table 1). Only 39% of the participants with neuroPASC were vaccinated prior to having COVID-19. The majority of participants with neuroPASC (93%) tested positive for the SARS-CoV-2 nucleocapsid antibody, demonstrating presence of nucleocapsid antibodies ~2 years post-infection. Notably, 51% of the control participants tested positive, demonstrating asymptomatic exposure to SARS-CoV-2. There were no significant differences in age, sex, years of education, vaccination status at study visit, behavioral history, blood pressure, CCI, FRS, and inflammatory (IL-1, IL-6 and TNF-*α*) and neurodegeneration (Aβ42/40, pTau181, NfL, GFAP and presence of APOE ε4 allele) blood biomarkers between the neuroPASC and control groups (Tables 1, 2). Concentration of hsCRP did not differ significantly between controls and individuals with neuroPASC after adjustment for age and sex alone (*p* = 0.08; Table 2). However, after additional adjustment for BMI and inclusion of a group × BMI interaction term, we found hsCRP to be significantly elevated in neuroPASC compared to controls (*p* = 0.04; Table 2). The BMI × group interaction itself did not reach statistical significance (*p* = 0.2). Additionally, both hsCRP and IL-6 levels were higher in hospitalized participants than both controls and non-hospitalized participants with neuroPASC (Supplementary Table 4). We also observed a significant interaction between hospitalization during acute COVID-19 and BMI on hsCRP levels, such that hsCRP increased more steeply with increasing BMI among hospitalized participants compared to non-hospitalized and control participants (*F* = 4.65, df = 2, *p* = 0.01).

### NeuroPASC clusters based on symptom burden

**Figure 3:**
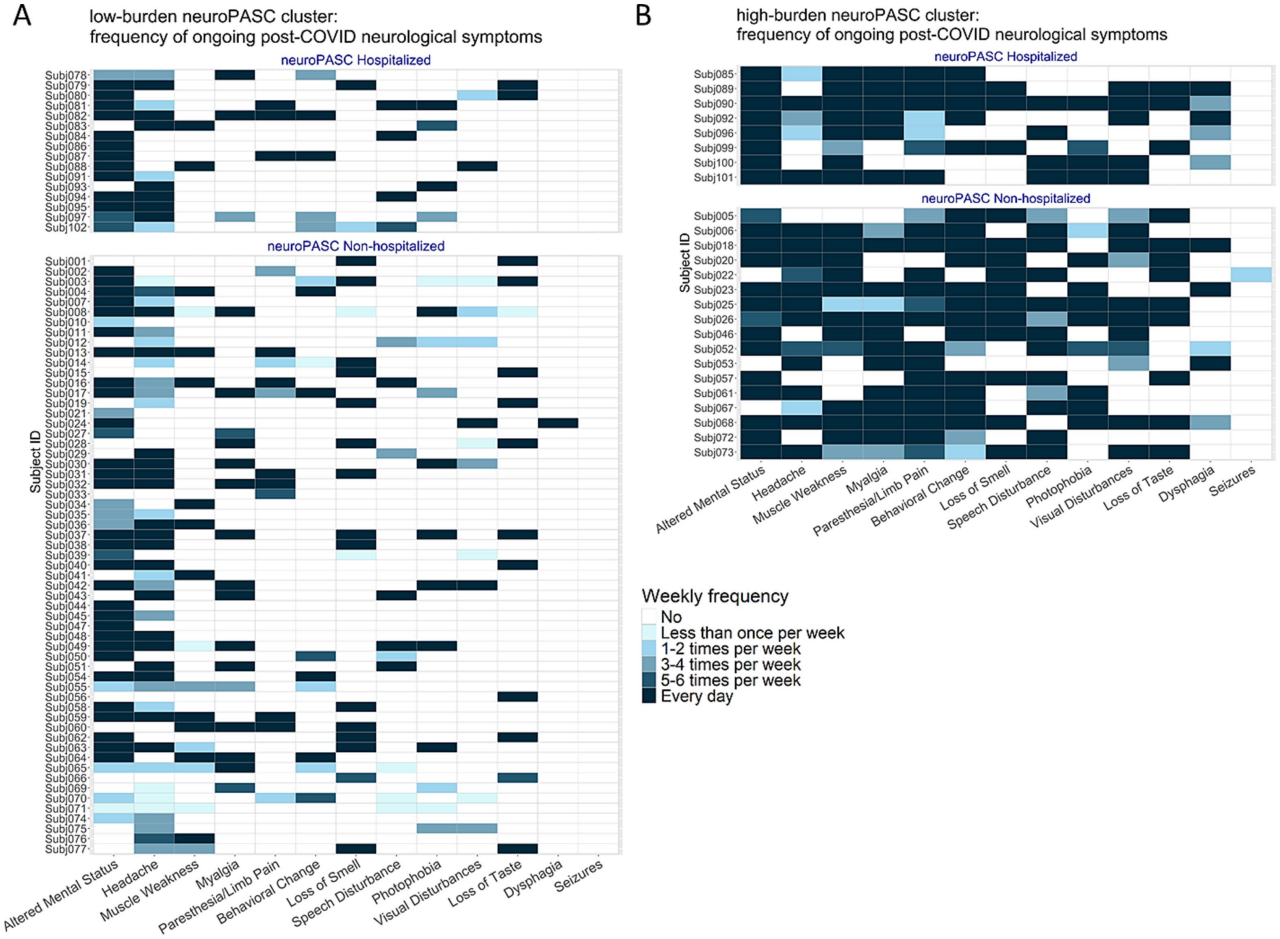
Frequency of ongoing post-COVID neurological symptoms among participants in the **(A)** low-burden and **(B)** high-burden neuroPASC clusters (see Figure 2). Subject-level heatmaps are color-coded by weekly frequency of each symptom when most severe. Only ongoing symptoms, those still present within the 14 days before the study visit, are included.

We investigated if there are clusters of individuals who experience differing levels of neuroPASC symptom burden. The optimal number of clusters based on self-reported symptom burden was two, supported by a majority of internal clustering validity indices, including the Calinski–Harabasz index and average silhouette width (Figure 2B; Supplementary Figure 2). The larger cluster was characterized by lower burden of neurological symptoms, with 1 to 8 self-reported symptoms and overall low weekly symptom frequency (termed “low-burden neuroPASC” from here on, Figure 3A). Altered mental status (73%) and headaches (68%) were the most common symptoms in this cluster (Figure 2C), and 21% of the participants in this cluster had been hospitalized during acute illness (Table 1). The second cluster was characterized by higher burden of neurological symptoms, with 5 to 12 self-reported symptoms and overall high weekly symptom frequency (termed “high-burden neuroPASC,” Figure 3B). Paresthesia/limb pain (85%), muscle weakness (85%) and altered mental status (85%) were the most common symptoms in this cluster (Figure 2D), and 33% of the participants in this cluster had been hospitalized. The hospitalization rate was not different between the clusters. Using a linear encoding of symptom frequency (0–5) produced similar clustering results, with only two participants shifting from the high-burden to the low-burden cluster. Both individuals reported a high overall symptom burden (6–7 symptoms), with the majority (4–5 symptoms) occurring more than five times per week, supporting their original classification as high-burden.

### Quality-of-life, neurological and neurocognitive assessments in neuroPASC clusters

**Figure 4:**
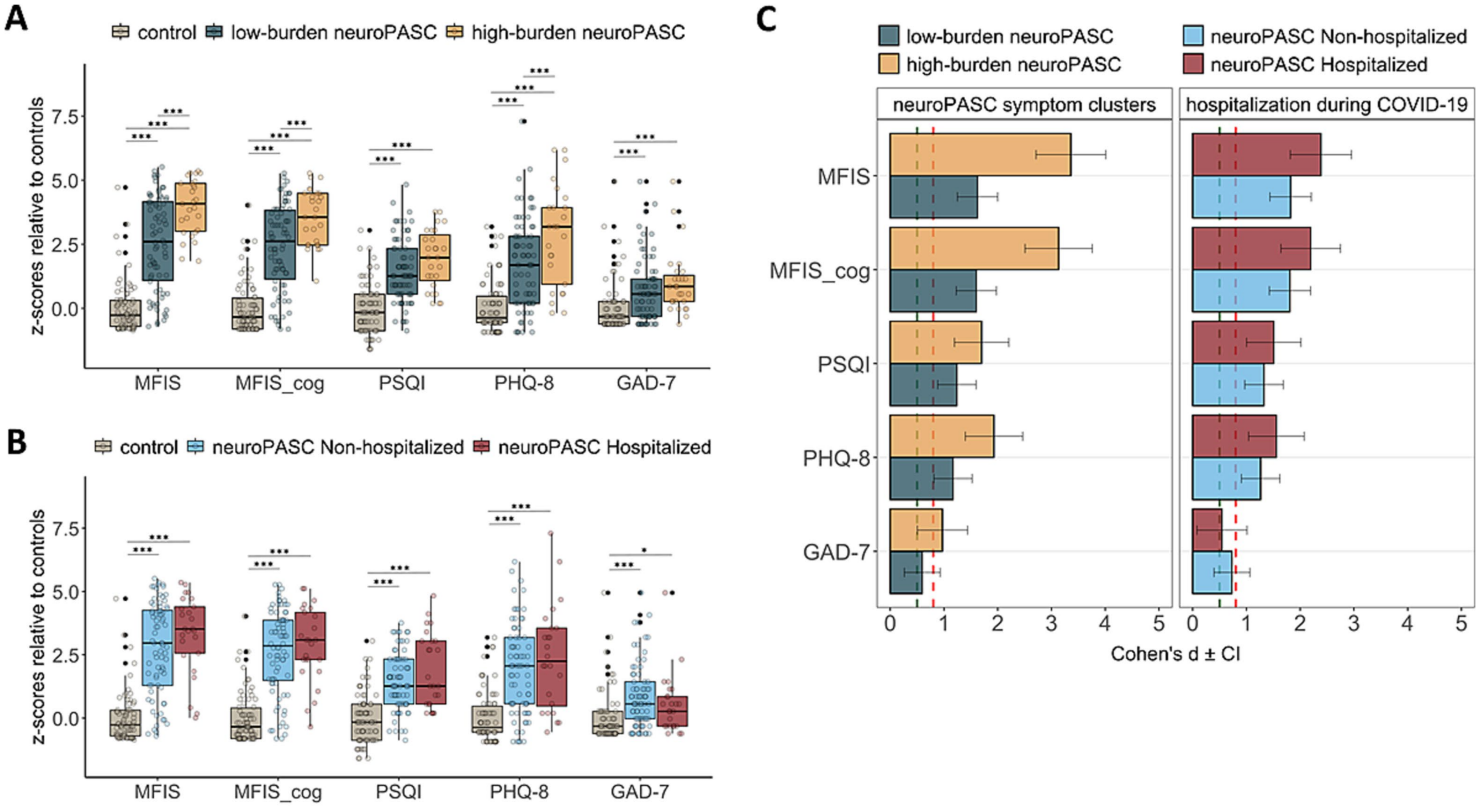
Quality-of-life questionnaires. Boxplots of quality-of-life scores in participants with neuroPASC, grouped by **(A)** post-COVID neurological symptom burden clusters (see Figure 2) and **(B)** hospitalization status during acute COVID-19. **(C)** Bar plots showing Cohen’s *d* effect sizes for comparisons between each neuroPASC group and controls, with participants grouped by post-COVID neurological symptom burden clusters (left) or by hospitalization status during acute COVID-19 (right). The thresholds for when an effect size is considered to be medium (0.5) or large (0.8), according to Cohen’s criteria, are represented by the vertical green and red dashed lines, respectively. Statistics are documented in Table 2. Group differences were assessed using ANCOVA followed by pairwise comparisons, while adjusting for age and sex. *p < 0.05, **p < 0.01, ***p < 0.001; *p*-values were adjusted for multiple testing using false discovery rate correction (3 x 5 = 15 comparisons). Black dots in **(A)** and **(B)** indicate outliers, which were retained in the analyses. MFIS: Modified Fatigue Impact Scale, MFIS_cog: MFIS cognitive subscale, PSQI: Pittsburgh Sleep Quality Index, PHQ-8: Patient Health Questionnaire-8, GAD-7: General Anxiety Disorder-7, CI: 95% confidence interval.

Next, we assessed if standardized quality-of-life, neurological and cognitive evaluations corroborated the self-reported symptom burden in the two clusters. Both neuroPASC clusters had higher fatigue, sleep disturbances, depression and anxiety scores than controls (Table 2), with higher scores overall in high-burden vs. low-burden neuroPASC (Figure 4A). These quality-of-life scores were not different between non-hospitalized and hospitalized cases (Figure 4B). Consistently, the high-burden neuroPASC cluster had the largest Cohen’s d effect sizes vs. controls across quality-of-life scales (Figure 4C). In addition, the high-burden neuroPASC cluster had a higher proportion of individuals who were unable to work than both controls and low-burden neuroPASC cluster (Supplementary Table 1).

The high-burden neuroPASC cluster also had a higher proportion of individuals exhibiting neurological signs compared to both controls and low-burden neuroPASC cluster (Supplementary Table 2), specifically reduced hearing, reduced muscle strength in the left upper extremity, reduced sensation to pain/temperature and light touch in the lower extremities, and abnormal gait (coordination/cerebellar function).

**Figure 5:**
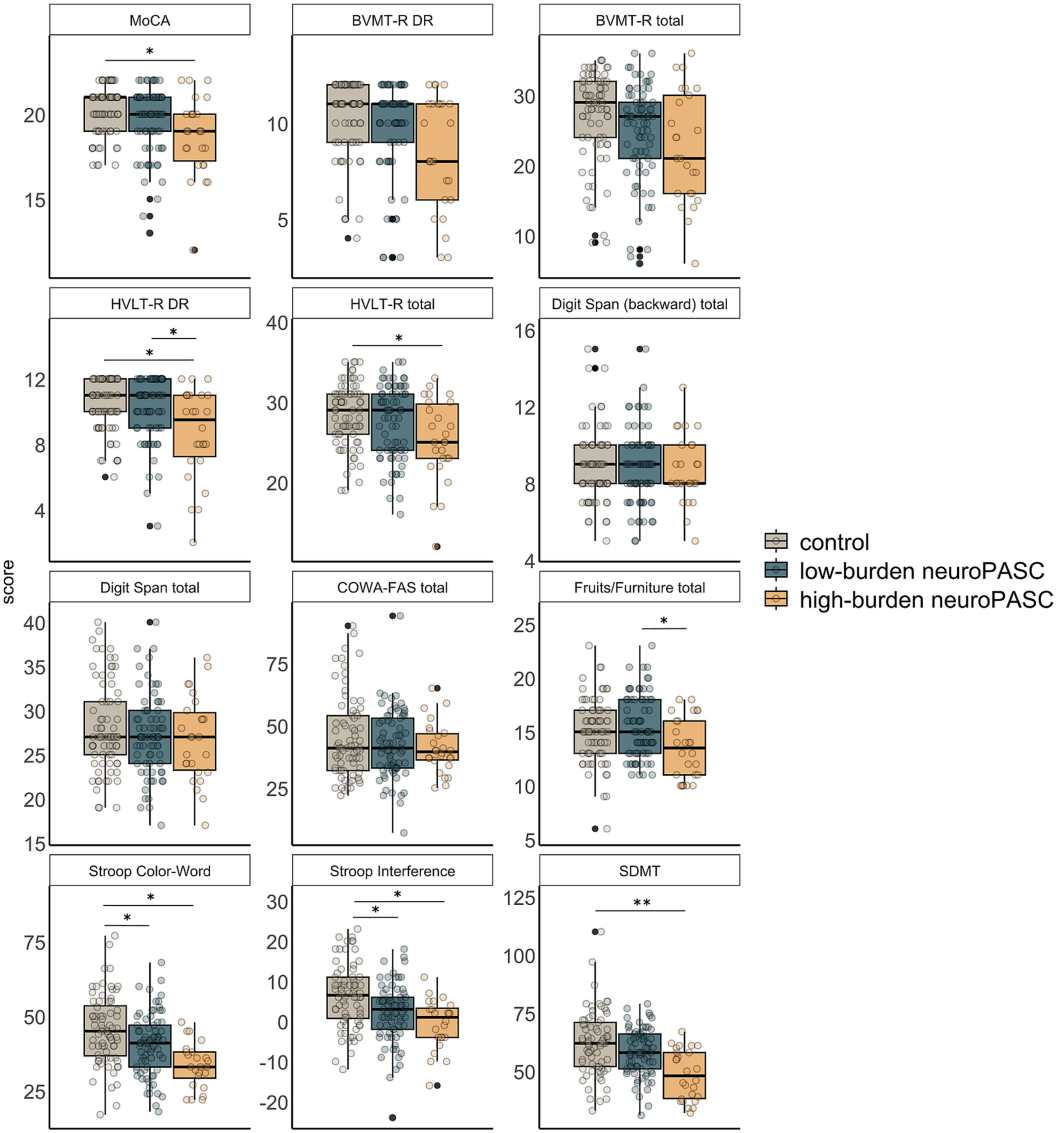
Boxplots of neurocognitive findings in participants with low-burden and high-burden neuroPASC (see Figure 2) and controls. Statistics are documented in Supplementary Table 5. Group differences were assessed using ANCOVA followed by pairwise comparisons, while adjusting for age and sex. *p < 0.05, **p < 0.01; *p*-values were adjusted for multiple testing using false discovery rate correction (3 × 12 = 36 comparisons). Black dots indicate outliers, which were retained in the analyses. MoCA: Montreal Cognitive Assessment, BVMT-R: Brief Visuospatial Memory Test Revised, HVLT-R: Hopkins Verbal Learning Test-Revised, DR: Delay recall, COWA-FAS: Controlled Oral Word Association Test, SDMT: Symbol Digit Modalities Test.

Participants in the high-burden neuroPASC cluster achieved lower scores on tests of cognitive screening (MoCA), verbal learning and memory (HVLT-R DR and HVLT-R total), response inhibition (Stroop Color-Word and Stroop Interference), and cognitive processing speed (SDMT) than controls, while those in the low-burden neuroPASC cluster only displayed impaired response inhibition. The high-burden neuroPASC cluster had lower HVLT-R DR and verbal fluency category switching (Fruit/Furniture) scores than the low-burden neuroPASC cluster (Figure 5; Supplementary Table 5). Similar to the quality-of-life outcomes, there was no difference in cognitive deficits between non-hospitalized and hospitalized participants (Supplementary Figure 3; Supplementary Table 6) and the high-burden neuroPASC cluster had the largest Cohen’s d effect sizes vs. controls, except for MoCA and Stroop interference (Supplementary Figure 4), where the hospitalized group displayed larger effect sizes.

Because the high-burden neuroPASC cluster was older than controls and the low-burden neuroPASC cluster, statistical analyses of the neurocognitive battery were repeated after removing participants younger than 30 years to mean-age-match groups and to assess if group differences in the cognitive battery were due to age differences (Supplementary Table 7). Differences between high-burden neuroPASC and controls did not change (Supplementary Table 8). Differences between high-burden and low-burden neuroPASC remained but did not reach statistical significance (HVLT_DR, Cohen’s d = −0.5, 95% CI, −0.98 to −0.02, pFDR = 0.07; Fruits/Furniture, Cohen’s d = −0.59, 95% CI, −1.07 to −0.1, pFDR = 0.06).

Therefore, the high-burden neuroPASC cluster that was identified based on self-reported symptoms consistently displayed more functional deficits based on validated quality-of-life, neurological and cognitive measures.

### Differences in cognitive function by SARS-CoV-2 nucleocapsid antibody status

Given that 38 (50%) of the control participants tested positive for the SARS-CoV-2 nucleocapsid antibody, indicating prior asymptomatic infection in half of the control group, we next examined whether nucleocapsid seropositivity was associated with differences in cognitive performance. To this end, we conducted a four-group analysis comparing both control subgroups (antibody-positive vs. antibody-negative) and the neuroPASC clusters, excluding the seven neuroPASC participants who tested antibody-negative. Antibody-negative controls were slightly older than antibody-positive controls (positive controls: 40 ± 17 years; negative controls: 48 ± 16 years; t = 2.2, *p* = 0.03), with no significant differences in sex or years of education between the groups.

After adjusting for age and sex, we observed lower scores on the COWA-FAS total (Cohen’s d = −0.64; 95% CI, −1.12 to −0.16; pFDR = 0.02) and Fruits/Furniture total (Cohen’s d = −0.69; 95% CI, −1.17 to −0.20; pFDR = 0.02) in control participants who tested positive for SARS-CoV-2 nucleocapsid antibodies compared with those who tested negative. When comparing the neuroPASC clusters to the control groups, differences were more pronounced between both neuroPASC clusters and the antibody-negative controls than between the clusters and antibody-positive controls. No significant differences were found when comparing the low-burden cluster to the antibody-positive control group (Supplementary Table 9).

### Factors associated with high-burden neuroPASC

Finally, we sought to identify clinical, behavioral and sociodemographic factors associated with high neurological symptom burden. The high-burden neuroPASC cluster did not differ from controls and low-burden neuroPASC with respect to sex, years of education, tobacco and cannabis use, blood pressure, HbA1C, and blood biomarkers of inflammation, neurodegeneration and amyloid burden. In addition, days since infection, hospitalization rate during acute illness, oxygen therapy during hospitalization, and BMI were not different between low-burden and high-burden neuroPASC clusters (Tables 1, 2). Similarly, while only 39% of the neuroPASC group had been vaccinated before COVID-19 vs. 96% of controls, the vaccination rate before COVID-19 was not different between low-burden vs. high-burden neuroPASC. The proportion of antibody-positivity also was not different between low-burden vs. high-burden neuroPASC (Table 1).

**Figure 6:**
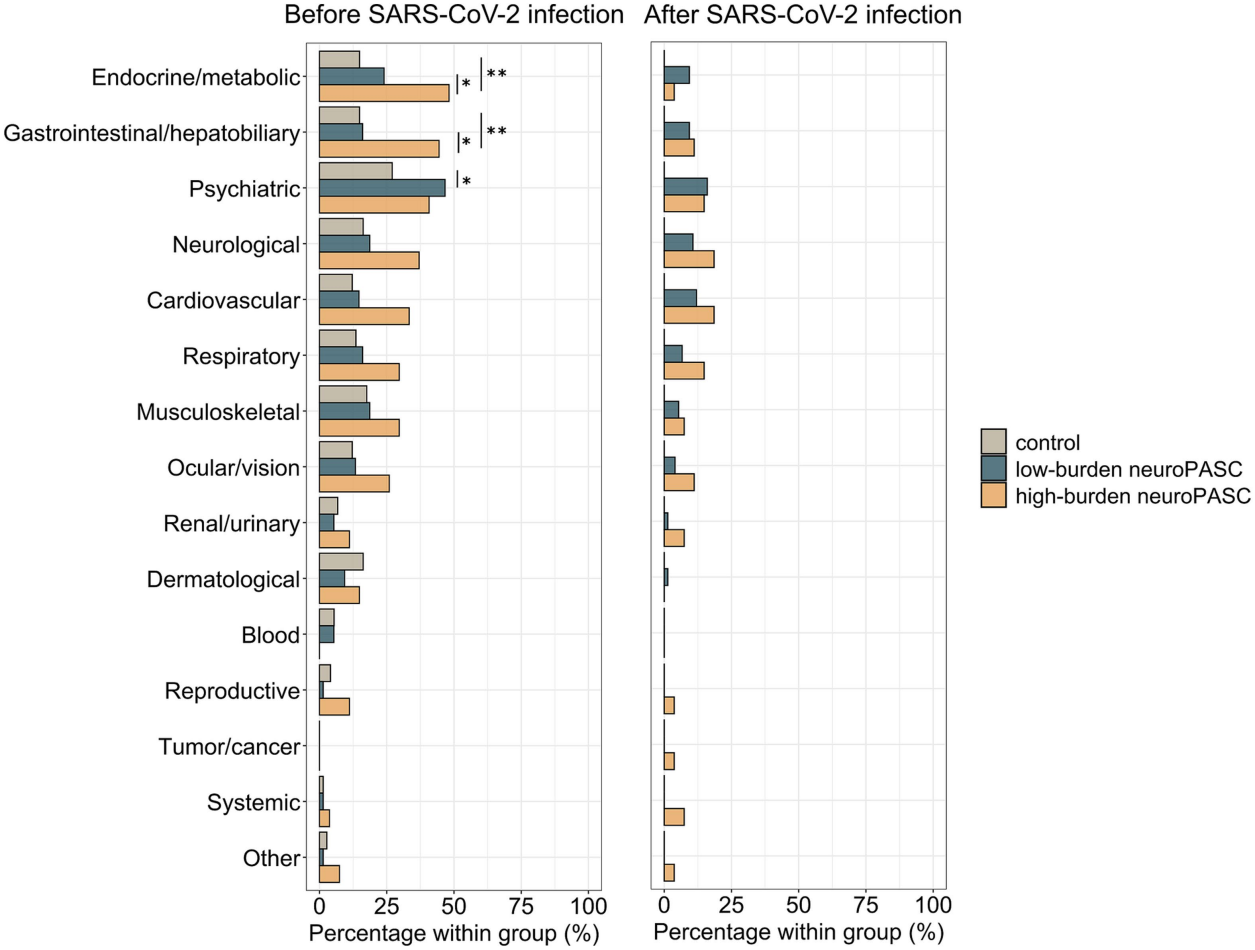
Bar plot illustrating the percentage of participants with ongoing medical conditions (present at the time of the study visit) with onset prior to (left) or following (right) SARS-CoV-2 infection, in the low-burden and high-burden neuroPASC clusters (see Figure 2) and in controls. Group differences were assessed using Fisher’s exact test per condition with false discovery rate adjusting for group testing (control vs. low-burden neuroPASC, control vs. high-burden neuroPASC, and low-burden vs. high-burden neuroPASC). *p < 0.05, **p < 0.01.

Participants with high-burden neuroPASC were significantly older than those with low-burden neuroPASC (Table 1). Specifically, all participants with high-burden neuroPASC were older than 30 years old and younger participants (20-to-30 years old) suffering from neuroPASC exhibited low symptom burden (Supplementary Figure 5). On the other hand, the entire age range of the high-burden neuroPASC group overlapped with that of low-burden neuroPASC and control groups, allowing good age-matching in the range above 30. The high-burden neuroPASC group had higher CCI, FRS, as well as greater cumulative alcohol use—reflected by higher alcohol drink-years and an earlier age of drinking onset—compared to both controls and low-burden neuroPASC. In a sensitivity analysis, we verified that after excluding individuals younger than 30 years, those with high-burden neuroPASC continued to show slightly higher CCI and FRS values compared to controls (controls above 30 years: CCI median = 1, FRS median = 4.6; high-burden neuroPASC: CCI median = 2, FRS median = 7.3); however, these differences were no longer statistically significant. Similarly, differences in drink-years remained, but reduced to trend levels, after excluding individuals younger than 30 years (controls above 30 years: drink-years median = 5.6; high-burden neuroPASC: drink-years median = 6.6, *p* = 0.05), likely due to the smaller sample size. The most common pre-existing conditions in high-burden neuroPASC were endocrine/metabolic (48%) and gastrointestinal/hepatobiliary (44%). These conditions were significantly more prevalent in the high-burden neuroPASC cluster (63% reporting one or both conditions) than in low-burden neuroPASC (34% reporting one or both conditions; Figure 6; Supplementary Table 3). The prevalence of these conditions did not differ between low-burden neuroPASC vs. controls (26% reporting one or both). To assess if the high prevalence of metabolic and endocrine conditions in the high-burden neuroPASC group was due to older age, we compared the group to the subset of controls ≥ 30 years. The prevalence in this control group (35% reporting one or both) remained significantly lower than that in the high-burden neuroPASC group.

The multivariable logistic regression model predicting high symptom burden (low-burden vs. high-burden), which included age, sex, education, BMI, CCI, FRS, pre-existing endocrine/metabolic and/or gastrointestinal/hepatobiliary conditions, COVID-19 vaccination prior to infection, hospitalization during acute infection, and cumulative alcohol use (drink-years) as covariates, showed that having pre-existing endocrine/metabolic and/or gastrointestinal/hepatobiliary conditions was independently associated with greater odds of high-burden neuroPASC (Table 3).

**Table 3:** Multivariable Logistic Regression predicting high symptom burden (1 = high-burden cluster; 0 = low-burden cluster).

Variable	OR (95% CI)	*p* value
Age	1.04 (0.98–1.12)	0.2
Sex	0.86 (0.19–3.5)	0.8
Hospitalized during COVID-19	1.04 (0.25–4.05)	0.9
Education	0.87 (0.66–1.12)	0.3
BMI	0.93 (0.85–1.01)	0.1
Pre-existing endocrine/metabolic and/or gastrointestinal/hepatobiliary conditions	3.47 (1.16–11.23)	**0.03**
Charlson comorbidity index	1.01 (0.52–2)	0.9
10-year framingham risk score	0.98 (0.88–1.1)	0.7
Vaccinated for COVID-19 before infection	1.01 (0.33–3.25)	0.9
Cumulative alcohol use (drink years)	1.01 (1–1.03)	0.1

Bold values were used to highlight significant *p*-values (*p* < 0.05).

## Discussion

We developed and deeply characterized a cohort of individuals who were neurologically healthy prior to having COVID-19, had mild–moderate COVID-19 and were still suffering from neuroPASC 2-years post-infection. The study design allowed the assessment of newly emerging neuroPASC symptoms without the confounds of chronic neurological or active psychiatric illness and mechanical ventilation during hospitalization for COVID-19. We identified two distinct neuroPASC clusters with different levels of self-reported disease burden, which were then characterized by validated quality-of-life, neurological and cognitive measures. Neurological symptom burden in individuals with neuroPASC was not associated with acute COVID-19 severity. Instead, higher neurological symptom burden was associated with older age, modestly elevated pre-existing comorbidity and cardiovascular risk factors, a longer alcohol use history, and pre-existing endocrine/metabolic and gastrointestinal/hepatobiliary conditions among individuals without prior history of chronic neurological disease.

Participants in the high-burden neuroPASC cluster experienced 5–12 ongoing symptoms most days and comprised a quarter of those who report neuroPASC symptoms in the COVID-BRAIN cohort. Notably, the percentage of hospitalized participants was similar in low-burden and high-burden neuroPASC clusters, therefore a need for hospital care during acute COVID-19 was not associated with high-burden neuroPASC in this cohort. This is in line with previous research showing similar cognitive impairments in hospitalized and non-hospitalized individuals after COVID-19 (Miskowiak et al., 2023). The proportion of individuals needing oxygen therapy during hospitalization was also similar in low-burden and high-burden neuroPASC clusters, therefore hypoxemia was not associated with high-burden neuroPASC. It is important to note that individuals who needed mechanical ventilation during hospitalization, i.e., the most severe acute cases, were excluded from the COVID-BRAIN cohort and are known to experience severe neurological sequelae (Mao et al., 2020; Irisson-Mora et al., 2022). Most long COVID studies to date have focused on hospitalized cohorts; our findings highlight the importance of assessing neurological post-COVID symptoms regardless of the severity of the acute infection.

Older age (Mansell et al., 2022) and comorbidity burden, particularly higher cardiovascular risk (Khatib et al., 2023; Tsampasian et al., 2024), were shown to increase the risk for both severe COVID-19 and long COVID. Our study indicates that these factors are also associated with high-burden neuroPASC. Our findings differ from a prior study reporting greater neurological symptom burden, cognitive deficits, and reduced quality-of-life among younger and middle-aged non-hospitalized individuals with neuroPASC compared with older (65 years and older) adults (Choudhury et al., 2025). In our cohort, older age among non-hospitalized participants was associated with lower cognitive performance and higher fatigue and depression scores (Supplementary Figures 7, 8). However, direct comparison between studies is limited by differences in age distribution – particularly the small number of participants over 65 in the COVID-BRAIN cohort – and by differing recruitment strategies, with the prior study recruiting from a specialized Neuro-COVID-19 clinic, whereas our participants were recruited primarily through community outreach.

Remarkably, CCI and FRS of the high-burden neuroPASC cluster in the current cohort were mostly in the low-to-intermediate risk range, indicating that relatively modest cardiovascular and comorbidity risk is observed in high-burden neuroPASC. However, this association was no longer significant after age-matching controls and low-burden to high-burden participants, suggesting that the effect may be partially age-dependent. We also observed a longer history of alcohol use—reflected by higher alcohol drink-years and an earlier age of drinking onset—among individuals with high-burden neuroPASC compared to those with low-burden neuroPASC and controls. Interestingly, the proportion of participants currently consuming alcohol was lower in the high-burden neuroPASC cluster than in the low-burden cluster, possibly reflecting behavioral changes following symptom onset. Chronic alcohol consumption negatively impacts the immune system, with heavy drinking (>5 drinks/day) presenting a higher risk of COVID-19 disease progression (Friske and Spanagel, 2023). However, only two COVID-BRAIN participants with high-burden neuroPASC reported heavy drinking, while others were moderate drinkers (<1–2 drinks/day), suggesting that chronic moderate alcohol consumption may be associated with high-burden neuroPASC. Tobacco use is indicated as a risk factor for long COVID (Trofor et al., 2024), but tobacco use was low in the COVID-BRAIN cohort, limiting the dynamic range to observe a potential association with high-burden neuroPASC. Importantly, most participants with high-burden neuroPASC reported pre-existing metabolic and endocrine (e.g., hyperlipidemia, hypothyroidism, diabetes) or gastrointestinal (e.g., irritable bowel syndrome, gastroesophageal reflux disease) conditions or both (Supplementary Table 3). Endocrine and metabolic diseases have been linked to COVID-19 severity (Puig-Domingo et al., 2021; Esmaeilzadeh et al., 2022) and endocrine and gastrointestinal diseases to long COVID risk (Bansal et al., 2022; Jacobs et al., 2023). In a multivariable analysis, pre-existing endocrine/metabolic and/or gastrointestinal/hepatobiliary conditions were independently associated with higher odds of high-burden neuroPASC compared to those with low-burden neuroPASC—after controlling for all other variables (age, sex, BMI, alcohol use, etc.). This finding is important in light of the increasing appreciation of contributions of cross-body interactions to disease pathogenesis, specifically the profound links between the cardiovascular, endocrine, immune and nervous systems, and warrants further investigation in larger cohorts.

Long COVID is a highly heterogeneous condition and several phenotypes have been identified using clustering methods (Reese et al., 2023; Thaweethai et al., 2023; Geng et al., 2024; Moniz et al., 2024; Slurink et al., 2024). These studies have focused on multi-system symptoms, where neurological symptoms often appear as a distinct cluster (Reese et al., 2023; Moniz et al., 2024). The current study provides further insights into the heterogeneity within neuroPASC. Our data indicate that post-COVID neurological complaints in those without chronic neurological disease primarily comprise mental and cognitive disturbances in ¾ of cases, while other neurological signs and symptoms, including musculoskeletal, visual, speech and pain are also reported in the remaining ¼ of cases. Importantly, ~20% of those with high-burden neuroPASC present with gait abnormalities and ~30% with sensory deficits in lower extremities (Supplementary Table 2). In addition, ~30% of participants with high-burden neuroPASC exhibited reduced hearing. Because diabetes and alcohol use are major risk factors for sensory neuropathy and age for reduced hearing, we confirmed that there was still a significant association between high-burden neuroPASC and these neurological signs, after adjusting for age, endocrine conditions and alcohol use. Notably, COVID-19 has been linked to hearing loss and vertigo (Kim et al., 2024) and hearing loss is a risk factor for dementia (Livingston et al., 2024), therefore more research is needed to investigate this association.

We evaluated memory, attention, and executive function domains in the neuropsychological battery. Cognitive deficits were previously reported in the months after infection for hospitalized (Negrini et al., 2021) and non-hospitalized (Graham et al., 2021) patients with long COVID. Participants with neuroPASC achieved lower scores on cognitive screening (MoCA), verbal learning and memory, processing speed, and response inhibition than controls in our study, similar to what was reported in previous studies (Crivelli et al., 2022; Perrottelli et al., 2022). Consistent with their self-reported symptom burden, the high-burden neuroPASC group performed worse on cognitive testing than the low-burden group, with a statistically significant deficit in cognitive flexibility (verbal fluency switching). Participants in the high-burden neuroPASC cluster also showed greater impairment on quality-of-life measures than participants in the low-burden neuroPASC cluster, when compared to controls. Although age was included as a covariate in all statistical models, the high-burden neuroPASC group was on average older, and all younger participants (20–30 years) with neuroPASC fell into the low-burden cluster. To address this imbalance, analyses were repeated after excluding participants younger than 30 years to achieve mean age-matched groups, yielding similar results. Scatterplots further showed that cognitive and quality-of-life deficits were present across age ranges (Supplementary Figures 9, 10). The neuroPASC group had a higher prevalence of pre-existing psychiatric conditions (e.g., depression, anxiety, and attention-deficit/hyperactivity disorder; Supplementary Table 3) than controls. However, psychiatric comorbidities were not more prevalent in the high-burden neuroPASC cluster than controls or the low-burden neuroPASC cluster. Therefore, differences in neurological symptom burden, quality-of-life, and cognitive outcomes between the high vs. low-burden neuroPASC groups are unlikely to be driven by psychiatric comorbidity or altered symptom perception. Consistently, adjusting quality-of-life and neurocognitive analyses for pre-existing psychiatric conditions did not alter the results. Together, these standardized cognitive and quality-of-life assessments support the symptom-based clustering and indicate that functional impairment aligns with participants’ reported experiences.

Neurocognitive testing was done remotely, which precluded administration of the Trail Making test. As an alternative measure of cognitive flexibility, we administered the DKEFS category Switching subtest, which does not require a motor component. Prior studies support the validity of virtual administration of neuropsychological tests that rely on processing of visual stimuli but lack a motor component. Notably, no significant differences have been reported between in-person and virtual administration of the MoCA (Gallagher et al., 2023), the oral version of the SDMT (Gallagher et al., 2023; Krynicki et al., 2023; Rogers et al., 2023) and the Judgment of Line Orientation Test (Gallagher et al., 2023). One study reported higher scores on BVMT-R when administered virtually compared with in-person (Rogers et al., 2023). Although comparisons of virtual versus in-person administration of the Stroop Color-Word test are lacking, the evaluation of an alternative version of this test – the DKEFS Color-Word Interference Test – revealed a faster performance in the virtual format compared to the in-person format on the color naming condition, with no differences on the word reading or response inhibition conditions of the test (Krynicki et al., 2023).

Low vaccination rates and high BMI were associated with the development of neuroPASC, but were not associated with high-burden neuroPASC. Similarly, hsCRP levels were modestly higher in the entire neuroPASC group compared with controls after adjustment for BMI, consistent with low-grade systemic inflammation. However, hsCRP did not differ significantly between the low-burden and high-burden neuroPASC clusters. Other inflammatory biomarkers assessed here, such as IL-6 and TNF-*α*, have been found to be elevated in neuroPASC (Comeau et al., 2023). These inflammatory biomarkers were not different from controls in the current cohort, possibly due to the long duration between the infection and study visit (~2 years). However, hsCRP and IL-6 were higher in hospitalized participants than both controls and non-hospitalized participants with neuroPASC (Supplementary Table 4), even after adjusting for BMI, suggesting that persistent long-term systemic inflammation is associated with the severity of the acute illness. Notably, correlations between inflammatory markers and COVID-19 severity were reported during the acute phase of COVID-19 (Barros-Aragão et al., 2024).

Almost all participants with neuroPASC (93%) tested positive for SARS-CoV-2 nucleocapsid antibodies ~2 years post-infection, which is consistent with a longer antibody positivity observed in people with Post-COVID condition compared to those without (Beale et al., 2025). Therefore, our finding may indicate a longer persistence of nucleocapsid antibodies in neuroPASC, which is noteworthy considering that persistence of virus or viral fragments is one of the potential pathogenic mechanisms of long COVID (Zuo et al., 2024). However, a high percentage of antibody positivity may also result from repeated exposure to the virus, which we cannot rule out based on the current data. Importantly, half of control participants also tested positive for nucleocapsid antibodies, revealing SARS-CoV-2 exposure without symptoms. Interestingly, control participants who tested positive for the SARS-CoV-2 nucleocapsid antibody exhibited lower verbal fluency scores (COWA-FAS and Fruits/Furniture) compared with antibody-negative controls. A prior study has reported that individuals with SARS-CoV-2 infection–related antibodies show poorer performance in language comprehension and temporal orientation, particularly among older adults (Serena et al., 2023). However, further research is needed to clarify the relationship between prior SARS-CoV-2 exposure and specific cognitive domains.

Several limitations should be considered when interpreting these findings. Although consensus guidelines now exist for the definition of long COVID, consensus guidelines for the assessment and reporting of neurological symptoms in long COVID are still lacking. In addition, our study was designed in 2020, at a time when the characterization of long COVID symptomatology, particularly neurological manifestations, was still in its early stages. Our neurological symptom checklist was adapted from the COVID-Neuro Network case record form, which was developed through expert consensus to standardize data collection from patients with neurological complications of COVID-19. The checklist was further refined to better capture symptoms emerging as characteristic of long COVID. While this approach ensured clinical relevance at the time of study design, the resulting checklist does not represent a formally validated instrument. This study is further limited by the lack of data from the same participants before the pandemic, not allowing a direct comparison between pre-COVID and post-COVID measures. The inclusion of a control group that was also enrolled during the pandemic was therefore critical and allowed us to rule out population-level effects of the pandemic on the quality-of-life and neurocognitive measures. Also, the COVID-BRAIN cohort was generally healthy, with no major neurological and active psychiatric conditions before the pandemic, partially mitigating the lack of pre-COVID assessments in the neuroPASC group. The exclusion of individuals with active psychiatric or chronic neurological conditions reduces the generalizability to the general population at risk for neuroPASC. This decision was made deliberately to minimize the risk that the observed effects reflect pre-existing neurobiology, as both psychiatric and neurological disorders can independently lead to alterations in cognitive performance, neurological function, and inflammatory and neurodegeneration biomarkers (Sæther et al., 2023). Given the marked heterogeneity in long COVID symptomatology and clinical presentation, this approach helped preserve interpretability and statistical power within the achievable sample size of the COVID-BRAIN project. By focusing on a well-defined cohort, we were able to investigate neuroPASC-related effects without confounding from major neurological or psychiatric disease. Future studies should extend these findings by including individuals with comorbid psychiatric or neurological conditions and by testing potential interaction effects between pre-existing mental and neurological illnesses and post-viral mechanisms. Another limitation was the lack of ethnic, racial and socioeconomic diversity in the cohort. Therefore, these findings cannot be generalized to sociodemographic risk and further work is needed in larger and more diverse neuroPASC cohorts. Finally, our cluster analysis identified two distinct clusters that should be interpreted as phenomenological groupings of symptom burden and frequency rather than discrete, biologically defined subtypes. Neurological symptoms were assessed using self-report, which may be influenced by recall or reporting bias and individual differences in symptom perception. However, such measures capture participants’ lived experiences and are widely used in long COVID research to assess fluctuating and subjective neurological symptoms. In addition, our analyses were based on symptom frequency rather than symptom intensity, which was not available in our dataset. Despite these limitations, we found that greater symptom frequency was associated with greater functional impairment, as evidenced by poorer quality-of-life, as well as worse neurological and cognitive outcomes.

In conclusion, we identified two presentations of persistent neuroPASC after COVID-19 in a previously neurologically healthy cohort with differing level of self-reported symptom burden. We characterized the two presentations by standardized measures of neurological and cognitive function. NeuroPASC symptom burden was not associated with acute COVID-19 severity, but was associated with older age, higher comorbidity burden, and greater cumulative alcohol use. The presence of pre-existing endocrine/metabolic or gastrointestinal/hepatobiliary conditions was the strongest independent associate, conferring a 3.5-fold increase in the odds of high-burden neuroPASC. These findings highlight modifiable behavioral and systemic medical risk factors, and motivate investigations into how endocrine, gastrointestinal, and neural pathways interact to shape neuroPASC severity.

## Data Availability

The datasets presented in this article are not readily available because deidentified participant data will be made available to researchers whose proposed used of the data has been approved. Requests to access the datasets should be directed to gulin@cmrr.umn.edu.

## Footnotes

1
https://covidbrainstudy.umn.edu/

## Group members of COVID-BRAIN Investigators

James M. Joers, Center for Magnetic Resonance Research, Department of Radiology, University of Minnesota, Minneapolis, MN, United States; Alfredo Lorente, Center for Magnetic Resonance Research, Department of Radiology, University of Minnesota, Minneapolis, MN, United States; Jeromy Thotland, Center for Magnetic Resonance Research, Department of Radiology, University of Minnesota, Minneapolis, MN, United States; Jaime Lavallee, Advanced Research and Diagnostic Laboratory, University of Minnesota, Minneapolis, MN, United States; Dinesh K. Deelchand, Center for Magnetic Resonance Research, Department of Radiology, University of Minnesota, Minneapolis, MN, United States; Young Woo Park, Center for Magnetic Resonance Research, Department of Radiology, University of Minnesota, Minneapolis, MN, United States; Xiufeng Li, Center for Magnetic Resonance Research, Department of Radiology, University of Minnesota, Minneapolis, MN, United States; Merve Atik, Department of Radiology, Mayo Clinic Rochester, Rochester, MN, United States; Matthew L. Senjem, Department of Radiology, Mayo Clinic Rochester, Rochester, MN, United States; Meher R. Juttukonda, Athinoula A. Martinos Center for Biomedical Imaging, Department of Radiology, Massachusetts General Hospital, Charlestown, MA, United States and Harvard Medical School, Boston, MA, United States; David H. Salat, Athinoula A. Martinos Center for Biomedical Imaging, Department of Radiology, Massachusetts General Hospital, Charlestown, MA, United States and Harvard Medical School, Boston, MA, United States; Janet C. Sherman, Harvard Medical School, Boston, MA, United States and Psychology Assessment Center, Department of Psychiatry, Massachusetts General Hospital, Harvard Medical School, Boston, MA, United States; G. Kyle Harrold, Harvard Medical School, Boston, MA, United States and Department of Neurology, Massachusetts General Hospital, Boston, MA, United States; Mehreen Nabi, Johns Hopkins University, Baltimore, MD, United States; Sana Rehman, Johns Hopkins University, Baltimore, MD, United States; Ipek Özdemir, Johns Hopkins University, Baltimore, MD, United States; Dillip Senapati, Johns Hopkins University, Baltimore, MD, United States; Peter B. Barker, Johns Hopkins University, Baltimore, MD, United States; Christof Karmonik, Johns Hopkins University, Baltimore, MD, United States and The Houston Methodist Research Institute, Houston, TX, United States; Syed A. Gillani, The Houston Methodist Research Institute, Houston, TX, United States; Valerie Flores, The Houston Methodist Research Institute, Houston, TX, United States; and Rachel Davis, The Houston Methodist Research Institute, Houston, TX, United States.
