# Supplementary material for: Factors associated with severe neurological sequelae of COVID-19: findings from the multicenter COVID-BRAIN imaging cohort
Source: Front Hum Neurosci. 2026 Mar 19;20:1754342. doi: 10.3389/fnhum.2026.1754342 (PMC13044115; doi:10.3389/fnhum.2026.1754342)
Supplement: Supplementary file 1 [file Data_Sheet_1.zip › Supplementary Material/Data Sheet_1.DOCX]

# SUPPLEMENTARY FORMS

# COVID-BRAIN NEUROLOGICAL SYMPTOMS

**NEUROLOGICAL SYMPTOMS DURING AND AFTER COVID-19:**

Please check if the patient experienced any of the following:

🞏 Headache 🞏 Altered Mental Status 🞏 Speech Disturbance

🞏 Behavioral change 🞏 Photophobia 🞏 Loss of Smell

🞏 Loss of Taste 🞏 Dysphagia 🞏 Myalgia

🞏 Muscle Weakness 🞏 Paresthesia/Limb Pain

🞏 Other (give details): _______________________________________

­­­­­­­­­­­­­­­­­­­­

Any seizure(s)? 🞏 Yes 🞏 No

If yes: 🞏 Focal 🞏 Generalized 🞏 Other (give details) ­­­­­­­­­­: ___________________

___________________

Any visual disturbance(s)? 🞏 Yes 🞏 No

If yes: 🞏 Visual field defect 🞏 Diplopia

🞏 Reduced acuity 🞏 Other (give details): ___________________

___________________

**COLLECT THE FOLLOWING FOR EACH SYMPTOM REPORTED:**

When was the symptom experienced?

🞏 During acute infection only 🞏 During and after acute infection 🞏 After acute infection only

If, after acute infection only, how many days or weeks after acute infection did the symptom start?

__________________________________

How many days per week was the symptom experienced by the participant while it was ongoing and at its most severe?

🞏 Every day 🞏 5-6 times per week 🞏 3-4 times per week

🞏 1-2 times per week 🞏 Less than once per week

Is the symptom ongoing (experienced within the past 14 days)? 🞏 Yes 🞏 No

If no, when did the participant last experience the symptom?

__________________________________ (mm/dd/yyyy)

Was the most severe time in the past 14 days? 🞏 Yes 🞏 No

If yes, how many days per week was the symptom experienced in the past 14 days?

🞏 Every day 🞏 5-6 times per week 🞏 3-4 times per week

🞏 1-2 times per week 🞏 Less than once per week

**COVID-BRAIN NEUROLOGICAL EXAM**

**MENINGEAL SIGNS** (neck stiffness, Brudzinski sign, Kernig sign): 🞏 Present 🞏 Absent

**MENTAL STATUS: Normal Abnormal Not Done Comment**

Level of consciousness 🞏 🞏 🞏 ___________________

Appearance/ facial/ motor expression 🞏 🞏 🞏 ___________________

Fund of knowledge 🞏 🞏 🞏 ___________________

Language function 🞏 🞏 🞏 ___________________

**CRANIAL NERVES: Normal Abnormal Not Done Comment**

Visual acuity (II) 🞏 🞏 🞏 ___________________

Pupils/ Fundi (II) 🞏 🞏 🞏 ___________________

Eye ductions (III, IV, VI) 🞏 🞏 🞏 ___________________

Eye saccades/ pursuit 🞏 🞏 🞏 ___________________

Jaw strength (V) 🞏 🞏 🞏 ___________________

Facial sensation (V) 🞏 🞏 🞏 ___________________

Facial strength (VII) 🞏 🞏 🞏 ___________________

Hearing (VIII) 🞏 🞏 🞏 ___________________

Swallowing pharynx, larynx (IX, X) 🞏 🞏 🞏 ___________________

SCM, trapezius (XI) 🞏 🞏 🞏 ___________________

Tongue (XII) 🞏 🞏 🞏 ___________________

**MOTOR SYSTEM: Normal Abnormal Not Done Comment**

Muscle bulk/ mass 🞏 🞏 🞏 ___________________

**Muscle Tone (rigid, spastic, or flaccid)**

Right upper extremity 🞏 🞏 🞏 ___________________

Left upper extremity 🞏 🞏 🞏 ___________________

Right lower extremity 🞏 🞏 🞏 ___________________

Left lower extremity 🞏 🞏 🞏 ___________________

**Disordered Movements (eg. Bradykinetic, tremor, chorea, myoclonus)**

Right upper extremity 🞏 🞏 🞏 ___________________

Left upper extremity 🞏 🞏 🞏 ___________________

Right lower extremity 🞏 🞏 🞏 ___________________

Left lower extremity 🞏 🞏 🞏 ___________________

**Muscle Strength**

Trunk 🞏 🞏 🞏 ___________________

Right upper extremity 🞏 🞏 🞏 ___________________

Left upper extremity 🞏 🞏 🞏 ___________________

Right lower extremity 🞏 🞏 🞏 ___________________

Left lower extremity 🞏 🞏 🞏 ___________________

**SENSATION: Normal Abnormal Not Done Comment**

**Upper**

Pain/ temperature 🞏 🞏 🞏 ___________________

Light touch 🞏 🞏 🞏 ___________________

Vibration 🞏 🞏 🞏 ___________________

Position 🞏 🞏 🞏 ___________________

**Lower Extremities**

Pain/ temperature 🞏 🞏 🞏 ___________________

Light touch 🞏 🞏 🞏 ___________________

Vibration 🞏 🞏 🞏 ___________________

Position 🞏 🞏 🞏 ___________________

**COORDINATION/**

**CEREBELLAR FUNCTION: Normal Abnormal Not Done Comment**

Gait 🞏 🞏 🞏 ___________________

Nystagmus 🞏 🞏 🞏 ___________________

Finger to nose 🞏 🞏 🞏 ___________________

Heel to shin 🞏 🞏 🞏 ___________________

**REFLEXES:**

| Not Done | **RIGHT** | 0 | 1+ | 2+ | 3+ | 4+ | **LEFT** | 0 | 1+ | 2+ | 3+ | 4+ | Not Done |
| --- | --- | --- | --- | --- | --- | --- | --- | --- | --- | --- | --- | --- | --- |
| ⭘ | Pectoral |  |  |  |  |  | Pectoral |  |  |  |  |  | ⭘ |
| ⭘ | Biceps |  |  |  |  |  | Biceps |  |  |  |  |  | ⭘ |
| ⭘ | Brachioradialis |  |  |  |  |  | Brachioradialis |  |  |  |  |  | ⭘ |
| ⭘ | Triceps |  |  |  |  |  | Triceps |  |  |  |  |  | ⭘ |
| ⭘ | Knee |  |  |  |  |  | Knee |  |  |  |  |  | ⭘ |
| ⭘ | Ankle |  |  |  |  |  | Ankle |  |  |  |  |  | ⭘ |
| ⭘ | Hoffman Sign | ⭘ Absent ⭘ Present ⭘ Equivocal | | | | | Hoffman Sign | ⭘ Absent ⭘ Present ⭘ Equivocal | | | | | ⭘ |
| ⭘ | Abdominals | ⭘ Absent ⭘ Present ⭘ Brisk | | | | | Abdominals | ⭘ Absent ⭘ Present ⭘ Brisk | | | | | ⭘ |
| ⭘ | Plantar | ⭘ Down ⭘ Up ⭘ Mute ⭘Equivocal | | | | | Plantar | ⭘ Down ⭘ Up ⭘ Mute ⭘Equivocal | | | | | ⭘ |
| ⭘ | Jaw Reflex | ⭘ Absent ⭘ Normal ⭘ Brisk | | | | | | | | | | | |
