# Supplementary material for: Factors associated with severe neurological sequelae of COVID-19: findings from the multicenter COVID-BRAIN imaging cohort
Source: Front Hum Neurosci. 2026 Mar 19;20:1754342. doi: 10.3389/fnhum.2026.1754342 (PMC13044115; doi:10.3389/fnhum.2026.1754342)
Supplement: Supplementary file 1 [file Data_Sheet_1.zip › Supplementary Material/Data Sheet_2.DOCX]

Supplementary Material

Index

[Supplementary Methods 2](#_Toc217891795)

[C-reactive Protein, High sensitivity (hsCRP) 2](#_Toc217891796)

[Interleukin 6 (IL-6) 2](#_Toc217891797)

[IL-1 beta 3](#_Toc217891798)

[TNF alpha 4](#_Toc217891799)

[HbA1C 4](#_Toc217891800)

[ApoE status 6](#_Toc217891801)

[Quanterix 4-Plex E (GFAP, AB40, AB42, NFL) 6](#_Toc217891802)

[Quanterix P-tau 181 7](#_Toc217891803)

[SARS-CoV-2 Antibody, Nucleocapsid 8](#_Toc217891804)

[Supplementary Tables 9](#_Toc217891805)

[Supplementary Figures 26](#_Toc217891806)

# Supplementary Methods

For data consistency, blood specimens collected at each site were shipped to the Advanced Research and Diagnostic Laboratory (ARDL, https://med.umn.edu/pathology/research/ardl) at the University of Minnesota and centrally analyzed using the methods detailed below.

## C-reactive Protein, High sensitivity (hsCRP)

hsCRP is measured in plasma using a latex-particle enhanced immunoturbidimetric assay kit (Roche Diagnostics, Indianapolis, IN 46250) and read on the Roche Cobas 8000 Chemistry analyzer (Roche Diagnostics). This is a two-reagent, immunoturbidimetric system. The specimen is first combined with a Tris buffer, then incubated. The second reagent (latex particles coated with mouse anti-human CRP antibodies) is then added and forms immune complexes. These complexes cause an increase in light scattering that is proportional to the CRP concentration. The light absorbance resulting from this light scatter is read against a stored CRP standard. Turbidity is measured at a primary wavelength of 546 nm (secondary wavelength 800 nm). The inter-assay CVs in our laboratory are 6.3% at a mean concentration of 3.07 mg/L and 5.0% at a mean concentration of 1.15 mg/L.

Sample type: EDTA plasma

Minimum volume needed: 100uL

Sample volume used: 6uL

Dead volume: 100uL

**References**

**1.** Cobas 8000 Analyzer Operator’s Manual. Roche Diagnostics Inc., 9115 Hague Road Indianapolis, IN 46250-0457. Version 5.3.

**2.** Roche PreciPath U Plus reagent package insert, Version 2020-03, V6.0. Roche Diagnostics Inc., 9115 Hague Road Indianapolis, IN 46250-0457.

**3.** Roche CRPHS reagent package insert. Version 2019-03, V12.0 English. Roche Diagnostics Inc., 9115 Hague Road Indianapolis, IN 46250-0457.

**4.** Roche Calibrator for Automated Systems, Proteins (C.F.A.S. Proteins) package insert. Version 2020-03, V 6.0 English. Roche Diagnostics Inc., 9115 Hague Road Indianapolis, IN 46250-0457.

## Interleukin 6 (IL-6)

IL-6 is measured in plasma on a Roche Cobas 8000 Analyzer (Roche Diagnostics Corporation) using a sandwich principle (Roche Diagnostics, Indianapolis, IN 46250). In the 1st incubation 18 µL of sample are incubated with a biotinylated monoclonal IL-6 specific antibody. After addition of a monoclonal IL-6 specific antibody labeled with a ruthenium complexa) and streptavidin-coated microparticles, the antibodies form a sandwich complex with the antigen of the sample. The reaction mixture is aspirated into the measuring cell where the microparticles are magnetically captured onto the surface of the electrode. Unbound substances are then removed. Application of a voltage to the electrode then induces chemiluminescent emission which is measured by a photomultiplier. Results are determined via a calibration curve which is instrument-specifically generated by 2 point calibration and a master curve provided via the reagent barcode or e barcode.

Sample type: EDTA plasma

Minimum volume needed: 150uL

Sample volume used: 18uL

Dead volume: 150uL

**References**

**1.** Cobas 8000 Analyzer Operator’s Manual. Roche Diagnostics Inc., 9115 Hague Road Indianapolis, IN 46250-0457.

**2.** Roche Elecsys IL-6 reagent package insert. Version 2020-08, V1.0. Roche Diagnostics Inc., 9115 Hague Road Indianapolis, IN 46250-0457.

**3.** Roche CalSet IL-6 package insert.. Version 2023-01, V1.0. Roche Diagnostics Inc., 9115 Hague Road Indianapolis, IN 46250-0457.

**4.** Roche PreciControl MultiMarker package insert. Version 2023-05, V6.0. Roche Diagnostics Inc., 9115 Hague Road Indianapolis, IN 46250-0457.

**5.** Roche CalCheck IL-6 package insert. Version 2021-03, V1.0. Roche Diagnostics Inc., 9115 Hague Road Indianapolis, IN 46250-0457.

## IL-1 beta

IL-1 Beta is measured using a Simple Plex assay on the Ella (BioTechne, Minneapolis, MN). In this method, sample runs through a microfluidic channel that binds IL-1 Beta. Next, Ella washes off unbound analyte and adds a detection reagent. Because each channel has three Glass Nano Reactors (GNRs) coated with a capture antibody, triplicate results for each sample are read. Concentrations are then generated from a factory-calibrated standard curve imbedded in each cartridge. Because each GNR is independent from the others, there is no cross-reactivity between channels and analytes.

Sample type: EDTA plasma

Minimum volume needed: 100 µL

Sample volume used: 25µL

Dead volume: 65µL

**References**

**1.** User Guide for ELLA, revision G 9/22/2016. Protein Simple, San Jose, CA

## TNF alpha

TNF alpha is measured using a Simple Plex assay on the Ella (BioTechne, Minneapolis, MN). In this method, sample runs through a microfluidic channel that binds TNF alpha. Next, Ella washes off unbound analyte and adds a detection reagent. Because each channel has three Glass Nano Reactors (GNRs) coated with a capture antibody, triplicate results for each sample are read. Concentrations are then generated from a factory-calibrated standard curve imbedded in each cartridge. Because each GNR is independent from the others, there is no cross-reactivity between channels and analytes.

Sample type: EDTA plasma

Minimum volume needed: 100 µL

Sample volume used: 25µL

Dead volume: 65µL

**References**

**1.** User Guide for ELLA, revision G 9/22/2016. Protein Simple, San Jose, CA.

## HbA1C

HbA1c is measured in EDTA whole blood on the Tosoh HPLC Glycohemoglobin Analyzer (Tosoh Medics, Inc., San Francisco CA 94080) using an automated high performance liquid chromatography method. This method is calibrated utilizing standard values derived by the National Glycohemoglobin Standardization Program (NGSP). Reference range is 4.3-6.0% with a laboratory CV range of 1.4-1.9%. Pooled, frozen human blood controls, from 1/01/21 to 6/30/21 (DN: 4.9%, CV 1.18%, DH: 10.5%, CV 0.70%) updated 7/13/21.

501rp middleware installed = 6/29/21

5.24 software update= 9/13/21

New Tosohs Installed= 8/02/21

Inter-assay CVs from the 209 and 309 instruments’ method validations (VA, 7/11/23)

209 instrument:

0.92% (HbA1c value of 4.9%)

0.68% (HbA1c value of 10.6%)

309 instrument:

0.92% (HbA1c value of 4.9%)

0.55% (HbA1c value of 10.6%)

Internal laboratory data and published studies (Selvin E, Coresh J, Jordahl J, Boland L, Steffes MW. Diabet Med. 2005;22:1726-30) confirm that whole blood frozen at -70C may be used for HbA1c measurements.

Sample type: EDTA-anticoagulated whole blood

Minimum volume needed: 50uL (1mL in a 3mL vacutainer preferred)

Sample volume used: 5uL

Dead volume: 1mL

**References**

**1.** G8 Variant Analysis Mode Operator’s Manual, TOSOH Bioscience, Inc., Inc. June 2011, version 2.5.

**2.** Coriello A, Giugliano D, Dello Russo P, Sgambato S, D’Onotrio F. Increased glycosylated hemoglobin A1 in opiate addicts. Evidence for hyperglycemic effect of morphine. Diabetologia 1962;22:379.

**3.** Goldstein DE, Little RR, Wiedmeyer HM, England JD, and McKenzie EM. Glycated hemoglobin: methodologies and clinical applications. Clin Chem 1986;32;B64-B70.

**4.** Nathan DM, Francis TB, Palmer JL. Effect of aspirin on determinations of glycosylated hemoglobin. Clin Chem 1983;29:466-9.

**5.** Fluckiger R, Harmon W, Meier W, Loo S, Gabbay KH. Hemoglobin carbamylation in uremia. N Eng J Med 1981;304:823-7.

**6.** Tze et al. Hemoglobin A1c – An Indicator of Diabetic Control. J of Pediatrics 1978, 93:1316.

**7.** American Diabetes Association, Standards of medical care for patients with diabetes mellitus (Position Statement). Diabetes Care. 1998;21 (Suppl. 1):S23-S31. G7 Automated HPLC Analyzer

**8.**Trivelli LA, Ranney HM, Lai H-T. Hemoglobin components in patients with diabetes mellitus. NEJM 1971; 284(7):353.

**9.** Bunn HF, Gabbay KH, Gallop PM. The glycosylation of hemoglobin: relevance to diabetes mellitus. Science 1678; 200:21-7.

**10.** Cerami A, Koenig RJ. Hemoglobin a1c as a model for the development of sequelae of diabetes mellitus. TIBS 1978; Apr:73.

## ApoE status

TaqMan Assays are used to detect specific variants in genomic DNA. TaqMan probes hybridize to the target DNA between two unlabeled PCR primers. Signal from the fluorescent dye on the 5´ end of a TaqMan probe is quenched by the NFQ on its 3´ end through fluorescence resonance energy transfer (FRET). During PCR, Taq polymerase extends the unlabeled primers using the template strand as a guide. When the polymerase reaches the TaqMan probe, it cleaves the molecule, separating the dye from the quencher. The qPCR instrument detects fluorescence from the unquenched FAM or VIC dye. Apolipoprotein E (Apo E) is a major cholesterol carrier and plays an important role in maintaining lipid homeostasis both in the periphery and brain. The Apo E gene is polymorphic at two nucleotides, (rs429358 and rs7412), resulting in three different alleles (E2, E3 and E4) and six Apo E genotypes E2/E2, E2/E3, E2/E4, E3/E3, E3/E4 and E4/E4. Differences among the three Apo E isoforms reside in the amino acid residues 112 and 158, where either cysteine or arginine is present, and the presence of either Cysteine or Arginine is determined by the DNA bases thymine (T) or Cytosine (C).

Sample type: acid-citrate-dextrose (ACD), NA-citrate, EDTA or heparin anticoagulant

Optimum volume: 5-10 mL fresh whole blood or 5 mL frozen packed cells

Minimum volume: 1 mL fresh whole blood or frozen packed cells

**References**

**1.** TaqMan® SNP Genotyping Assays User guide (MAN0009593). Revised 12/21. Applied Biosystems.

**2.** Wu L, Zhang X, et al. Human ApoE Isoforms Differentially Modulate Brain Glucose and Ketone Body Metabolism: Implications for Alzheimer's Disease Risk Reduction and Early Intervention. J Neurosci. 2018 Jul 25;38(30):6665-6681.

**3.** Zhong L, et al.. A rapid and cost-effective method for genotyping apolipoprotein E gene polymorphism. Mol Neurodegener. 2016 Jan 12;11:1

## Quanterix 4-Plex E (GFAP, AB40, AB42, NFL)

The Simoa® Neurology 4-Plex E assay is a digital immunoassay for the quantitative determination of Abeta 1-40, Abeta 1-42, GFAP, and NF-light in human CSF and plasma. The assay is run on the Simoa HD-X Analyzer (Billerica, MA).

Aβ40: Reference range: Plasma 88.3 – 142.7 pg/ml. The laboratory inter-assay CV is 4.8% at a mean concentration of 18.87 pg/mL, 2.4% at a mean concentration of 131.2 pg/ml, and 10.5% at a mean concentration of 37 pg/ml.

Aβ42: Plasma 4.9 – 8.9 pg/ml. The laboratory inter-assay CV is 5.1% at a mean concentration of 5.66 pg/mL, 3.7% at a mean concentration of 28.0 pg/ml, and 14.6% at a mean concentration of 2 pg/ml.

GFAP: Plasma 45.3 – 269.7 pg/ml. The laboratory inter-assay CV is 5.6% at a mean concentration of 230.79 pg/mL, 5.4% at a mean concentration of 4144.7 pg/ml, and 19.4% at a mean concentration of 60 pg/ml.

NF-Light: Plasma 6.6 – 30.0 pg/ml. The laboratory inter-assay CV is 9.8% at a mean concentration of 17.37 pg/mL, 8.5% at a mean concentration of 329.2 pg/ml, and 10% at a mean concentration of 10 pg/ml.

Sample type: EDTA plasma

Minimum volume needed: 500uL

Sample volume used: 400uL (100uL per measurement)

Dead Volume: n/a

**References**

**1.** Clinical & Laboratory Standards Institute. Document M29 Protection of Laboratory Workers from Occupationally Acquired Infections CLSI Web site https://clsi.org/standards/products/microbiology/documents/m29/. Accessed June 29, 2020

**2.** Simoa HD-X Analyzer® User Guide. USER-0033 11. SW3.1. Quanterix® Corporation 900 Middlesex Turnpike, Billerica, MA 01821

**3.** Simoa® Neurology 4-Plex E Advantage Kit Instructions. KI-0151 05. 06 Apr 2021. Quanterix® Corporation 900 Middlesex Turnpike, Billerica, MA 01821

## Quanterix P-tau 181

The Simoa® Human pTau-181 Advantage V2 assay is a digital immunoassay for the quantitative determination of pTau-181 in human CSF and plasma. The assay is run on the Simoa HD-X Analyzer (Billerica, MA). Reference Range: EDTA Plasma 8.7-25.0 pg/ml. The laboratory inter-assay CV is 4.5% at a mean concentration of 54.14 pg/mL, 6.2% at a mean concentration of 696.9 pg/ml, and 7.9% at a mean concentration of 16 pg/ml.

Sample type: EDTA plasma

Minimum volume needed: 200uL

Sample volume used: 100uL

Dead Volume: n/a

**References**

**1.** Clinical & Laboratory Standards Institute. Document M29 Protection of Laboratory Workers from Occupationally Acquired Infections CLSI Web site https://clsi.org/standards/products/microbiology/documents/m29/. Accessed June 29, 2020

**2.** Simoa HD-X Analyzer® User Guide. USER-0033 11. SW3.1. Quanterix® Corporation 900 Middlesex Turnpike, Billerica, MA 01821

**3.** Simoa® pTau-181 Advantage V2 Kit Kit Instructions for HD-1/HD-X

## SARS-CoV-2 Antibody, Nucleocapsid

Anti-SARS-CoV-2 is measured in plasma on a Roche Cobas 8000 Analyzer (Roche Diagnostics Corporation) using a sandwich immunoassay method (Roche Diagnostics, Indianapolis, IN 46250). In the first incubation 20 uL of sample, biotinylated SARS-CoV2-specific recombinant antigen, and SARS-CoV2-specific recombinant antigen labeled with a ruthenium complex form a sandwich complex. In the second incubation, after addition of streptavidin-coated microparticles, the complex becomes bound to the solid phase via interaction of biotin and streptavidin. The reaction mixture is aspirated into the measuring cell where the microparticles are magnetically captured onto the surface of the electrode. Unbound substances are then removed with ProCell M. Application of a voltage to the electrode then induces chemiluminescent emission which is measured by a photomultiplier. Results are determined by instrument software by comparing the electrochemiluminescence signal obtained from the reaction product of the sample with the signal of the cutoff value previously obtained by calibration. The laboratory inter-assay CV is 1.45% at a mean concentration of 0.105 IU/L and 1.77% at a mean concentration of 2.54 IU/L.

Sample type: EDTA plasma

Minimum volume used: 250 µL

Sample volume used: 12 µL

Dead volume: 200 µL

**References**

**1.** Cobas 8000 Analyzer Operator’s Manual. Roche Diagnostics Inc., 9115 Hague Road Indianapolis, IN 46250-0457.

**2.** Roche Cobas 8000 Anti-CoV-2 S reagent package insert. Version 2022-03, V3.0. Roche Diagnostics Inc., 9115 Hague Road Indianapolis, IN 46250-0457.

**3.** Roche ACOV2S CalSet package insert. Version 2024-05, V2.0. Roche Diagnostics Inc., 9115 Hague Road Indianapolis, IN 46250-0457.

**4.** Roche PreciControl ACOV2S package insert. Version 2024-09, V3.0 . Roche Diagnostics Inc., 9115 Hague Road Indianapolis, IN 46250-045

# Supplementary Tables

**Supplementary Table 1** – Social determinants of health among participants with neuroPASC and controls. Participants with neuroPASC were stratified into two clusters—low-burden and high-burden—based on the burden of self-reported post-COVID neurological symptoms persisting at the time of assessment. Group comparisons were conducted using the Benjamini–Hochberg correction for multiple testing (false discovery rate, FDR < 0.05) across the following contrasts: controls vs. low-burden neuroPASC, controls vs. high-burden neuroPASC, and low-burden vs. high-burden neuroPASC. Sample sizes are indicated where data were missing (e.g., employment status missing for one control participant).

| **Variable** | **Control participants (n=74)** | **Participants with neuroPASC** | | | ***P value* neuroPASC vs. control participants** | ***P value* three group comparison (Control, low-burden, high-burden neuroPASC)** |
| --- | --- | --- | --- | --- | --- | --- |
|  |  | **Total group (n=102)** | **Low-burden neuroPASC**  **(n=75)** | **High-burden neuroPASC (n=27)** |  |  |
| Race^a^, No. (%) | | | | | | |
| American Indian/ Alaska Native | 1 (1%) | 1 (1%) | 1 (1%) | 0 | **0.02**^b^ | 0.2^c^, 0.5^d^, 0.9^e^ |
| Asian | 7 (9%) | 1 (1%) | 1 (1%) | 0 |  |  |
| Black | 2 (3%) | 2 (2%) | 2 (3%) | 0 |  |  |
| White | 64 (86%) | 95 (93%) | 68 (91%) | 27 (100%) |  |  |
| Unknown | 0 | 3 (3%) | 3 (4%) | 0 |  |  |
| Education, No. (%) | | | | | | |
| 12th grade, no diploma | 0 | 2 (2%) | 1 (1%) | 1 (4%) | 0.3^b^ | 0.6^c^, 0.1^d^, 0.6^e^ |
| High school graduate | 1 (1%) | 5 (5%) | 4 (5%) | 1 (4%) |  |  |
| GED or equivalent | 1 (1%) | 0 | 0 | 0 |  |  |
| Some college, no degree | 8 (11%) | 8 (8%) | 7 (9%) | 1 (4%) |  |  |
| Associate degree | 6 (8%) | 18 (18%) | 10 (13%) | 8 (30%) |  |  |
| Bachelor’s degree | 36 (49%) | 38 (37%) | 30 (40%) | 8 (30%) |  |  |
| Master’s degree | 13 (18%) | 22 (22%) | 15 (20%) | 7 (26%) |  |  |
| Professional school degree | 4 (5%) | 5 (5%) | 5 (7%) | 0 |  |  |
| Doctoral degree | 5 (7%) | 4 (4%) | 3 (4%) | 1 (4%) |  |  |
| Current employment status, No. (%) | | | | | | |
| Working | 55 (75%), n=73 | 56 (55%) | 48 (64%) | 8 (30%) | **<0.001**^b^ | **0.01**^c^, **<0.001**^d^, **0.007**^e^ |
| Unable to work | 1 (1%), n=73 | 23 (23%) | 11 (15%) | 12 (44%) |  |  |
| Keeping house | 0, n=73 | 1 (1%) | 0 | 1 (4%) |  |  |
| Retired | 11 (15%), n=73 | 12 (12%) | 9 (12%) | 3 (11%) |  |  |
| Student | 4 (5%), n=73 | 1 (1%) | 1 (1%) | 0 |  |  |
| Unemployed | 2 (3%), n=73 | 5 (5%) | 4 (5%) | 1 (4%) |  |  |
| Other | 0 | 4 (4%) | 2 (3%) | 2 (7%) |  |  |
| Annual family income, No. (%) | | | | | | |
| Over $100,000 | 27 (37%) | 45 (44%) | 33 (43%) | 12 (44%) | 0.2^b^ | 0.8^c^, 0.8^d^, 0.8^e^ |
| $60,000 - $99,999 | 19 (25%) | 28 (28%) | 18 (24%) | 10 (37%) |  |  |
| $40,000 - $59,999 | 5 (7%) | 14 (14%) | 11 (15%) | 3 (11%) |  |  |
| $20,000 - $39,999 | 7 (9%) | 6 (6%) | 4 (5%) | 2 (7%) |  |  |
| $10,000 - $19,999 | 5 (7%) | 3 (3%) | 3 (4%) | 0 |  |  |
| Less than $10,000 | 4 (5%) | 1 (1%) | 1 (1%) | 0 |  |  |
| Prefer not to answer | 4 (5%) | 4 (4%) | 4 (5%) | 0 |  |  |
| I don’t know | 3 (4%) | 1 (1%) | 1 (1%) | 0 |  |  |
| Covered by health insurance, No. (%) | 72 (97%) | 99 (97%) | 72 (96%) | 27 (100%) | >0.99^b^ | >0.99^c^, >0.99^d^, >0.99^e^ |

a Race was assessed through self-report.

b Fisher’s exact test.

c Fisher’s exact test, control vs low-burden neuroPASC.

d Fisher’s exact test, control vs high-burden neuroPASC.

e Fisher’s exact test, low-burden vs high-burden neuroPASC.

**Supplementary Table 2** – Neurological signs among participants with neuroPASC and controls. Participants with neuroPASC were stratified into two clusters—low-burden and high-burden—based on the burden of self-reported post-COVID neurological symptoms persisting at the time of assessment. Group comparisons were conducted using the Benjamini–Hochberg correction for multiple testing (false discovery rate, FDR < 0.05) across the following contrasts: controls vs. low-burden neuroPASC, controls vs. high-burden neuroPASC, and low-burden vs. high-burden neuroPASC. Sample sizes are indicated where data were missing.

| **Variable** | **Control participants (n=74)** | **Participants with neuroPASC** | | | ***P value* neuroPASC vs. control participants** | ***P value* three group comparison (Control, low-burden, high-burden neuroPASC)** |
| --- | --- | --- | --- | --- | --- | --- |
|  |  | **Total group (n=102)** | **Low-burden neuroPASC (n=75)** | **High-burden neuroPASC (n=27)** |  |  |
| Meningeal signs, abnormal, No. (%) | 0 | 0 | 0 | 0 | >0.99^a^ | >0.99^b^, >0.99^c^, >0.99^d^ |
| Mental status, abnormal, No. (%) | | | | | | |
| Level of consciousness | 1 (1%) | 1 (1%), n=101 | 0, n=74 | 1 (4%) | >0.99^a^ | >0.99^b^, 0.7^c^, 0.7^d^ |
| Appearance/facial/motor expression | 1 (1%) | 1 (1%), n=101 | 0, n=74 | 1 (4%) | >0.99^a^ | >0.99^b^, 0.7^c^, 0.7^d^ |
| Fund of knowledge | 1 (1%) | 0, n=101 | 0, n=74 | 0 | >0.99^a^ | >0.99^b^, >0.99^c^, >0.99^d^ |
| Language function | 1 (1%) | 2 (2%), n=101 | 2 (3%), n=74 | 0 | >0.99^a^ | >0.99^b^, >0.99^c^, >0.99^d^ |
| Cranial nerves, abnormal, No. (%) | | | | | | |
| Visual acuity (II) | 5 (7%), n=68 | 7 (8%), n=90 | 5 (7%), n=69 | 2 (10%), n=21 | >0.99^a^ | >0.99^b^, >0.99^c^, >0.99^d^ |
| Pupils/ Fundi (II) | 0, n=65 | 4 (4%), n=92 | 2 (3%), n=69 | 2 (9%), n=23 | 0.1^a^ | 0.5^b^, 0.2^c^, 0.4^d^ |
| Eye ductions (III, IV, VI) | 2 (3%) | 2 (2%), n=99 | 1 (1%), n=72 | 1 (4%) | >0.99^a^ | >0.99^b^, >0.99^c^, >0.99^d^ |
| Eye saccades/ pursuit | 2 (3%) | 3 (3%), n=101 | 1 (1%), n=74 | 2 (7%) | >0.99^a^ | >0.99^b^, 0.4^c^, 0.4^d^ |
| Jaw strength (V) | 0, n=68 | 0, n=93 | 0, n=68 | 0, n=25 | >0.99^a^ | >0.99^b^, >0.99^c^, >0.99^d^ |
| Facial sensation (V) | 1 (1%), n=69 | 3 (3%), n=97 | 1 (1%), n=72 | 2 (8%), n=25 | 0.6^a^ | >0.99^b^, 0.3^c^, 0.3^d^ |
| Facial strength (VII) | 1 (1%) | 2 (2%), n=101 | 1 (1%), n=74 | 1 (4%) | >0.99^a^ | >0.99^b^, 0.7^c^, 0.7^d^ |
| Hearing (VIII) | 5 (8%), n=66 | 10 (11%), n=91 | 3 (5%), n=67 | 7 (29%), n=24 | 0.6^a^ | 0.5^b^, **0.02**^c^, **0.009**^d^ |
| Swallowing pharynx, larynx (IX, X) | 0 | 1 (1%), n=100 | 0, n=74 | 1 (4%), n=26 | >0.99^a^ | >0.99^b^, 0.4^c^, 0.4^d^ |
| SCM, trapezius (XI) | 0, n=73 | 0, n=99 | 0, n=72 | 0 | >0.99^a^ | >0.99^b^, >0.99^c^, >0.99^d^ |
| Tongue (XII) | 0 | 0, n=100 | 0, n=73 | 0, n=27 | >0.99^a^ | >0.99^b^, >0.99^c^, >0.99^d^ |
| Motor system, abnormal, No. (%) | | | | | | |
| Muscle bulk/mass | 0, n=71 | 1 (1%), n=98 | 0, n=73 | 1 (4%), n=25 | >0.99^a^ | >0.99^b^, 0.4^c^, 0.4^d^ |
| Muscle Tone (rigid, spastic, or flaccid) | | | | | | |
| Right upper extremity | 0, n=67 | 1 (1%), n=95 | 0, n=70 | 1 (4%), n=25 | >0.99^a^ | >0.99^b^, 0.4^c^, 0.4^d^ |
| Left upper extremity | 0, n=67 | 1 (1%), n=95 | 0, n=70 | 1 (4%), n=25 | >0.99^a^ | >0.99^b^, 0.4^c^, 0.4^d^ |
| Right lower extremity | 0, n=67 | 1 (1%), n=95 | 0, n=70 | 1 (4%), n=25 | >0.99^a^ | >0.99^b^, 0.4^c^, 0.4^d^ |
| Left lower extremity | 0, n=67 | 0, n=95 | 0, n=70 | 0, n=25 | >0.99^a^ | >0.99^b^, >0.99^c^, >0.99^d^ |
| Disordered Movements (Bradykinetic, tremor, chorea, myoclonus) | | | | | | |
| Right upper extremity | 0 | 3 (3%), n=101 | 1 (1%), n=74 | 2 (7%) | 0.3^a^ | 0.5^b^, 0.2^c^, 0.3^d^ |
| Left upper extremity | 0 | 3 (3%), n=101 | 2 (3%), n=74 | 1 (4%) | 0.3^a^ | 0.7^b^, 0.7^c^, >0.99^d^ |
| Right lower extremity | 0 | 0, n=101 | 0, n=74 | 0 | >0.99^a^ | >0.99^b^, >0.99^c^, >0.99^d^ |
| Left lower extremity | 0 | 1 (1%), n=101 | 0, n=74 | 1 (4%) | >0.99^a^ | >0.99^b^, 0.4^c^, 0.4^d^ |
| Muscle strength | | | | | | |
| Trunk | 0, n=71 | 1 (1%), n=100 | 0, n=73 | 1 (4%) | >0.99^a^ | >0.99^b^, 0.4^c^, 0.4^d^ |
| Right upper extremity | 0 | 2 (2%), n=101 | 0, n=74 | 2 (7%) | 0.5^a^ | >0.99^b^, 0.1^c^, 0.1^d^ |
| Left upper extremity | 0 | 5 (5%), n=101 | 1 (1%), n=74 | 4 (14%) | 0.07^a^ | 0.5^b^, **0.01**^c^, **0.03**^d^ |
| Right lower extremity | 0 | 0, n=101 | 0, n=74 | 0 | >0.99^a^ | >0.99^b^, >0.99^c^, >0.99^d^ |
| Left lower extremity | 0 | 0, n=101 | 0, n=74 | 0 | >0.99^a^ | >0.99^b^, >0.99^c^, >0.99^d^ |
| Sensation, abnormal, No. (%) | | | | | | |
| Upper Extremities | | | | | | |
| Pain/ temperature | 0, n=66 | 3 (3%), n=95 | 2 (3%), n=70 | 1 (4%), n=25 | 0.3^a^ | 0.7^b^, 0.7^c^, >0.99^d^ |
| Light touch | 0, n=67 | 1 (1%), n=97 | 1 (1%), n=72 | 0, n=25 | >0.99^a^ | >0.99^b^, >0.99^c^, >0.99^d^ |
| Vibration | 0, n=64 | 1 (1%), n=94 | 1 (1%), n=69 | 0, n=25 | >0.99^a^ | >0.99^b^, >0.99^c^, >0.99^d^ |
| Position | 0, n=61 | 0, n=91 | 0, n=68 | 0, n=23 | >0.99^a^ | >0.99^b^, >0.99^c^, >0.99^d^ |
| Lower Extremities | | | | | | |
| Pain/ temperature | 1 (2%), n=66 | 13 (14%), n=94 | 6 (9%), n=69 | 7 (28%), n=25 | **0.008**^a^ | 0.1^b^, **0.001**^c^, 0.055^d^ |
| Light touch | 0, n=67 | 7 (7%), n=96 | 2 (3%), n=71 | 5 (20%), n=25 | **0.04**^a^ | 0.5^b^, **0.003**^c^, **0.02**^d^ |
| Vibration | 5 (8%), n=64 | 16 (17%), n=93 | 9 (13%), n=68 | 7 (28%), n=25 | 0.1^a^ | 0.4^b^, 0.1^c^, 0.1^d^ |
| Position | 0, n=61 | 4 (4%), n=91 | 4 (6%), n=68 | 0, n=23 | 0.1^a^ | 0.4^b^, >0.99^c^, 0.9^d^ |
| Coordination/ cerebellar function, abnormal, No (%) | | | | | | |
| Gait | 0 | 10 (10%), n=101 | 4 (5%), n=74 | 6 (22%) | **0.005**^a^ | 0.1^b^, **0.0007**^c^, **0.03**^d^ |
| Nystagmus | 0 | 2 (2%), n=100 | 1 (1%), n=73 | 1 (4%) | 0.5^a^ | 0.5^b^, 0.5^c^, 0.5^d^ |
| Finger to nose | 0 | 2 (2%), n=99 | 1 (1%), n=72 | 1 (4%) | 0.5^a^ | 0.5^b^, 0.5^c^, 0.5^d^ |
| Heel to shin | 0, n=71 | 1 (1%), n=96 | 0, n=69 | 1 (4%) | >0.99^a^ | >0.99^b^, 0.4^c^, 0.4^d^ |

a Fisher’s exact test.

b Fisher’s exact test, control vs low-burden neuroPASC.

c Fisher’s exact test, control vs high-burden neuroPASC.

d Fisher’s exact test, low-burden vs high-burden neuroPASC.

**Supplementary Table 3** – Pre-existing conditions among participants with neuroPASC and controls. Participants with neuroPASC were stratified into two clusters—low-burden and high-burden—based on the burden of self-reported post-COVID neurological symptoms persisting at the time of assessment. Group comparisons were conducted using the Benjamini–Hochberg correction for multiple testing (false discovery rate, FDR < 0.05) across the following contrasts: controls vs. low-burden neuroPASC, controls vs. high-burden neuroPASC, and low-burden vs. high-burden neuroPASC. Sample sizes are indicated where data were missing.

| **Pre-existing conditions, No. (%)** | **Control participants (n=74)** | **Participants with neuroPASC** | | | | ***P value* neuroPASC vs. control participants** | ***P value* three group comparison (Control, low-burden, high-burden neuroPASC)** |
| --- | --- | --- | --- | --- | --- | --- | --- |
|  |  | **Total group (n=102)** | **Low-burden neuroPASC (n=75)** | | **High-burden neuroPASC (n=27)** |  |  |
| Endocrine/metabolic^e^ | 11 (15%) | 31 (30%) | 18 (24%) | 13 (48%) | | **0.02^a^** | 0.2^b^, **0.003^c^**, **0.04^d^** |
| Gastrointestinal/hepatobiliary^f^ | 11 (15%) | 24 (24%) | 12 (16%) | 12 (44%) | | 0.2**^a^** | >0.99^b^, **0.009^c^**, **0.01^d^** |
| Psychiatric^g^ | 20 (27%) | 46 (45%) | 35 (47%) | 11 (41%) | | **0.02^a^** | **0.05**^b^, 0.3**^c^**, 0.7**^d^** |
| Neurological^h^ | 12 (16%) | 24 (24%) | 14 (19%) | 10 (37%) | | 0.3**^a^** | 0.8^b^, 0.1**^c^**, 0.1**^d^** |
| Cardiovascular | 9 (12%) | 20 (20%) | 11 (15%) | 9 (33%) | | 0.2**^a^** | 0.8^b^, 0.06**^c^**, 0.07**^d^** |
| Respiratory | 10 (14%) | 20 (20%) | 12 (16%) | 8 (30%) | | 0.3**^a^** | 0.8^b^, 0.2**^c^**, 0.2**^d^** |
| Musculoskeletal | 13 (18%) | 22 (22%) | 14 (19%) | 8 (30%) | | 0.6**^a^** | >0.99^b^, 0.4**^c^**, 0.4**^d^** |
| Ocular/vision | 9 (12%) | 17 (17%) | 10 (13%) | 7 (26%) | | 0.5**^a^** | >0.99^b^, 0.2**^c^**, 0.2**^d^** |
| Renal/urinary | 5 (7%) | 7 (7%) | 4 (5%) | 3 (11%) | | >0.99**^a^** | 0.7^b^, 0.7**^c^**, 0.7**^d^** |
| Dermatological | 12 (16%) | 11 (11%) | 7 (9%) | 4 (15%) | | 0.4**^a^** | 0.7^b^, >0.99**^c^**, 0.7**^d^** |
| Blood | 4 (5%) | 4 (4%) | 4 (5%) | 0 | | 0.7**^a^** | >0.99^b^, 0.9**^c^**, 0.9**^d^** |
| Reproductive | 3 (4%) | 4 (4%) | 1 (1%) | 3 (11%) | | >0.99**^a^** | 0.4^b^ 0.4**^c^**, 0.2**^d^** |
| Tumor/cancer | 0 | 0 | 0 | 0 | | >0.99**^a^** | >0.99^b^, >0.99**^c^**, >0.99**^d^** |
| Systemic | 1 (1%) | 2 (2%) | 1 (1%) | 1 (4%) | | >0.99**^a^** | >0.99^b^, 0.7**^c^**, 0.7**^d^** |
| Other | 2 (3%) | 3 (3%) | 1 (1%) | 2 (7%) | | >0.99**^a^** | 0.6^b^, 0.4**^c^**, 0.4**^d^** |

a Fisher’s exact test.

b Fisher’s exact test, control vs low-burden neuroPASC.

c Fisher’s exact test, control vs high-burden neuroPASC.

d Fisher’s exact test, low-burden vs high-burden neuroPASC.

**e** Endocrine/metabolic conditions in **controls**: hyperlipidemia (n=5), hypercholesterolemia (n=4), B12 deficiency monitoring (n=1), diabetes (n=1), Gilbert's syndrome (n=1), lower testosterone levels (n=1); **low-burden neuroPASC**: hypothyroidism (n=6), diabetes (n=4), lower testosterone levels (n=2), gout (n=2), hypercholesterolemia (n=2), obesity (n=2), Gilbert’s syndrome (n=1), hyperlipidemia (n=1), pre-diabetes (n=1), thyroid-binding globulin deficiency (n=1), thyroidectomy (n=1), vitamin deficiency (n=1); **high-burden neuroPASC**: hyperlipidemia (n=3), hypothyroidism (n=2), obesity (n=2), diabetes (n=3), prediabetes (n=1), adrenal adenona (n=1), dyslipidemia (n=1), hypercholesterolemia (n=1), mass of thyroid gland (n=1), multinodular goiter (n=1).

**f** Gastrointestinal/hepatobiliary conditions in **controls**: gastroesophageal reflux disease (n=7), Crohn’s disease (n=1), eosinophilic gastritis (n=1), gallstone (n=1), hemorrhoids (n=1), irritable bowel syndrome (n=1), non-alcoholic fatty liver (n=1), reflux gastritis (n=1); **low-burden neuroPASC**: gastroesophageal reflux disease (n=7), steatosis of liver (n=3), irritable bowel syndrome (n=2), gastric reflux (n=3), adenoma of descending colon (n=1), hiatal hernia with gastroesophageal reflux (n=1); **high-burden neuroPASC**: gastroesophageal reflux disease (n=4), irritable bowel syndrome (n=2), esophageal structure (n=1), gastroparesis syndrome (n=1), gluten sensitivity (n=1), hemangioma of liver (n=1), inflammatory bowel disease (n=1), multiple gastric ulcers (n=1), steosis of liver (n=1), ulcerative colitis (n=1).

**g** Psychiatric conditions in **controls**: anxiety (n=9), generalized anxiety disorder (n=4), attention deficit hyperactivity disorder (n=5), depression (n=7), major depressive disorder (n=4), obsessive-compulsive disorder (n=2), personality disorder (n=1); **low-burden neuroPASC**: anxiety (n=19), generalized anxiety disorder (n=3), attention deficit hyperactivity disorder (n=6), undifferentiated attention deficit disorder (n=2), depression (n=9), postpartum depression (n=1), major depressive disorder (n=10), dysthymia (n=1), obsessive-compulsive disorder (n=1), posttraumatic stress disorder (n=1); **high-burden neuroPASC**: anxiety (n=3), generalized anxiety disorder (n=2), attention deficit hyperactivity disorder (n=3), depression (n=2), major depressive disorder (n=3), mixed anxiety and depressive disorder (n=1), posttraumatic stress disorder (n=1).

**h** Neurological conditions in **controls**: autism spectrum disorder (n=1), Bell’s palsy (n=1), insomnia (n=1), migraine (n=7), neuropathy (n=2), spasms of back muscles (n=1), tinnitus (n=1); **low-burden neuroPASC**: headache (n=2), insomnia (n=1), meralgia paresthetica (n=1), migraine (n=9), nystagmus (n=1); **high-burden neuroPASC**: benign intracranial hypertension (n=1), tinnitus (n=1), carpal tunnel syndrome (n=1), history of cerebral aneurysm (n=1), migraine (n=4), neuropathy (n=2), right foot drop (n=1), sciatica (n=1), traumatic brain injury without loss of consciousness (n=1).

**Supplementary Table 4 –** Characteristics of participants with neuroPASC and controls, stratified by hospitalization status during acute infection. Participants with neuroPASC were categorized into non-hospitalized and hospitalized groups. Group comparisons were adjusted for multiple testing using the Benjamini–Hochberg false discovery rate (FDR < 0.05) across the following contrasts: controls vs. non-hospitalized neuroPASC, controls vs. hospitalized neuroPASC, and non-hospitalized vs. hospitalized neuroPASC. Tobacco pack-years was computed as (cigarettes per day/20) × years, assuming that a pack has 20 cigarettes. Alcohol drink-years was computed as (average number of drinks in one day × daily equivalent frequency) × years. Cannabis use-years was computed as daily equivalent frequency × years. MFIS: Modified Fatigue Impact Scale, MFIS _cog: MFIS cognitive subscale, PSQI: Pittsburgh Sleep Quality Index, PHQ-8: Patient Health Questionnaire-8, GAD-7: General Anxiety Disorder-7. Sample sizes are reported where data were missing.

| **Variable** | **Control participants (n=74)** | **Participants with neuroPASC** | | ***P value* three group comparison (Control, Non-hospitalized, Hospitalized neuroPASC)** |
| --- | --- | --- | --- | --- |
|  |  | **neuroPASC**  **Non-hospitalized (n=77)** | **neuroPASC Hospitalized (n=25)** |  |
| Age, median (IQR), y | 43 (28-59) | 46 (33-57) | 58 (51-66) | **0.002**^a^, 0.4^b^, **0.001**^c^, **0.001**^d^ |
| Female, No (%) | 47 (64%) | 56 (72%) | 20 (80%) | 0.4^e^, 0.4^f^, 0.6^g^ |
| Education, median (IQR), y | 16 (16-18) | 16 (14-18) | 16 (14-16) | 0.1^a^, 0.3^b^, 0.07^c^, 0.08^d^ |
| Days since infection, median (IQR), d |  | 742 (449-946) | 949 (641-1230) | **0.007**^d^ |
| Vaccinated for COVID-19, No (%) | 71 (96%) | 72 (94%) | 22 (88%) | 0.7^e^, 0.5^f^, 0.6^g^ |
| Vaccinated for COVID-19 before infection, No (%) | NA | 35 (46%) | 5 (20%) | **0.03**^g^ |
| Behavior history | | | | |
| Tobacco |  |  |  |  |
| Current use, No. (%) | 3 (4%) | 4 (5%) | 1 (4%) | >0.99^e^, >0.99^f^, >0.99^g^ |
| Pack-years, median (IQR) | 0 (0-0) | 0 (0-0) | 0 (0-3.5) | 0.3^h^, 0.7^i^, 0.3^j^, 0.3^k^ |
| Alcohol |  |  |  |  |
| Current use, No. (%) | 56 (76%) | 49 (64%) | 15 (60%) | 0.3^e^, 0.3^f^, 0.8^g^ |
| Past use, No. (%) | 64 (86%) | 65 (84%) | 24 (89%) | 0.8^e^, 0.4^f^, 0.4^g^ |
| Age started drinking, median (IQR) | 19 (18-21) | 18 (17-21) | 17 (15-18) | **0.003**^a^, 0.2^b^, **0.001**^c^, **0.005**^d^ |
| Drink-years, median (IQR) | 1.9 (0.6-7.6) | 2 (0.8-11.5) | 6.2 (2.3-16.4) | 0.3^h^, 0.4^i^, 0.4^j^, 0.4^k^ |
| Cannabis |  |  |  |  |
| Current use, No. (%) | 11 (15%) | 15 (19%) | 4 (16%) | >0.99^e^, >0.99^f^, >0.99^g^ |
| Use-years, median (IQR) | 0 (0-0.2) | 0 (0-0.3) | 0 (0-0.8) | 0.6^h^, 0.7^i^, 0.7^j^, 0.9^k^ |
| BMI, median (IQR) | 25 (23-32) | 28 (24-33), n=75 | 35 (28-41) | **<.001**^h^, .1^i^, **<.001**^j^, **.002**^k^ |
| Blood pressure (mmHg), median (IQR) | | | | |
| Systolic | 121 (112-130) | 121 (110-130) | 124 (109-132) | 0.7^h^, 0.7^i^, 0.7^j^, 0.7^k^ |
| Diastolic | 78 (71-84) | 78 (72-83) | 78 (72-84) | 0.9^h^, 0.9^i^, 0.9^j^, 0.9^k^ |
| HbA1C, %, median (IQR) | 5.2 (4.9-5.6) | 5.2 (5-5.5) | 5.5 (5.3-6.3) | **<0.001**^h^, 0.6^i^, **<0.001**^j^, **0.001**^k^ |
| Quality of life, median (IQR) | | | | |
| MFIS | 28 (22-35) | 68 (47-85), n=76 | 75 (63-86) | **<0.001**^h^, **<0.001**^i^, **<0.001**^j^, 0.3^k^ |
| MFIS_cog | 13 (10-18) | 34 (25-40), n=76 | 35 (30-42) | **<0.001**^h^, **<0.001**^i^, **<0.001**^j^, 0.5^k^ |
| PSQI | 4 (2-6) | 8 (6-11), n=75 | 8 (6-13) | **<0.001**^h^, **<0.001**^i^, **<0.001**^j^, 0.2^k^ |
| PHQ-8 | 2 (1-4) | 8 (4-11), n=72 | 9 (4-12), n=24 | **<0.001**^h^, **<0.001**^i^, **<0.001**^j^, 0.3^k^ |
| GAD-7 | 1 (0-3) | 4 (2-7), n=76 | 3 (1-5) | **<0.001**^h^, **<0.001**^i^, **0.04**^j^, 0.4^k^ |
| Charlson Comorbidity Index, median (IQR) | 0 (0-1) | 0 (0-1) | 2 (1-3) | **<0.001**^a^, 0.3^b^, **<0.001**^c^, **<0.001**^d^ |
| 10-Year Risk Framingham score, median (IQR) | 2.8 (1.2-7.3), n=73 | 3.3 (1.2-8.1), n=76 | 9.4 (4.5-11.7) | **0.02**^a^, .3^b^, **0.007**^c^, **0.01**^d^ |
| Positive for SARS-CoV-2 nucleocapsid antibodies, No. (%) | 37 (51%), n=72 | 70 (91%) | 24 (100%), n=24 | **<0.001**^e^, **<0.001**^f^, 0.3^g^ |
| Presence of APOE ε4 allele, No. (%) | 23 (35%), n=66 | 19 (32%), n=60 | 4 (21%), n=19 | 0.8^e^, 0.8^f^, 0.8^g^ |
| Blood markers, median (IQR) | | | | |
| hsCRP (mg/L) | 1 (0.5-2), n=72 | 1.5 (0.7-2.4) | 3 (0.8-7.4), n=24 | **0.04**^h^, 0.4^i^, **0.03**^j^, 0.07^k^ |
|  |  |  |  | **0.02**^l^**,** 0.9^m^**, 0.02**^n^**, 0.02**^o^ |
| IL-1 (pg/mL) | 0.4 (0.4-0.4), n=72 | 0.4 (0.4-0.4) | 0.4 (0.4-0.5), n=24 | 0.8^h^, 0.9^i^, 0.9^j^, 0.9^k^ |
| IL-6 (pg/mL) | 1.7 (1.5-2.4), n=72 | 1.7 (1.5-2.3), n=76 | 2.9 (1.9-3.8), n=24 | **0.02**^h^, 0.9^i^, **0.02**^j^, **0.02**^k^ |
| TNF-α (pg/mL) | 7.5 (6.3-8.8), n=72 | 7.01 (6.2-8.7) | 8.5 (7.1-9.5), n=24 | 0.8^h^, 0.9^i^, 0.9^j^, 0.9^k^ |
| Aβ42/40 | 0.07 (0.06-0.07), n=72 | 0.07 (0.06-0.07), n=73 | 0.06 (0.06-0.07), n=23 | 0.3^h^, 0.4^i^, 0.8^j^, 0.6^k^ |
| pTau181 (pg/mL) | 18.4 (15-23), n=72 | 18.2 (14-24), n=76 | 18.1 (14-26), n=24 | 0.3^h^, 0.9^i^, 0.2^j^, 0.2^k^ |
| NfL (pg/mL) | 6.9 (4.7-10.8), n=72 | 6.3 (4.7-8.9) | 10.4 (7.8-13), n=24 | 0.9^h^, 0.9^i^, 0.9^j^, 0.9^k^ |
| GFAP (pg/mL) | 58.3 (39.5-84), n=72 | 52.2 (42-72.1) | 81.2 (58.6-94.6) | 0.7^h^, 0.8^i^, 0.8^j^, 0.8^k^ |

a Kruskal-Wallis rank sum test.

b Dunn adjusted pairwise comparison, control vs neuroPASC non-hospitalized.

c Dunn adjusted pairwise comparison, control vs neuroPASC hospitalized.

d Dunn adjusted pairwise comparison, non-hospitalized vs hospitalized neuroPASC.

e Fisher’s exact test, control vs neuroPASC non-hospitalized.

f Fisher’s exact test, control vs neuroPASC hospitalized.

g Fisher’s exact test, non-hospitalized vs hospitalized neuroPASC.

h ANCOVA 2 degree-of-freedom F-test, adjusted for age and sex.

i Pairwise comparison, control vs neuroPASC non-hospitalized, adjusted for age and sex.

j Pairwise comparison, control vs neuroPASC hospitalized, adjusted for age and sex.

k Pairwise comparison, non-hospitalized vs hospitalized neuroPASC, adjusted for age and sex.

l ANCOVA 2 degree of freedom F-test, adjusted for age, sex and group interaction with BMI.

m Pairwise comparison, control vs non-hospitalized neuroPASC, adjusted for age, sex and group interaction with BMI.

n Pairwise comparison, control vs hospitalized neuroPASC, adjusted for age, sex and group interaction with BMI.

o Pairwise comparison, non-hospitalized vs hospitalized neuroPASC, adjusted for age, sex and group interaction with BMI.

**Supplementary Table 5** – Cognitive function analyses among controls and participants with neuroPASC stratified into low-burden and high-burden clusters. Group differences were evaluated using ANCOVA with age and sex as covariates, followed by pairwise comparisons between all groups. Cohen’s d effect sizes were calculated from covariance-corrected residuals adjusted for age and sex. Multiple testing was controlled using the Benjamini–Hochberg false discovery rate (FDR < 0.05) across 36 comparisons (3 groups × 12 cognitive measures). Abbreviations: p (unc.) – uncorrected p-value; pFDR – FDR-adjusted p-value; CI – confidence interval; C – control; LB – low-burden neuroPASC; HB – high-burden neuroPASC.

|  |  | Low-burden neuroPASC vs. Controls | | | | High-burden neuroPASC vs. Controls | | | | High-burden vs. Low-burden neuroPASC | | | |
| --- | --- | --- | --- | --- | --- | --- | --- | --- | --- | --- | --- | --- | --- |
|  | N | t | p  (unc.) | pFDR | Cohen’s d  (CI) | t | p  (unc.) | pFDR | Cohen’s d  (CI) | t | p  (unc.) | pFDR | Cohen’s d  (CI) |
| MoCA | C=73  LB=73  HB=26 | -2.06 | 0.04 | 0.09 | -0.35  (-0.68, -0.02) | -3.12 | 0.002 | **0.02** | -0.72  (-1.19, -0.26) | -1.68 | 0.09 | 0.2 | -0.32  (-0.78, 0.13) |
| BVMT-R DR | C=73  LB=73  HB=25 | -1.44 | 0.2 | 0.2 | -0.25  (-0.57, 0.08) | -2.35 | 0.02 | 0.06 | -0.55  (-1.01, -0.09) | -1.37 | 0.2 | 0.3 | -0.26  (-0.73, 0.2) |
| BVMT-R total | C=73  LB=73  HB=25 | -2.08 | 0.04 | 0.09 | -0.36  (-0.69, -0.03) | -1.62 | 0.1 | 0.2 | -0.36  (-0.82, 0.11) | -0.18 | 0.9 | 0.9 | -0.02  (-0.48, 0.44) |
| HVLT-R DR | C=73  LB=73  HB=26 | -0.79 | 0.4 | 0.6 | -0.14  (-0.46, 0.19) | -3.29 | 0.001 | **0.01** | -0.69  (-1.16, -0.23) | -2.76 | 0.006 | **0.03** | -0.56  (-1.02, -0.1) |
| HVLT-R total | C=73  LB=73  HB=26 | -1.47 | 0.1 | 0.2 | -0.25  (-0.58, 0.08) | -2.53 | 0.01 | **0.04** | -0.55  (-1.01, -0.09) | -1.51 | 0.1 | 0.2 | -0.31  (-0.77, 0.14) |
| Digit Span (backward) total | C=73  LB=73  HB=26 | -0.22 | 0.8 | 0.9 | -0.04  (-0.36, 0.29) | -0.23 | 0.8 | 0.9 | -0.05  (-0.5, 0.4) | -0.07 | 0.9 | 0.9 | -0.01  (-0.47, 0.44) |
| Digit Span total | C=73  LB=73  HB=26 | -1.07 | 0.3 | 0.4 | -0.18  (-0.5, 0.15) | -1.06 | 0.3 | 0.4 | -0.23  (-0.68, 0.23) | -0.3 | 0.8 | 0.9 | -0.06  (-0.52, 0.39) |
| COWA-FAS total | C=73  LB=73  HB=26 | -1.23 | 0.2 | 0.3 | -0.19  (-0.52, 0.14) | -1.56 | 0.1 | 0.2 | -0.33  (-0.79, 0.12) | -0.7 | 0.5 | 0.6 | -0.17  (-0.62, 0.29) |
| Fruits/  Furniture total | C=73  LB=73  HB=26 | 0.5 | 0.6 | 0.7 | 0.09  (-0.24, 0.42) | -2.23 | 0.03 | 0.07 | -0.49  (-0.95, -0.03) | -2.61 | 0.01 | **0.04** | -0.59  (-1.05, -0.13) |
| Stroop Color-Word | C=72  LB=73  HB=24 | -2.75 | 0.007 | **0.03** | -0.43  (-0.76, -0.1) | -3.5 | 0.0006 | **0.01** | -0.79  (-1.28, -0.31) | -1.64 | 0.1 | 0.2 | -0.38  (-0.85, 0.09) |
| Stroop Interference | C=72  LB=73  HB=24 | -3.4 | 0.0008 | **0.01** | -0.55  (-0.88, -0.21) | -3.01 | 0.003 | **0.02** | -0.69  (-1.16, -0.21) | -0.7 | 0.5 | 0.6 | -0.14  (-0.6, 0.33) |
| SDMT | C=73  LB=73  HB=26 | -2.41 | 0.02 | 0.06 | -0.39  (-0.72, -0.06) | -4.02 | 7.5E-05 | **0.003** | -0.8  (-1.27, -0.34) | -2.39 | 0.02 | 0.06 | -0.59  (-1.05, -0.13) |

**Supplementary Table 6** – Cognitive function analyses among controls and participants with neuroPASC, stratified by hospitalization status during acute infection. Group differences were evaluated using ANCOVA with age and sex as covariates, followed by pairwise comparisons between all groups. Cohen’s d effect sizes were calculated from covariance-corrected residuals adjusted for age and sex. Multiple testing was controlled using the Benjamini–Hochberg false discovery rate (FDR < 0.05) across 36 comparisons (3 groups × 12 cognitive measures). Abbreviations: p (unc.) – uncorrected p-values, pFDR – adjusted p-value for multiple testing, CI – confidence interval, C – control, NH – non-hospitalized, H – hospitalized.

|  |  | Non-hospitalized neuroPASC vs. Controls | | | | Hospitalized neuroPASC vs. Controls | | | | Hospitalized vs. Non-hospitalized neuroPASC | | | |
| --- | --- | --- | --- | --- | --- | --- | --- | --- | --- | --- | --- | --- | --- |
|  | N | t | p  (unc.) | pFDR | Cohen’s d  (CI) | t | p  (unc.) | pFDR | Cohen’s d  (CI) | t | p  (unc.) | pFDR | Cohen’s d  (CI) |
| MoCA | C=73,  NH=75,  H=24 | -2.04 | 0.04 | 0.1 | -0.33  (-0.66, -0.002) | -3.32 | 0.001 | **0.02** | -0.85  (-1.33, -0.37) | -1.98 | 0.05 | 0.1 | -0.39  (-0.86, 0.08) |
| BVMT-R DR | C=73,  NH=74,  H=24 | -2.24 | 0.03 | 0.1 | -0.38  (-0.71, -0.05) | -0.48 | 0.6 | 0.8 | -0.13  (-0.59, 0.34) | 1.05 | 0.3 | 0.5 | 0.23  (-0.24, 0.7) |
| BVMT-R total | C=73,  NH=74,  H=24 | -2.33 | 0.02 | 0.1 | -0.38  (-0.71, -0.05) | -1.04 | 0.3 | 0.5 | -0.26  (-0.73, 0.21) | 0.54 | 0.6 | 0.8 | 0.14  (-0.33, 0.6) |
| HVLT-R DR | C=73,  NH=75,  H=24 | -1.26 | 0.2 | 0.4 | -0.21  (-0.53, 0.12) | -2.28 | 0.02 | 0.1 | -0.55  (-1.02, -0.08) | -1.45 | 0.2 | 0.3 | -0.29  (-0.76, 0.18) |
| HVLT-R total | C=73,  NH=75,  H=24 | -1.7 | 0.09 | 0.2 | -0.28  (-0.6, 0.05) | -2.04 | 0.04 | 0.1 | -0.49  (-0.96, -0.02) | -0.91 | 0.4 | 0.6 | -0.18  (-0.65, 0.29) |
| Digit Span (backward) total | C=73,  NH=75,  H=24 | -0.38 | 0.7 | 0.8 | -0.06  (-0.39, 0.26) | 0.13 | 0.9 | 0.9 | 0.03  (-0.44, 0.5) | 0.39 | 0.7 | 0.8 | 0.09  (-0.37, 0.56) |
| Digit Span total | C=73,  NH=75,  H=24 | -1.36 | 0.2 | 0.4 | -0.22  (-0.54, 0.11) | -0.39 | 0.7 | 0.8 | -0.09  (-0.56, 0.38) | 0.54 | 0.6 | 0.8 | 0.14  (-0.33, 0.61) |
| COWA-FAS total | C=73,  NH=75,  H=24 | -1.65 | 0.1 | 0.2 | -0.27  (-0.6, 0.06) | -0.61 | 0.5 | 0.8 | -0.13  (-0.6, 0.34) | 0.5 | 0.6 | 0.8 | 0.15  (-0.32, 0.61) |
| Fruits/  Furniture total | C=73,  NH=75,  H=24 | -0.34 | 0.7 | 0.8 | -0.05  (-0.38, 0.27) | -0.37 | 0.7 | 0.8 | -0.09  (-0.55, 0.38) | -0.14 | 0.9 | 0.9 | -0.03  (-0.5, 0.44) |
| Stroop Color-Word | C=72,  NH=73,  H=24 | -3.01 | 0.003 | **0.02** | -0.49  (-0.82, -0.16) | -2.83 | 0.005 | **0.03** | -0.61  (-1.09, -0.14) | -0.79 | 0.4 | 0.7 | -0.16  (-0.63, 0.31) |
| Stroop Interference | C=72,  NH=73,  H=24 | -3.25 | 0.001 | **0.02** | -0.53  (-0.86, -0.19) | -3.42 | 0.0008 | **0.02** | -0.75  (-1.23, -0.27) | -1.23 | 0.2 | 0.4 | -0.26  (-0.73, 0.21) |
| SDMT | C=73,  NH=75,  H=24 | -3.08 | 0.002 | **0.02** | -0.49  (-0.82, -0.16) | -2.46 | 0.01 | 0.08 | -0.53  (-1, -0.05) | -0.4 | 0.7 | 0.8 | -0.07  (-0.54, 0.4) |

**Supplementary Table 7 –** Demographic characteristics of neuroPASC and control groups after excluding participants younger than 30 years. Group comparisons were adjusted for multiple testing using the Benjamini–Hochberg false discovery rate (FDR < 0.05) across the following contrasts: controls vs. low-burden neuroPASC, controls vs. high-burden neuroPASC, and low-burden vs. high-burden neuroPASC.

|  | **Control (n=51)** | **Low-burden neuroPASC (n=57)** | **High-burden neuroPASC (n=27)** | ***P value* three group comparison (Control, low-burden, high-burden neuroPASC)** |
| --- | --- | --- | --- | --- |
| Age, median (IQR), y | 55 (42-62) | 52 (42-60) | 56 (47-64) | 0.4^a^, 0.3^b^, 0.4^c^, 0.3^d^ |
| Females, No. (%) | 31 (61%) | 41 (72%) | 20 (74%) | 0.5^e^, 0.5^f^, >0.99^g^ |
| Education, median (IQR), y | 16 (16-18) | 16 (14-18) | 16 (14-18) | 0.3^a^, 0.2^b^, 0.2^c^, 0.3^d^ |

a Kruskal-Wallis rank sum test.

b Mann-Whitney U test, control vs low-burden neuroPASC.

c Mann-Whitney U test, control vs high-burden neuroPASC.

d Mann-Whitney U test low-burden vs high-burden neuroPASC.

e Fisher’s exact test, control vs low-burden neuroPASC.

f Fisher’s exact test, control vs high-burden neuroPASC.

g Fisher’s exact test, low-burden vs high-burden neuroPASC.

**Supplementary Table 8** - Cognitive function analyses in age-matched neuroPASC and control groups (participants aged ≥30 years). Group differences were evaluated using ANCOVA with age and sex as covariates, followed by pairwise comparisons between all groups. Cohen’s d effect sizes were calculated from covariance-corrected residuals adjusted for age and sex. p-values were corrected for multiple testing using the false discovery rate (FDR) across 36 comparisons (3 groups × 12 cognitive measures). Abbreviations: p (unc.) – uncorrected p-value; pFDR – FDR-adjusted p-value; CI – confidence interval; C – control; LB – low-burden neuroPASC; HB – high-burden neuroPASC.

|  |  | Low-burden neuroPASC vs. Controls | | | | High-burden neuroPASC vs. Controls | | | | High-burden vs. Low-burden neuroPASC | | | |
| --- | --- | --- | --- | --- | --- | --- | --- | --- | --- | --- | --- | --- | --- |
|  | N | t | p  (unc.) | pFDR | Cohen’s d  (CI) | t | p  (unc.) | pFDR | Cohen’s d  (CI) | t | p  (unc.) | pFDR | Cohen’s d  (CI) |
| MoCA | C=50  LB=55  HB=26 | -2.74 | 0.007 | **0.04** | -0.56  (-0.96, -0.17) | -3.3 | 0.001 | **0.02** | -0.87  (-1.37, -0.37) | -1.11 | 0.3 | 0.4 | -0.23  (-0.71, 0.24) |
| BVMT-R DR | C=50  LB=55  HB=25 | -1.41 | 0.2 | 0.3 | -0.29  (-0.68, 0.1) | -2.21 | 0.03 | 0.07 | -0.55  (-1.05, -0.06) | -1.12 | 0.3 | 0.4 | -0.25  (-0.73, 0.24) |
| BVMT-R total | C=50  LB=55  HB=25 | -2.17 | 0.03 | 0.08 | -0.44  (-0.83, -0.05) | -1.62 | 0.1 | 0.2 | -0.38  (-0.88, 0.11) | 0.1 | 0.9 | 0.9 | -0.03  (-0.45, 0.51) |
| HVLT-R DR | C=50  LB=55  HB=26 | -1.16 | 0.2 | 0.4 | -0.25  (-0.64, 0.14) | -3.14 | 0.002 | **0.02** | -0.72  (-1.22, -0.22) | -2.25 | 0.03 | 0.07 | -0.5  (-0.98, -0.02) |
| HVLT-R total | C=50  LB=55  HB=25 | -1.71 | 0.09 | 0.2 | -0.35  (-0.74, 0.04) | -2.57 | 0.01 | **0.05** | -0.6  (-1.09, -0.11) | -1.21 | 0.2 | 0.4 | -0.28  (-0.76, 0.2) |
| Digit Span (backward) total | C=50  LB=55  HB=26 | 0.57 | 0.6 | 0.7 | 0.11  (-0.28, 0.5) | 0.23 | 0.8 | 0.9 | 0.06  (-0.42, 0.54) | -0.24 | 0.8 | 0.9 | -0.05  (-0.53, 0.42) |
| Digit Span total | C=50  LB=55  HB=26 | -0.38 | 0.7 | 0.8 | -0.08  (-0.46, 0.31) | -0.77 | 0.5 | 0.6 | -0.18  (-0.66, 0.3) | -0.46 | 0.6 | 0.8 | -0.11  (-0.59, 0.36) |
| COWA-FAS total | C=50  LB=55  HB=26 | -0.85 | 0.4 | 0.6 | -0.16  (-0.55, 0.23) | -1.42 | 0.2 | 0.3 | -0.33  (-0.82, 0.15) | -0.74 | 0.5 | 0.6 | -0.2  (-0.67, 0.28) |
| Fruits/  Furniture total | C=50  LB=55  HB=26 | -0.15 | 0.9 | 0.9 | -0.02  (-0.37, 0.341) | -2.44 | 0.02 | 0.06 | -0.56  (-1.05, -0.07) | -2.45 | 0.02 | 0.06 | -0.59  (-1.07, -0.1) |
| Stroop Color-Word | C=49  LB=55  HB=24 | -2.18 | 0.03 | 0.08 | -0.42  (-0.82, -0.03) | -3.31 | 0.001 | **0.02** | -0.85  (-1.37, -0.34) | -1.62 | 0.1 | 0.2 | -0.41  (-0.9, 0.08) |
| Stroop Interference | C=49  LB=55  HB=24 | -2.84 | 0.005 | **0.03** | -0.54  (-0.94, -0.14) | -2.8 | 0.006 | **0.03** | -0.7  (-1.21, -0.19) | -0.6 | 0.5 | 0.7 | -0.15  (-0.64, 0.34) |
| SDMT | C=50  LB=55  HB=26 | -2.32 | 0.02 | 0.07 | -0.45  (-0.84, -0.06) | -4 | 0.0001 | **0.004** | -0.85  (-1.35, -0.35) | -2.14 | 0.04 | 0.08 | -0.59  (-1.08, -0.11) |

**Supplementary Table 9 –** Cognitive function analyses by SARS-CoV-2 nucleocapsid antibody status. Controls who tested positive for SARS-CoV-2 nucleocapsid antibodies were compared with antibody-negative controls. Participants in the low-burden and high-burden neuroPASC clusters who tested positive for nucleocapsid antibodies were compared with both antibody-negative and antibody-positive controls. Group differences were assessed using ANCOVA with age and sex as covariates, followed by pairwise comparisons between groups. p-values were corrected for multiple testing using the false discovery rate (FDR) across 60 comparisons (5 group contrasts × 12 cognitive measures). Abbreviations: pFDR – FDR-adjusted p-value; CI – confidence interval; CN – antibody-negative control; CP – antibody-positive control; LB – low-burden neuroPASC; HB – high-burden neuroPASC.

|  |  | Controls (Negative) vs Controls (Positive) | | | Low-burden neuroPASC (Positive) vs. Controls (Negative) | | | High-burden neuroPASC (Positive) vs. Controls (Negative) | | | Low-burden neuroPASC (Positive) vs. Controls (Positive) | | | High-burden neuroPASC (Positive) vs. Controls (Positive) | | |
| --- | --- | --- | --- | --- | --- | --- | --- | --- | --- | --- | --- | --- | --- | --- | --- | --- |
|  | N | t | pFDR | Cohen’s d  (CI) | t | pFDR | Cohen’s d  (CI) | t | pFDR | Cohen’s d  (CI) | t | pFDR | Cohen’s d  (CI) | t | pFDR | Cohen’s d  (CI) |
| MoCA | CN=35  CP=37  LB=69  HB=23 | -1.7 | 0.2 | -0.53  (-1.01, -0.05) | -2.7 | **0.03** | -0.59  (-1.01, -0.17) | -3.7 | **0.004** | -0.98  (-1.55, -0.42) | -0.8 | 0.6 | -0.15  (-0.55, 0.26) | -2.1 | 0.09 | -0.54  (-1.08, 0.003) |
| BVMT-R DR | CN=35  CP=37  LB=69  HB=22 | 0.2 | 0.9 | 0.009  (-0.46, 0.48) | -0.9 | 0.5 | -0.21  (-0.62, 0.2) | -2.6 | **0.04** | -0.65  (-1.21, -0.09) | -1.2 | 0.4 | -0.22  (-0.63, 0.18) | -2.7 | **0.03** | -0.69  (-1.25, -0.14) |
| BVMT-R total | CN=35  CP=37  LB=69  HB=22 | 0.2 | 0.9 | 0.01  (-0.46, 0.48) | -1.6 | 0.2 | -0.36  (-0.77, 0.06) | -2.1 | 0.09 | -0.52  (-1.07, 0.03) | -1.8 | 0.2 | -0.37  (-0.77, 0.04) | -2.2 | 0.08 | -0.53  (-1.07, 0.02) |
| HVLT-R DR | CN=35  CP=37  LB=69  HB=23 | -0.8 | 0.6 | -0.27  (-0.75, 0.20) | -1.4 | 0.3 | -0.33  (-0.74, 0.08) | -3.8 | **0.004** | -0.91  (-1.47, -0.34) | -0.4 | 0.8 | -0.07  (-0.48, 0.33) | -3.0 | **0.02** | -0.68  (-1.23, -0.14) |
| HVLT-R total | CN=35  CP=37  LB=69  HB=23 | -0.7 | 0.6 | -0.20  (-0.67, 0.27) | -1.7 | 0.2 | -0.39  (-0.8, 0.03) | -2.9 | **0.02** | -0.74  (-1.29, -0.18) | -1.0 | 0.5 | -0.19  (-0.59, 0.22) | -2.3 | 0.07 | -0.56  (-1.10, -0.02) |
| Digit Span (backward) total | CN=35  CP=37  LB=69  HB=23 | -0.3 | 0.9 | -0.06  (-0.54, 0.41) | -0.4 | 0.8 | -0.08  (-0.49, 0.33) | -0.5 | 0.7 | -0.15  (-0.68, 0.39) | -0.1 | 0.9 | -0.02  (-0.42, 0.38) | -0.3 | 0.8 | -0.08  (-0.61, 0.45) |
| Digit Span total | CN=35  CP=37  LB=69  HB=23 | -1.0 | 0.4 | -0.25  (-0.72, 0.22) | -1.5 | 0.2 | -0.32  (-0.74, 0.09) | -1.8 | 0.2 | -0.46  (-1.01, 0.08) | -0.3 | 0.8 | -0.06  (-0.47, 0.34) | -0.8 | 0.6 | -0.21  (-0.75, 0.32) |
| COWA-FAS total | CN=35  CP=37  LB=69  HB=23 | -3.0 | **0.02** | -0.64  (-1.12, -0.16) | -2.8 | **0.03** | -0.54  (-0.95, -0.12) | -2.5 | **0.048** | -0.61  (-1.16, -0.06) | 0.8 | 0.6 | 0.15  (-0.26, 0.55) | 0.2 | 0.8 | 0.04  (-0.49, 0.57) |
| Fruits/  Furniture total | CN=35  CP=37  LB=69  HB=23 | -2.8 | **0.02** | -0.69  (-1.17, -0.20) | -1.0 | 0.5 | -0.19  (-0.61, 0.22) | -3.6 | **0.004** | -0.92  (-1.49, -0.36) | 2.4 | 0.06 | 0.49  (0.08, 0.9) | -1.0 | 0.5 | -0.30  (-0.84, 0.23) |
| Stroop Color-Word | CN=35  CP=36  LB=69  HB=21 | -1.6 | 0.2 | -0.39  (-0.87, 0.09) | -3.0 | **0.02** | -0.65  (-1.07, -0.23) | -3.9 | **0.004** | -1.25  (-1.85, -0.65) | -1.1 | 0.4 | -0.20  (-0.61, 0.21) | -2.4 | 0.06 | -0.62  (-1.18, -0.05) |
| Stroop Interference | CN=35  CP=36  LB=69  HB=21 | -1.7 | 0.2 | -0.43  (-0.91, 0.05) | -3.7 | **0.004** | -0.80  (-1.22, -0.37) | -3.4 | **0.007** | -0.99  (-1.57, -0.4) | -1.7 | 0.2 | -0.34  (-0.75, 0.07) | -1.8 | 0.17 | -0.49  (-1.05, 0.07) |
| SDMT | CN=35  CP=37  LB=69  HB=23 | -0.4 | 0.8 | -0.14  (-0.61, 0.33) | -2.2 | 0.08 | -0.54  (-0.95, -0.12) | -4.1 | **0.004** | -1.09  (-1.66, -0.51) | -1.7 | 0.2 | -0.32  (-0.73, 0.09) | -3.6 | **0.004** | -0.80  (-1.35, -0.25) |

# Supplementary Figures


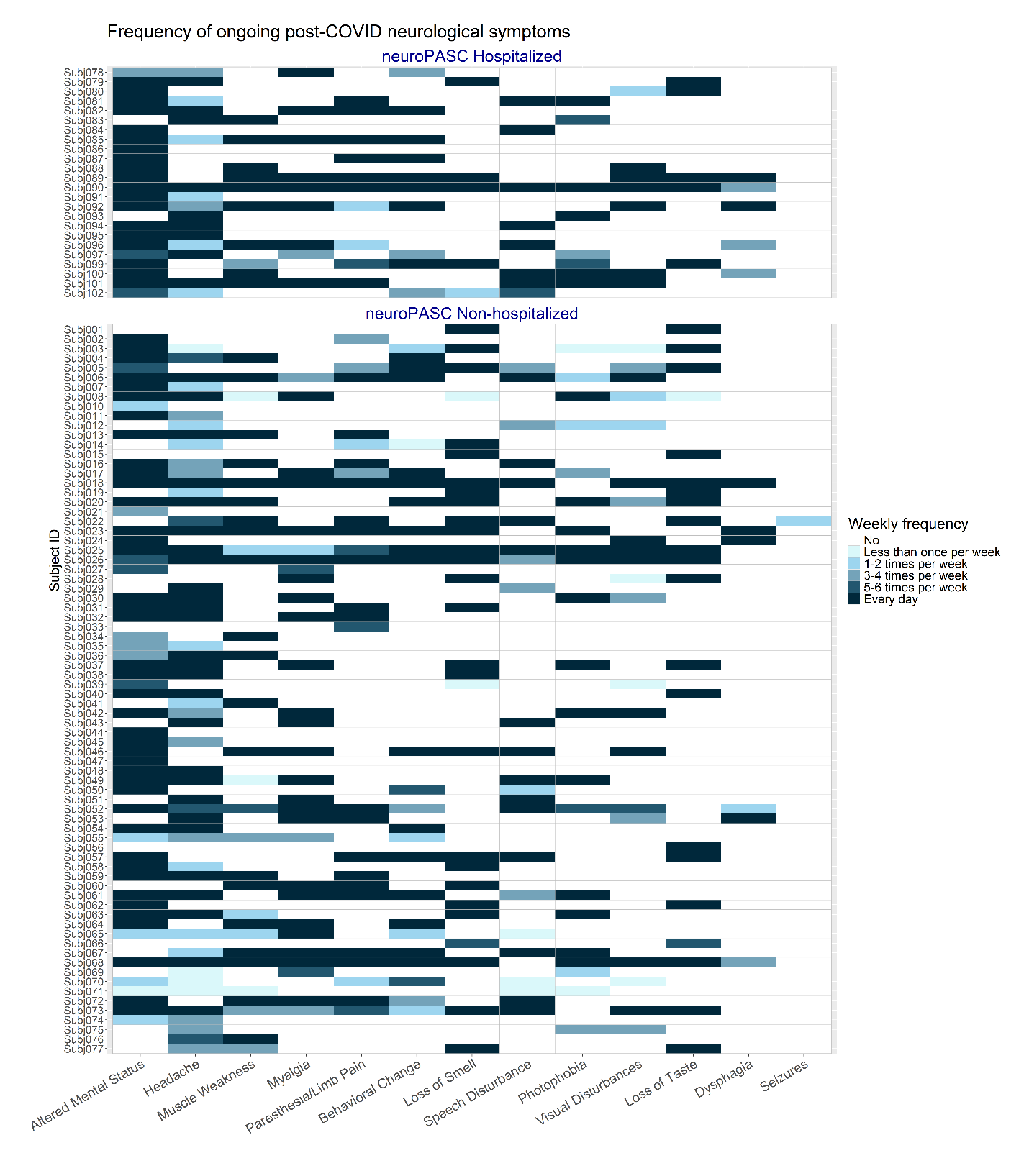


**Supplementary Figure 1** – Frequency of ongoing post-COVID neurological symptoms in participants with neuroPASC. Subject-level heatmaps are color-coded by weekly frequency of each symptom when most high-burden. Only ongoing symptoms, those still present within the 14 days before the study visit, are included.


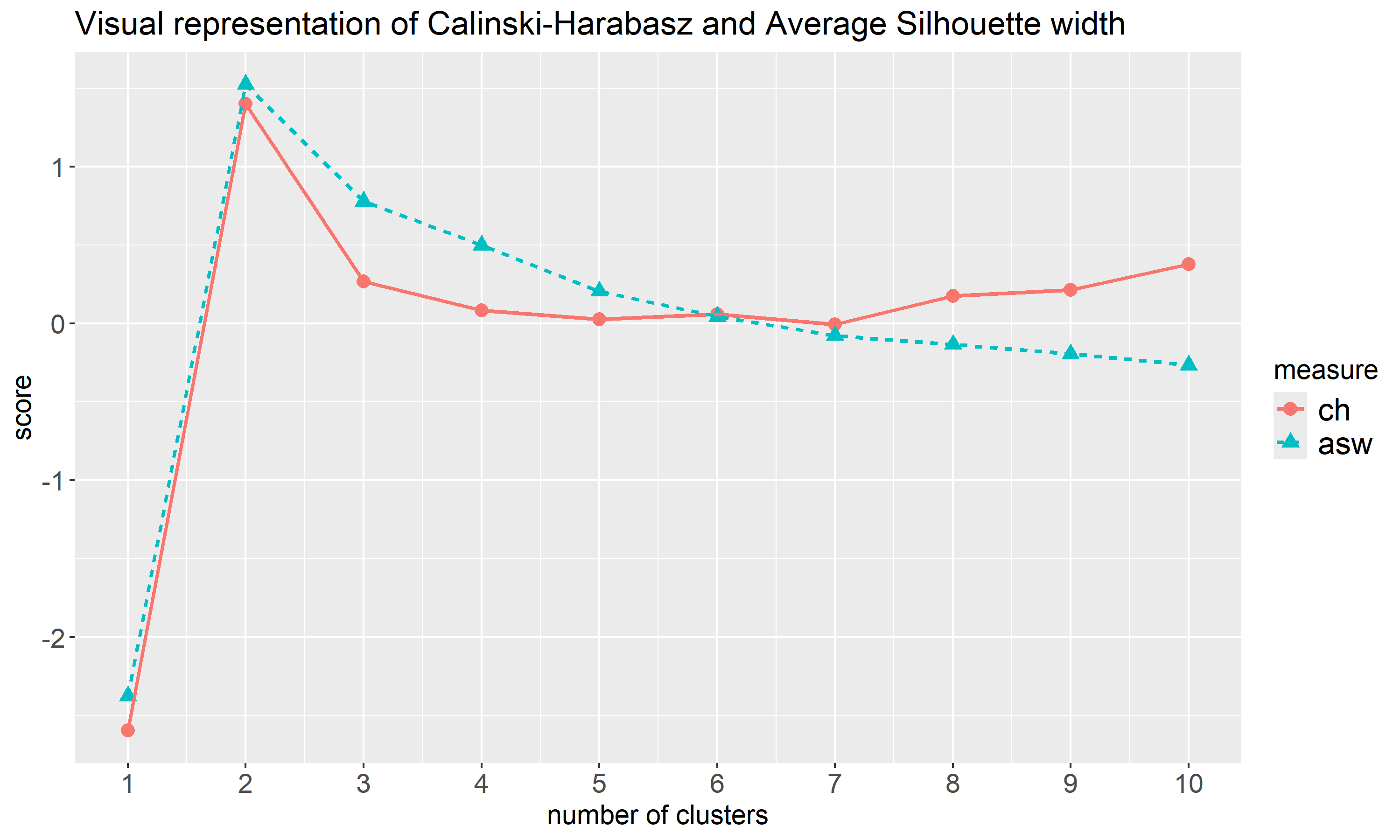


**Supplementary Figure 2** – Visual representation of Calinski-Harabasz and average silhouette width scores obtained by K-means clustering with different number of clusters on neuroPASC neurological symptoms.


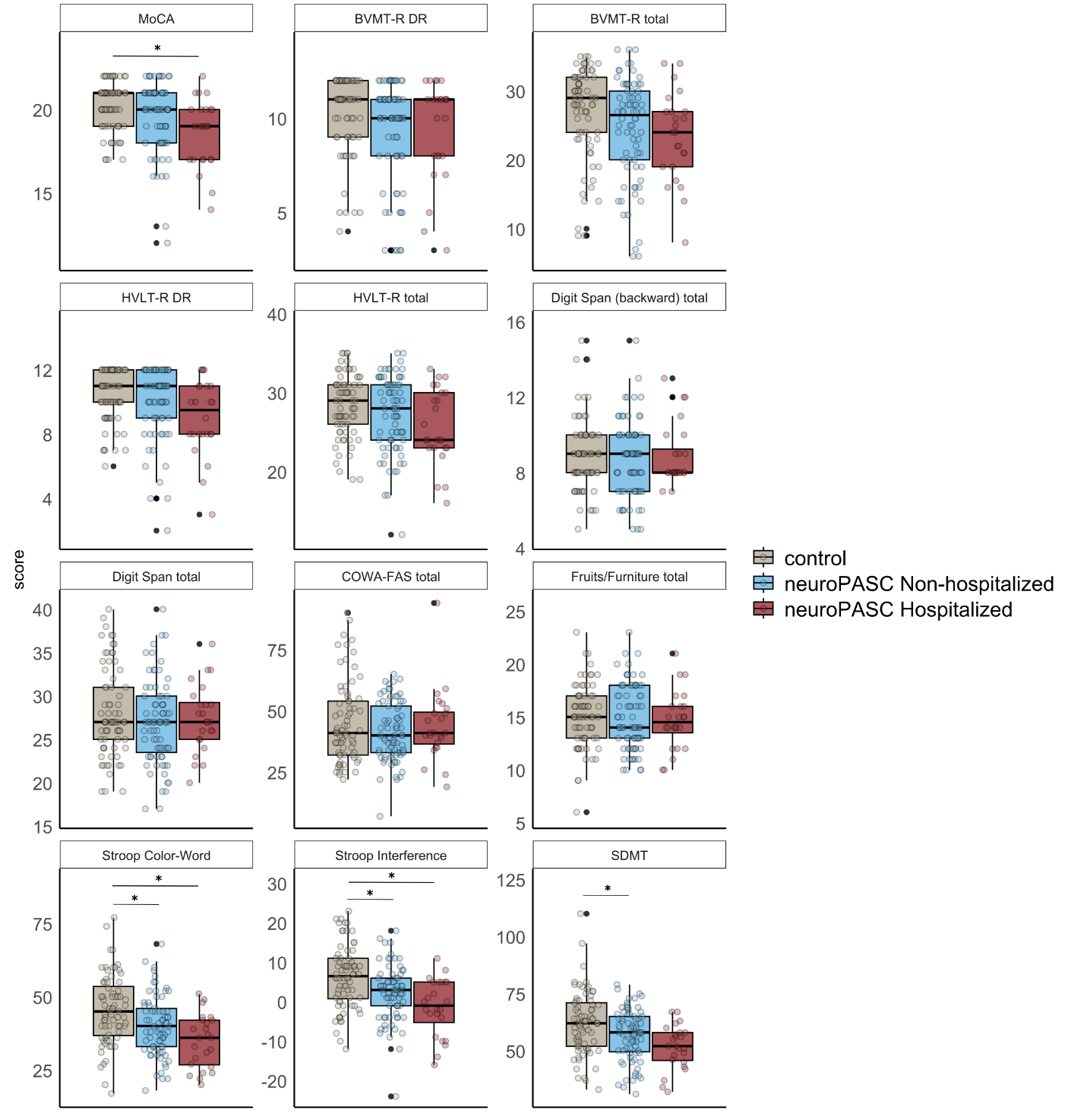


**Supplementary Figure 3** – Boxplots of neurocognitive findings in non-hospitalized and hospitalized participants with neuroPASC and controls. Group differences were assessed using ANCOVA followed by pairwise comparisons, adjusting for age and sex. Statistics are documented in Supplementary Supplementary Table 6. *p<0.05; p-values were adjusted for multiple testing using false discovery rate correction (3x12=36 comparisons). Black dots indicate outliers, which were retained in the analyses. MoCA: Montreal Cognitive Assessment, BVMT-R: Brief Visuospatial Memory Test Revised, HVLT-R: Hopkins Verbal Learning Test- Revised, DR: delay recall, COWA-FAS: Controlled Oral Word Association Test, SDMT: Symbol Digit Modalities Test.


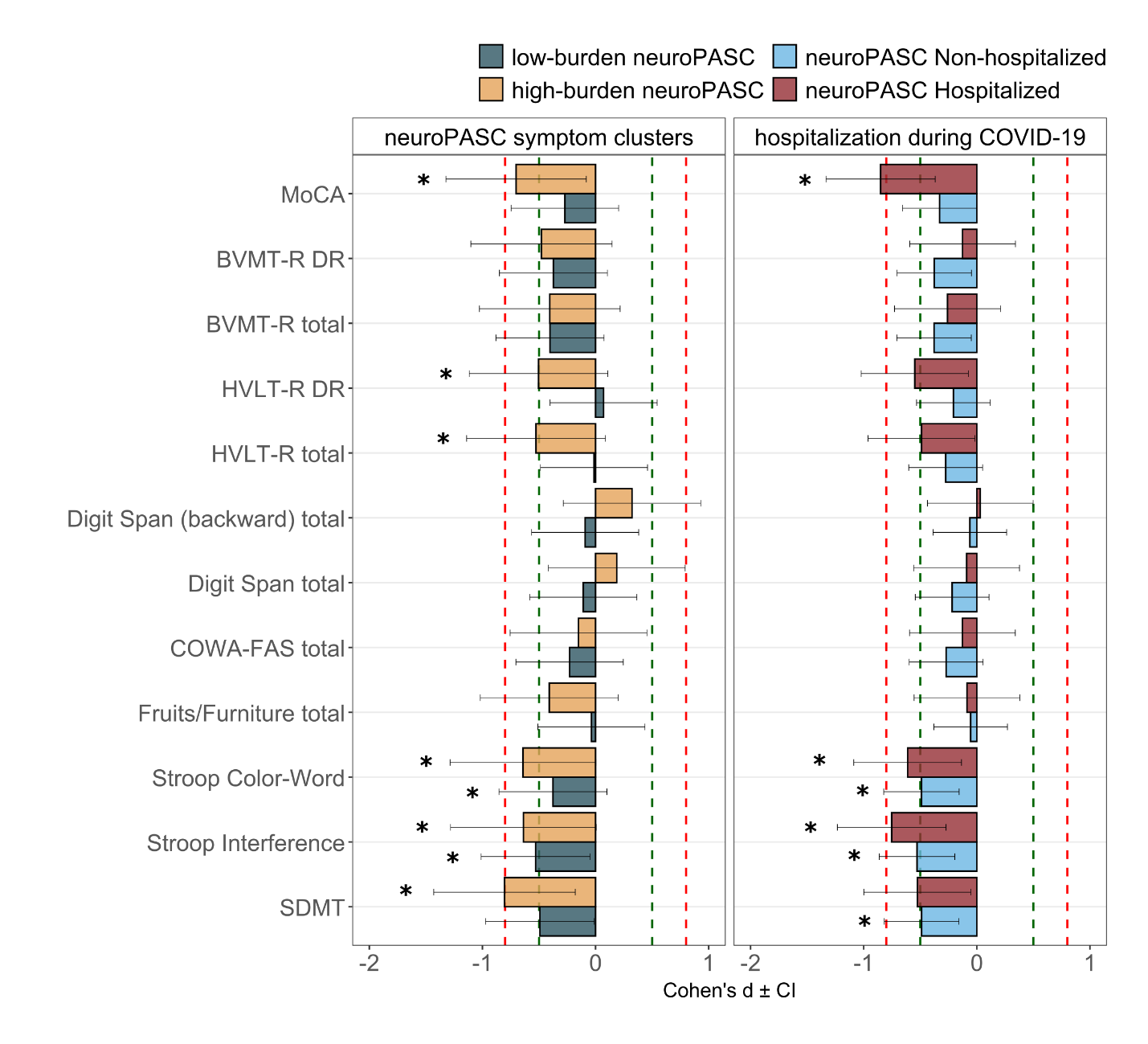


**Supplementary Figure 4** – Bar plot showing Cohen’s d effect sizes for comparisons of neurocognitive tests between each neuroPASC group and controls, with participants grouped by post-COVID neurological symptom burden clusters (left) or by hospitalization status during acute COVID-19 (right). The thresholds for when an effect size is considered to be medium (0.5) or large (0.8), according to Cohen’s criteria, are represented by the vertical green and red dashed lines, respectively. Cognitive tests where significant group differences were found using ANCOVA (Supplementary Table 5 and Supplementary Table 6) are noted by an asterisk (*). CI – 95% confidence interval. MoCA: Montreal Cognitive Assessment, BVMT-R: Brief Visuospatial Memory Test Revised, HVLT-R: Hopkins Verbal Learning Test- Revised, DR: delay recall, COWA-FAS: Controlled Oral Word Association Test, SDMT: Symbol Digit Modalities Test.


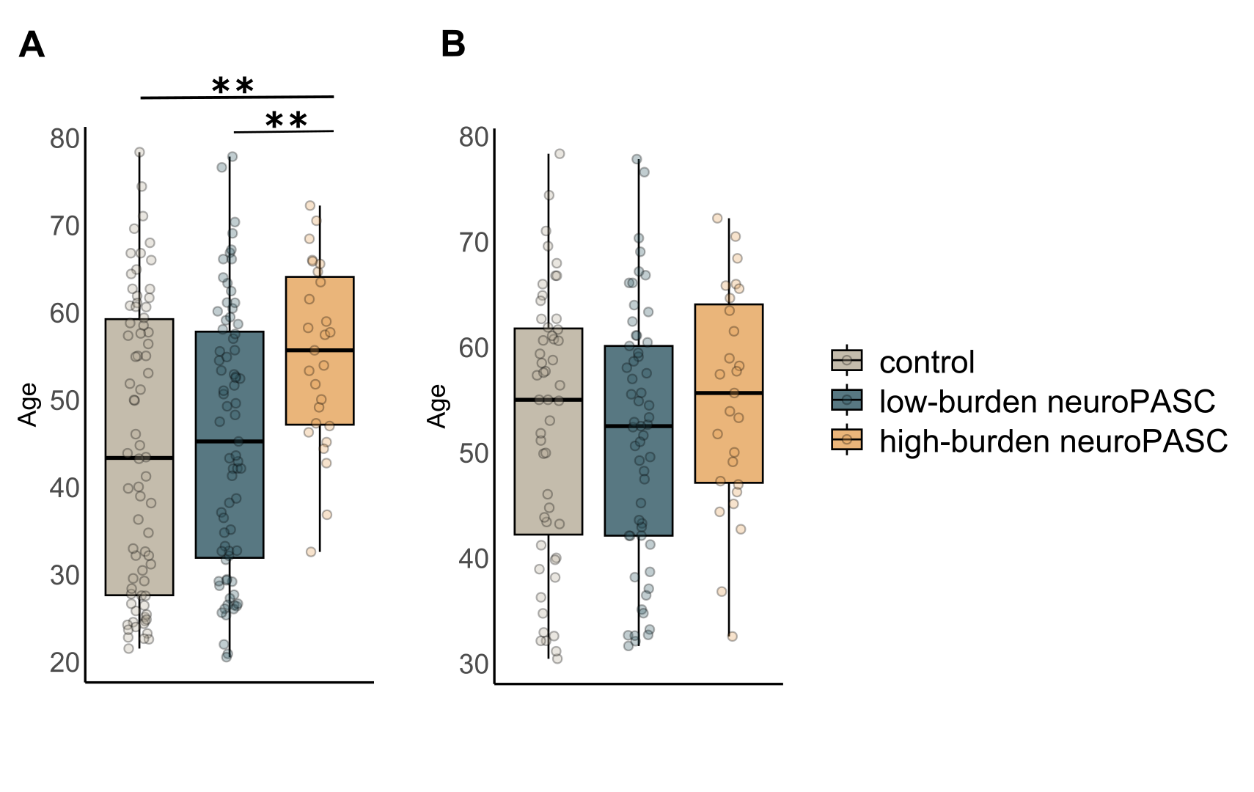
**Supplementary Figure 5** - Age differences between controls and neuroPASC clusters**. A)** The entire cohort; **B)** Subset of individuals who are 30 years or older.


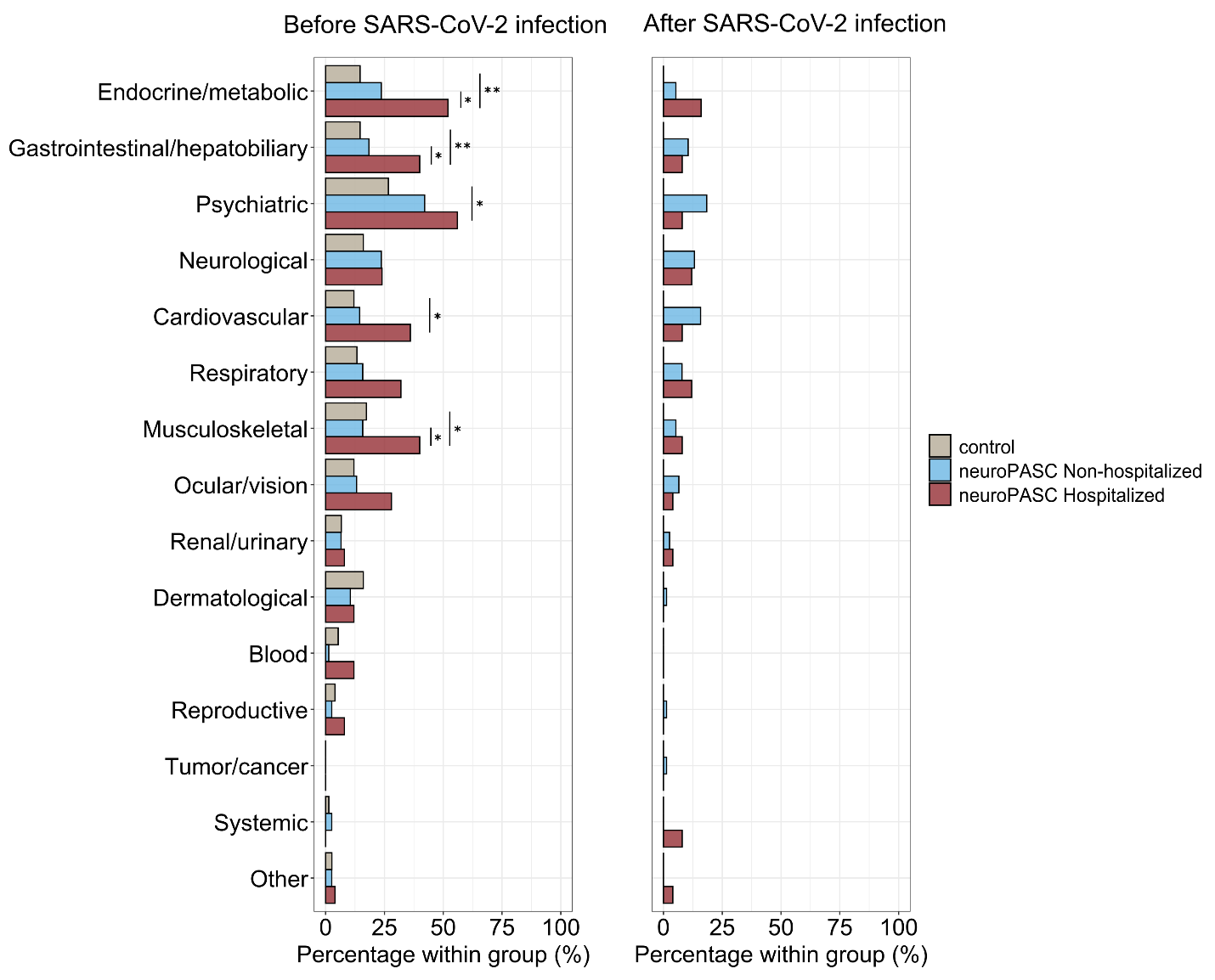


**Supplementary Figure 6** - Bar plot illustrating the percentage of participants with ongoing medical conditions (present at the time of the study visit) with onset prior to (left) or following (right) SARS-CoV-2 infection, non-hospitalized and hospitalized participants with neuroPASC and controls. Group differences were assessed using Fisher’s exact test per condition with false discovery rate adjusting for group testing (control vs neuroPASC non-hospitalized, control vs neuroPASC hospitalized and non-hospitalized vs hospitalized neuroPASC). *p<0.05, **p<0.01.


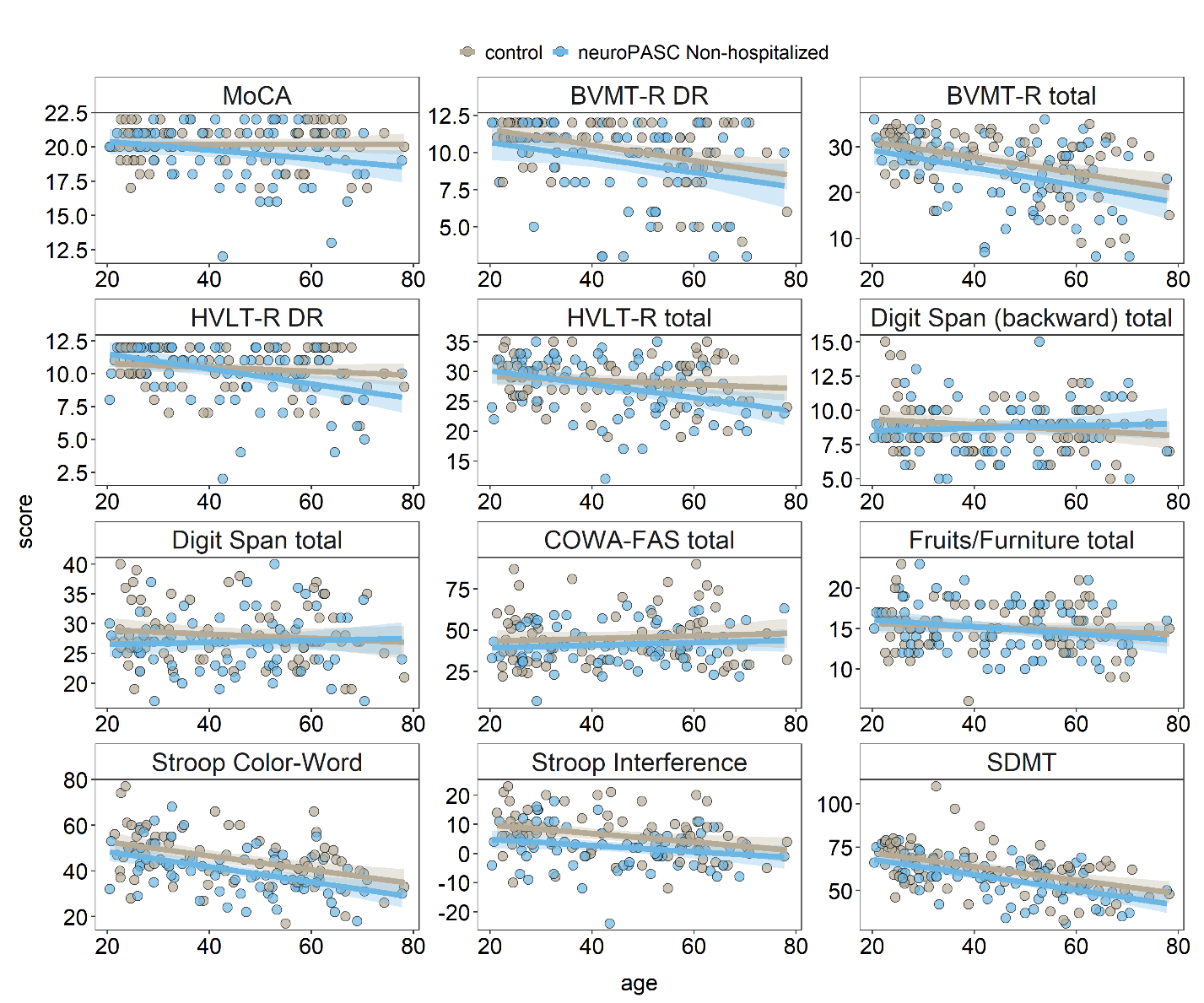


**Supplementary Figure 7** – Scatterplots showing linear regressions between age and cognitive scores in controls and non-hospitalized participants with neuroPASC. MoCA: Montreal Cognitive Assessment, BVMT-R: Brief Visuospatial Memory Test Revised, HVLT-R: Hopkins Verbal Learning Test- Revised, DR: delay recall, COWA-FAS: Controlled Oral Word Association Test, SDMT: Symbol Digit Modalities Test.


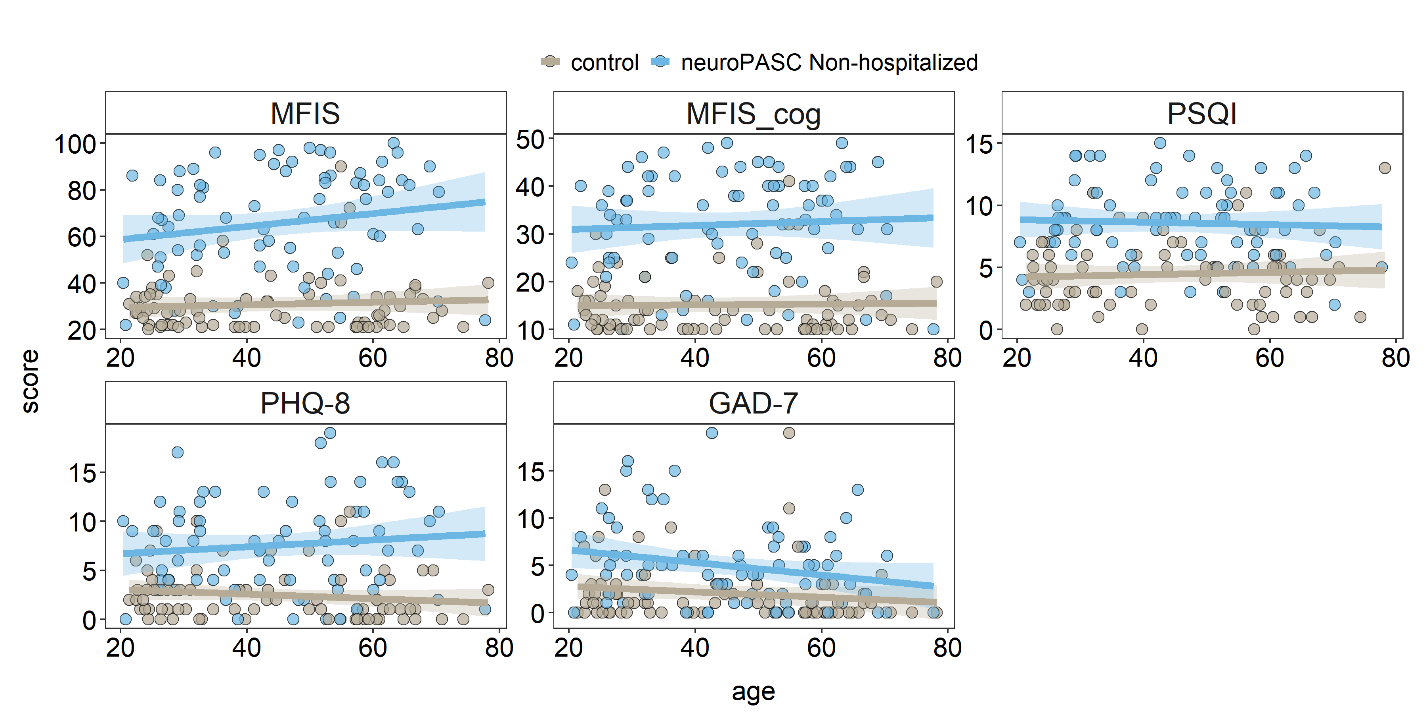


**Supplementary Figure 8** - Scatterplots showing linear regressions between age and quality-of-life scores in controls and non-hospitalized participants with neuroPASC. MFIS: Modified Fatigue Impact Scale, MFIS_cog: MFIS cognitive subscale, PSQI: Pittsburgh Sleep Quality Index, PHQ-8: Patient Health Questionnaire-8, GAD-7: General Anxiety Disorder-7 scale.


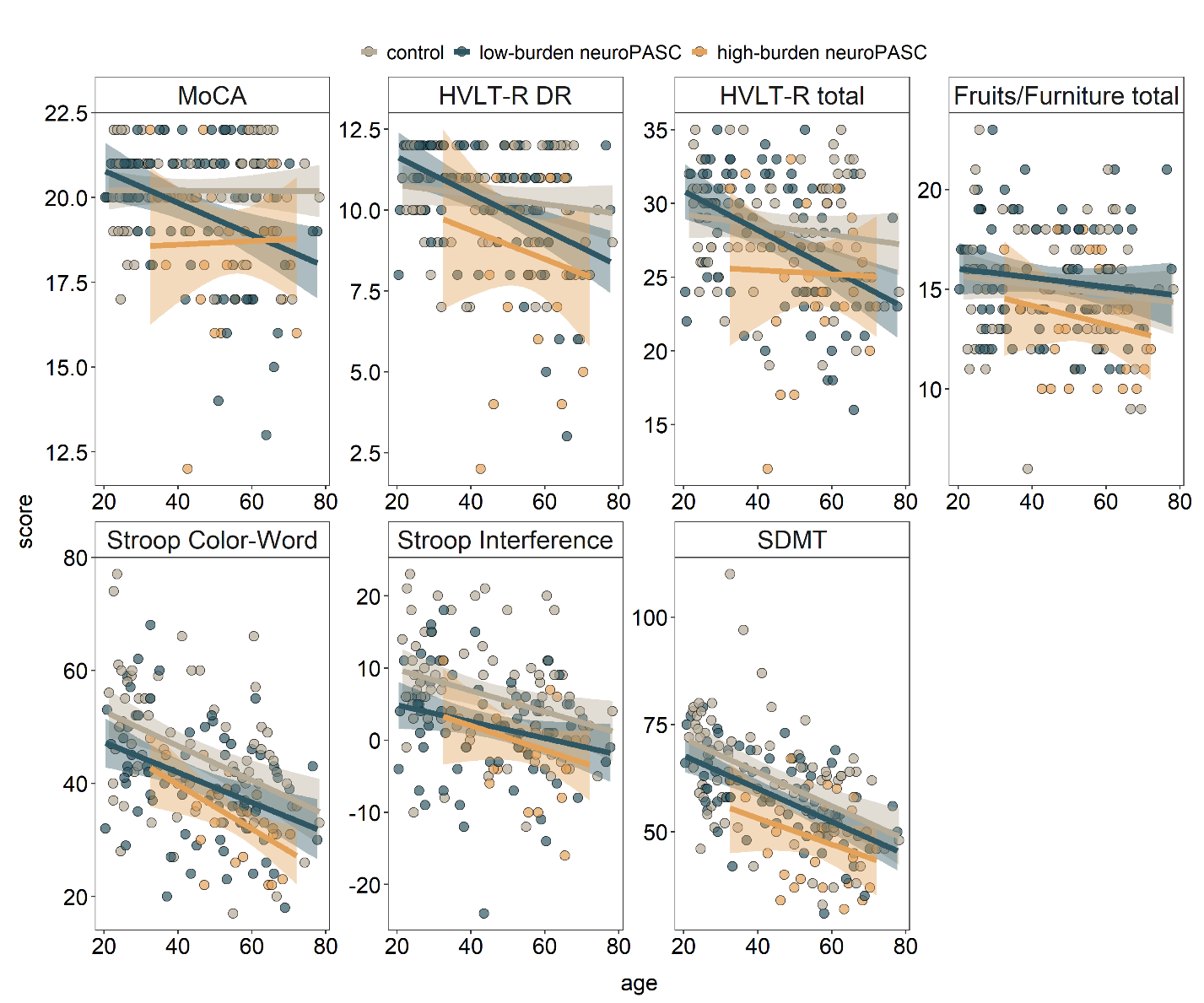


**Supplementary Figure 9** - Scatterplots showing linear regressions between age and cognitive scores in controls and in participants with low-burden or high-burden neuroPASC. MoCA: Montreal Cognitive Assessment, HVLT-R: Hopkins Verbal Learning Test- Revised, DR: delay recall, SDMT: Symbol Digit Modalities Test.


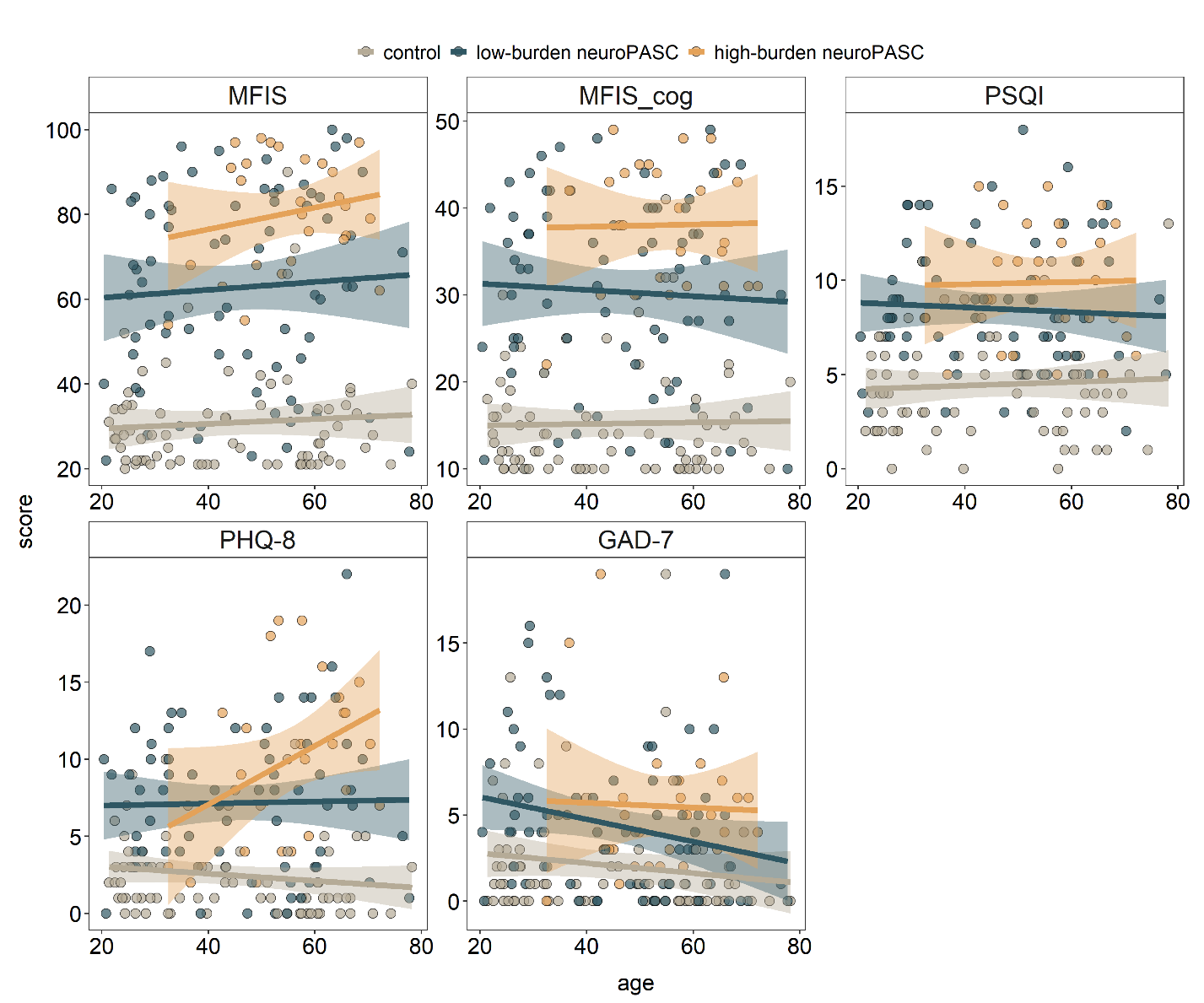


**Supplementary Figure 10 -** Scatterplots showing linear regressions between age and quality-of-life scores in controls and in participants with low-burden or high-burden neuroPASC. MFIS: Modified Fatigue Impact Scale, MFIS_cog: MFIS cognitive subscale, PSQI: Pittsburgh Sleep Quality Index, PHQ-8: Patient Health Questionnaire-8, GAD-7: General Anxiety Disorder-7 scale.
